# Systematic Modification of the Substitution Pattern of the 7-Hydroxy-5-oxopyrazolo[4,3-*b*]pyridine-6-carboxamide Scaffold Enabled the Discovery of New Ligands with High Affinity and Selectivity for the Cannabinoid Type 2 Receptor

**DOI:** 10.3390/molecules28134958

**Published:** 2023-06-24

**Authors:** Claudia Mugnaini, Magdalena Kostrzewa, Marta Casini, Poulami Kumar, Valeria Catallo, Marco Allarà, Laura Guastaferro, Antonella Brizzi, Marco Paolino, Andrea Tafi, Christelos Kapatais, Gianluca Giorgi, Federica Vacondio, Marco Mor, Federico Corelli, Alessia Ligresti

**Affiliations:** 1 Department of Biotechnology, Chemistry and Pharmacy, University of Siena, 53100 Siena, Italy; marta.casini94@gmail.com (M.C.); valeriacatallo@gmail.com (V.C.); guastaferro.laura@gmail.com (L.G.); antonella.brizzi@unisi.it (A.B.); paolino3@unisi.it (M.P.); andrea.tafi@unisi.it (A.T.); kapatais@student.unisi.it (C.K.); gianluca.giorgi@unisi.it (G.G.); federico.corelli@unisi.it (F.C.); 2 National Research Council of Italy, Institute of Biomolecular Chemistry, 80078 Pozzuoli, Italy; m.kostrzewka@gmail.com (M.K.); p.kumar@icb.cnr.it (P.K.); mallara@icb.cnr.it (M.A.); 3 Department of Food and Drug, University of Parma, Parco Area delle Scienze 27/A, 43124 Parma, Italy; federica.vacondio@unipr.it (F.V.); marco.mor@unipr.it (M.M.)

**Keywords:** cannabinoid type-2 ligands, receptor selectivity, structure–activity relationship, pyrazolo[4,3-*b*]pyridine derivatives

## Abstract

Selective ligands of the CB2 receptor are receiving considerable attention due to their potential as therapeutic agents for a variety of diseases. Recently, 7-hydroxy-5-oxopyrazolo[4,3-*b*]pyridine-6-carboxamide derivatives were shown to act at the CB2 receptor either as agonists or as inverse agonists/antagonists in vitro and to have anti-osteoarthritic activity in vivo. In this article, we report the synthesis, pharmacological profile, and molecular modeling of a series of twenty-three new 7-hydroxy-5-oxopyrazolo[4,3-*b*]pyridine-6-carboxamides with the aim of further developing this new class of selective CB2 ligands. In addition to these compounds, seven other analogs that had been previously synthesized were included in this study to better define the structure–activity relationship (SAR). Ten of the new compounds studied were found to be potent and selective ligands of the CB2 receptor, with *K*_i_ values ranging from 48.46 to 0.45 nM and CB1/CB2 selectivity indices (SI) ranging from >206 to >4739. In particular, compounds **54** and **55** were found to be high-affinity CB2 inverse agonists that were not active at all at the CB1 receptor, whereas **57** acted as an agonist. The functional activity profile of the compounds within this structural class depends mainly on the substitution pattern of the pyrazole ring.

## 1. Introduction

Primarily responsible for keeping body homeostasis, the endocannabinoid system (ECS) is a complex ubiquitous lipid signaling system consisting of two major G-protein-coupled receptors, cannabinoid-1 (CB1) and cannabinoid-2 (CB2) receptors, their endogenous ligands anandamide and 2-arachidonoylglycerol (2-AG), and the anabolic and catabolic enzymes responsible for their synthesis and degradation [1]. Although only CB1R and CB2R are widely recognized as CBRs, many other receptors, from other GPCRs to ion channels and nuclear receptors, have been reported to interact with cannabinoids [2]. This pleiotropic system plays a critical role in regulating a variety of physiological processes and functions, including mood regulation, nociception, cognitive functions, motivation and reward, appetite, lipid and glucose metabolism, neurogenesis and neurodegeneration, inflammation, immune functions, smooth muscle contractility, and cell growth and proliferation. The pathophysiological relevance of the endocannabinoid system has been demonstrated in a variety of diseases including pain, neurodegenerative diseases, seizures, psychiatric disorders, obesity, metabolic diseases, and cancer [2]. Thus, direct targeting of the receptors as well as indirect targeting via modulation of the endogenous machinery involved in this system can provide a successful therapeutic outcome [3]. Nevertheless, manipulating such a high-complex system can be particularly challenging when we consider possible multidirectional effects that could be triggered by affecting other mediators that are biochemically related to endocannabinoids [1].

CB1 and CB2 receptors share 44% amino acid homology and differ in their localization in the human body [3]. The CB1 receptor is one of the most abundant GPCRs in the brain, and this distribution correlates with its role in controlling motor function, cognition and memory, and pain relief [4]. The CB2 receptor is predominantly expressed in immune cells and moderately expressed in other peripheral tissues, including the cardiovascular system, gastrointestinal (GI) tract, liver, adipose tissue, bone, and reproductive system [5]. Although the expression of the CB2R in the CNS is limited, it is undeniable that CB2 activation is associated with neurological activities such as nociception, drug addiction, neurodefense mechanisms, and reduction in neuroinflammation [6].

Over the years, a large number of synthetic ligands have been identified that can act at both cannabinoid receptors. Recently, crystal structures of both receptors have been solved showing that they have a very similar lipophilic orthosteric agonist binding pocket, which poses challenges for the development of selective agonists [7,8,9,10]. Moreover, at least three structural states have been identified for the CB receptors, namely, inactive, active-like (intermediate), and active, indicating complex mechanisms underlying receptor activation and signal transduction [11].

Among synthetic molecules, CB2 ligands have received considerable attention due to their ability to activate the receptor without eliciting the psychotropic effects typical of CB1 stimulation [12,13]. Although no selective CB2 ligand has yet been approved for commercialization, CB2 activation has so far shown considerable preclinical and clinical interest for the treatment of autoimmune diseases and disorders with an inflammatory component [14,15,16,17,18,19,20], cancer [21,22], and metabolic disorders [23]. On the other hand, CB2 antagonists and inverse agonists have previously been considered as pharmacological tools for immunomodulation, but this area of research needs further exploration from a therapeutic perspective [24,25].

**Figure 1:**
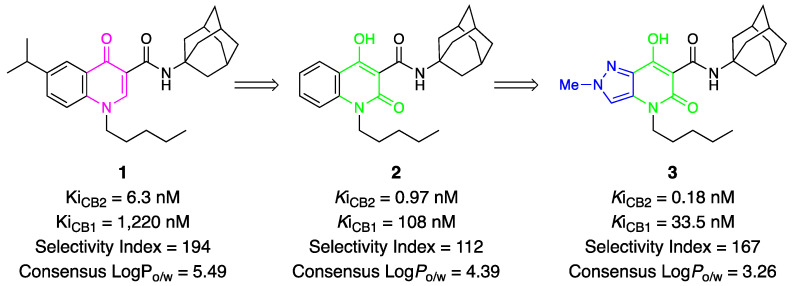
Schematic representation of the structural evolution of CB2 ligands studied by our research group.

Our research team has long been actively engaged in the search for new CB2 ligands with improved affinity and selectivity on the one hand, and physicochemical properties compatible with the criteria of pharmaceutical development on the other. This activity started with the synthesis and pharmacological evaluation of compounds (Figure 1) with a 4-quinolone structure (e.g., **1**) [26,27,28,29,30,31,32], then progressed to 2-quinolone structures (e.g., **2**) [33], and finally reached pyrazolo[4,3-*b*]pyridine derivatives (e.g., **3**) [34], constantly improving their pharmacological and physicochemical profile as CB2 ligands. Overall, the type **3** compounds proved to be the most balanced, as they combined all the desired properties and were better on average.

**Figure 2:**
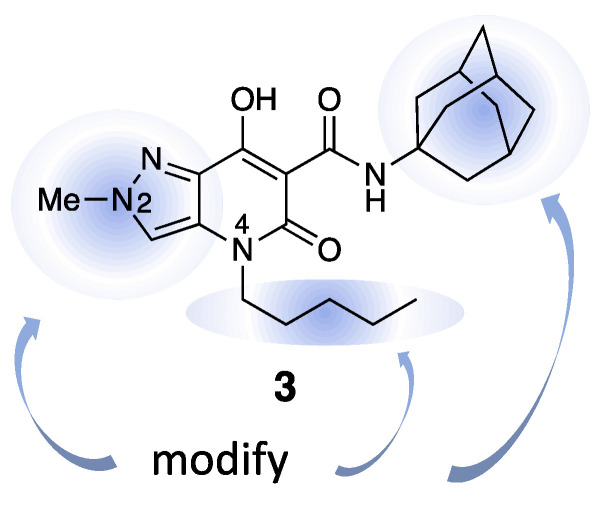
Schematic representation of the design strategy.

Following these results, a more detailed investigation of pyrazolo[4,3-*b*]pyridine compounds was carried out by extending the structure–activity relationship (SAR). Starting from structure **3**, a small library of 23 compounds was created by changing three parts of the molecule, namely, the amide substituent, the chain at position 4, and the substitution pattern of the pyrazole ring, as well as their combinations. In particular, the design strategy for the new compounds, summarized in Figure 2, was aimed at (*i*). investigating the effects on activity associated with less bulky aliphatic or planar aromatic amide substituents, chains at N4 with a smaller number of rotatable bonds; (*ii*). the introduction of substituents, also different from methyl, at other positions on the pyrazole ring; and (*iii*). the evaluation of their solubility in water after conversion to salts with monovalent cations. The results of these studies are reported herein.

## 2. Results and Discussion

### 2.1. Synthesis

Most of the new compounds were prepared by synthetic methods that we have already described for similar compounds [34].

**Scheme 1:**
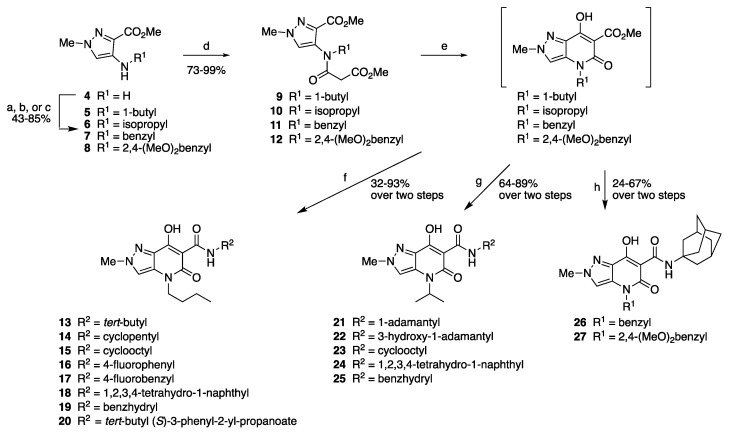
Synthesis of the final compounds **13**–**27**. *Reagents and conditions*: (a) 1-iodobutane, DMF, 50 °C, 7 h; (b) 2-iodopropane, K_2_CO_3_, AcCN, reflux, 16 h; (c) aromatic aldehyde, Na_2_SO_4_, dry DCM, rt, overnight, then NaBH_4_, rt, 2 h; (d) methyl malonyl chloride, TEA, dry DCM, 0–5 °C, 2–6 h; (e) NaH, THF, MeOH (cat.), 60 °C, 2 h; (f) appropriate amine, toluene, reflux, 1–2 h; (g) appropriate amine, toluene, reflux, 1–2 h; (h) 1-adamantylamine, toluene, reflux, 2 h.

Briefly, a first set of 15 compounds showing modifications either at the amide moiety or at the N4 chain or at both positions was prepared starting from methyl 4-amino-2-methylpyrazole-3-carboxylate (**4**) [35] (Scheme 1), which was *N*-alkylated by reaction with appropriate alkyl iodides or by reductive alkylation with aromatic aldehydes to give intermediates **5**–**8**, which were then *N*-acylated with methyl malonyl chloride to give bicyclic precursors **9**–**12**. These were converted by Dieckmann condensation into methyl 4,5-dihydro-7-hydroxy-5-oxo-2*H*-pyrazolo[4,3-*b*]pyridine-6-carboxylates, which were directly subjected to amidation reaction with the corresponding amines without purification and/or characterization to afford the final compounds **13**–**27**.

**Scheme 2:**
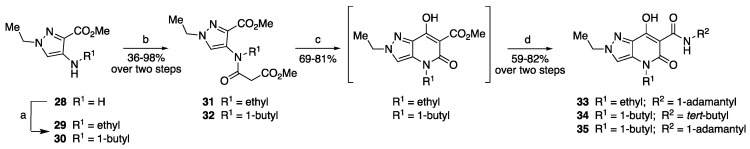
Synthesis of the final compounds **33**–**35**. *Reagents and conditions*: (a) 1-iodoethane or 1-iodobutane, DMF, 50 °C, 7 h; (b) methyl malonyl chloride, TEA, dry DCM, 0–5 °C, 2–6 h; (c) NaH, THF, MeOH (cat.), 60 °C, 2 h; (d) appropriate amine, toluene, reflux, 1–2 h.

Following the same procedure, the aminopyrazole derivative **28** [36] (Scheme 2) was converted by *N*-alkylation to intermediates **29**, **30**. Reaction with methyl malonyl chloride gave the *N*-acylated products **31**, **32**, which, in turn, were cyclized to 4,5-dihydro-7-hydroxy-5-oxo-2*H*-pyrazolo[4,3-*b*]pyridine-6-carboxylates. Subsequent reaction with the appropriate amines led to the final compounds **33**–**35**, which carried modifications of the amide or chain substituents, in all cases linked to a group other than methyl at the N2 position.

**Scheme 3:**
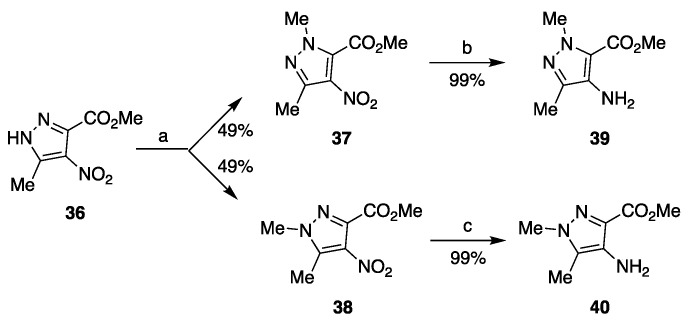
Synthesis of isomeric intermediates **39** and **40**. *Reagents and conditions*: (a) (MeO)_2_SO_2_, K_2_CO_3_, acetone, rt, overnight; (b) HCO_2_NH_4_, Pd/C, EtOH/H_2_O, 50 °C, 2–3 h; (c) HCO_2_NH_4_, Pd/C, EtOH/H_2_O, 50 °C, 2–3 h.

**Scheme 4:**
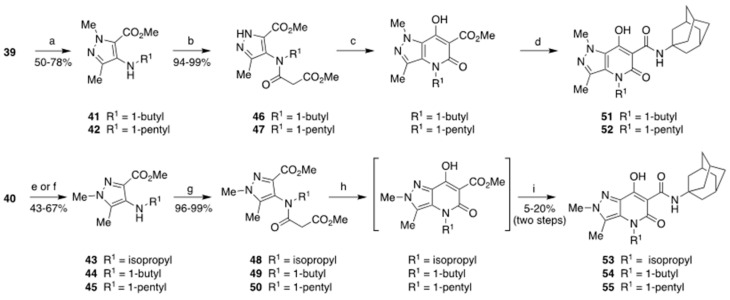
Synthesis of the final compounds **51**–**55**. *Reagents and conditions*: (a) 1-iodobutane or 1-iodopentane, DMF, 50 °C, 7 h; (b) methyl malonyl chloride, TEA, dry DCM, 0–5 °C, 2–6 h; (c) NaH, THF, MeOH (cat.), 60 °C, 2 h; (d) 1-adamantylamine, toluene, reflux, 2 h; (e) 1-iodobutane or 1-iodopentane, DMF, 50 °C, 7 h; (f) 2-iodopropane, K_2_CO_3_, AcCN, reflux, 16 h; (g) methyl malonyl chloride, TEA, dry DCM, 0–5 °C, 2–6 h; (h) NaH, THF, MeOH (cat.), 60 °C, 2 h; (i) 1-adamantylamine, toluene, reflux, 2 h.

With the aim of deepening the understanding of SAR in the class of 7-hydroxy-3-methyl-5-oxopyrazole[4,3-*b*]pyridine-6-carboxamide compounds (as **3**), we set out to investigate the previously unexplored chemical space around position 3 by replacing the hydrogen atom with a methyl group. To this end, compound **36** (Scheme 3), intended as the starting point for the synthesis, was prepared according to a previous literature report [37], but then subjected to *N*-methylation according to a different and more convenient approach, since we found that treatment at room temperature with dimethyl sulfate in the presence of potassium carbonate in acetone resulted in obtaining the two isomeric products **37** and **38** in the same yield of 49% each after separation of the reaction mixture by flash chromatography. The structure of both regioisomers **37** and **38** was elucidated using ^1^H NMR data. These nitro derivatives were reduced with ammonium formate in the presence of 10% Pd/C to give the amino compounds **39** and **40**, which, in turn, could be processed in parallel (Scheme 4) by the same method shown in Scheme 1 and Scheme 2.

Thus, **39** and **40** were alkylated to give the secondary amine derivatives **41**–**45**, which, after amidation to give **46**–**50** with methyl malonyl chloride, underwent Dieckmann cyclization to yield the bicyclic ester compounds, whose direct treatment with the appropriate amines led to the final compounds **51**–**55**. However, while compounds **51** and **52** were obtained in two steps with a moderate yield of 45–47%, compounds **53**–**55** were obtained only with a low yield of 5–20%. This difference in behavior could be justified by the fact that in compounds **53**–**55,** three substituents (i.e., the two methyl groups and the aliphatic chain in position 4) are in close proximity to each other and in a coplanar arrangement, leading to repulsive steric interactions. Therefore, the Dieckmann cyclization reaction is thermodynamically less favored when applied to substrates **48**–**50** than to substrates **46** and **47**. X-ray crystallographic analysis [38,39] of compound **51** (see Supplementary Information, Figure S1: X-ray crystal structure of compound **51**) confirmed that the structural assignment performed by ^1^H NMR was correct.

Finally, a series of compounds characterized by an adamantylamide and an isopropyl chain at position 4, but having different substituents at positions 1 or 2 of the pyrazole ring, was synthesized by a modified procedure recently described by us [40]. This synthetic approach has allowed the chemical diversity around the pyrazole moiety to increase by introducing substituents at positions 1 or 2 as the final step of the synthesis. The structure of compounds **56**–**62** is highlighted in Table 1, along with the structure of all the other final pyrazolo[4,3-*b*]pyridine-6-carboxamide derivatives that have been the subject of subsequent pharmacological investigation.

### 2.2. Estimated Drug-like Properties

There is a growing interest in making the drug discovery process more effective by incorporating the most favorable physicochemical properties (e.g., molecular weight, solubility, polar surface area, ClogP, and others) early in the design and evaluation of new chemical entities to increase the likelihood of further preclinical/clinical development [41,42]. Among all synthesized compounds, we selected those (**15**, **18**, **26**, **35**, **54**, and **55**) with higher receptor affinity, i.e., compounds with *K*_i_CB2 < 20 nM (see Table 1). Their physicochemical and pharmacokinetic parameters were evaluated using the free web tool SwissADME [43,44]. All compounds showed a very favorable overall profile, as they did not violate Lipinski’s “rule of five” in any way and showed no potential as PAINS (see Supplementary Information, Table S1. Calculated physicochemical and drug-like properties of selected compounds). The same compounds were also characterized by a multi-parameter score LLE (Liphophilic Ligand Efficiency) within the optimal range for suitable drug candidates (5 < LLE < 7) [45,46], apart from **18** (LLE = 4.90), since the reduction in overall lipophilicity, as predicted by CLogP values, did not affect the CB2R affinity values (p*K*_i_CB2).

### 2.3. Water Solubility Evaluation

The results of drug discovery and development can be significantly affected by the poor water solubility of pharmaceutical compounds. The solubility of the drug is mainly influenced by its chemical disposition and the conditions of its solution. The molecular assembly defines the molecular volume, crystal energy, hydrogen bonding, ionizability, and lipophilicity that determine the solubility of the drug [47].

**Figure 3:**
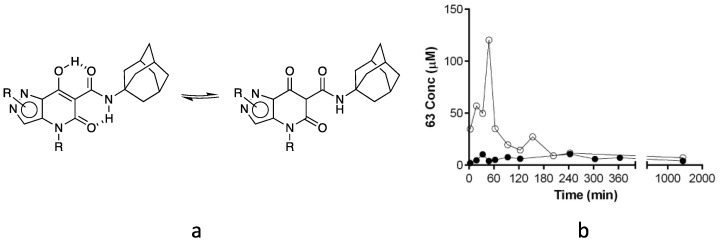
(**a**) Tautomeric equilibrium and H-bond networking. (**b**) Plot of concentration (µM) as a function of time (min) for **63**-sodium (filled circles) and -cesium (void circles) salts in a 0.9% NaCl solution.

7-Hydroxy-5-oxopyrazolo[4,3-*b*]pyridine-6-carboxamide derivatives typically have pKa values in the range of 4.2–5.0 [48], and their salts are expected to be sufficiently soluble in water at physiological pH, while the tautomeric equilibrium in the neutral species (Figure 3a) results in the formation of a strong intramolecular hydrogen bonding network that likely reduces its solubility.

Therefore, despite their lower lipophilicity compared to type **1** compounds (Figure 1), the thermodynamic water solubility of type **3** compounds as well as their dissolution rate is expected to be poor/moderate and a limiting factor for their evaluation in in vitro/in vivo tests. Therefore, we decided to investigate the dissolution of different salts (Na^+^, K^+^, Cs^+^, and Me_4_N^+^) of **63** (Table 1), representative of the whole class of compounds tested, testing their solubility in different EtOH/water mixtures. The **63**-cesium salt (85.7 mg in 1 mL) proved soluble in a 1:20 EtOH/water mixture, which is approximately equivalent to a 6% *v*/*v* solution of EtOH in water. The potassium salt was soluble in a 1:4 EtOH/water mixture, while the sodium salt began to precipitate once the 1:1 ratio of the same mixture was reached. The tetramethylammonium salt was not soluble at all, probably due to its organic nature. These results confirmed the idea that cations with progressively larger ionic radius (sodium < potassium < cesium) can lead to progressively less associated ion pairs with the **63**-enolate and, as expected, **63**-salts with larger cations showed increased solubility in solvent mixtures with low EtOH content, where salt dissociation predominates and the neutral form is not solubilized by the organic co-solvent.

Moreover, the solubility of the **63**-sodium salt in physiological 0.9% NaCl solution, which is the delivery vehicle for in vivo administration, is strongly affected by the action of the common ion (Na^+^), resulting in a further reduction in solubility. Plotting the concentration (µM) at different time points of dissolved **63**, added to a physiological solution as an excess of the sodium salt (Figure 3b), we observed relatively constant values with an average of 6.37 × 10^−6^ mol/L. Considering the concentration of sodium ions (154 mM), the solubility product constant for the sodium salt results: Ks = [Na^+^] [**63**^−^] = 9.81 × 10^−7^ mol^2^/L^2^. When added to the same solution, **63**-cesium salt initially showed higher concentrations of dissolved **63**, which gradually decreased reaching, after 200 min, the value consistent with the Ks of the sodium salt and the excess of sodium ions. However, starting from the cesium salt, the concentration of drug dissolved in the physiological solution maintained high values for at least one hour (around 50 × 10^−6^ mol/L, with a peak higher than 100 × 10^−6^ mol/L) and, for a further two hours, these values were higher than those obtained with the sodium salt. From this, it can be deduced that the **63**-cesium salt would be very suitable for the extemporaneous preparation of injectable solutions in the presence of high concentrations of sodium ions.

### 2.4. In Vitro Pharmacology

The binding affinities (*K*_i_ values) of the new compounds for human recombinant CB1R and CB2R are reported in Table 1. The compounds were evaluated in parallel with compounds **63** and **3** already reported [34] and SR144528 as reference CB2R ligand, as previously described ([34] and references cited therein). Among the thirty compounds tested, fourteen of them displayed significant binding affinity for CB2R, with *K*_i_ values < 200 nM, while twelve of these proved to be potent CB2R ligands with *K*_i_ values in the range 0.45–61.76 nM. Only compounds **26** and **35** showed also significant affinity for CB1R, with *K*_i_ values of 115.63 and 102.30 nM, respectively. Nevertheless, all the compounds with affinity for the CB2R had selectivity index values between >70 and >4739.

**Figure 4:**
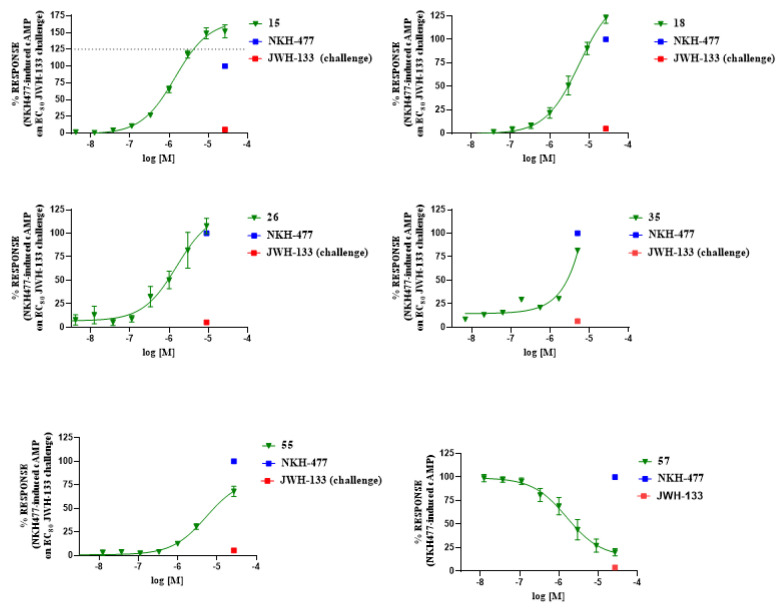
Concentration–response curves of compounds **15, 18, 26, 35, 55,** and **57** in a cAMP-based functional assay. The curves show the effect of increasing concentrations of ligands on NKH477-induced cAMP levels in stable CHO cells expressing the human recombinant CB2 receptor.

To investigate the effects of key substitutions at the pyrazolo[4,3-*b*]pyridine scaffold on functional activity at CB2 receptors, the ability of representative high-affinity and selective compounds (**15**, **18**, **26**, **35**, **55,** and **57**) to modulate intracellular cAMP levels in NKH-477-stimulated CHO cells—optimized to overexpress CB2R—was measured using enzyme fragment complementation technology. As shown in Figure 4, only compound **57** activated the receptor with typical agonist behavior (EC_50_ = 1.59 µM, Emax = 84.05) by reducing cAMP levels induced by NKH-477 (a water-soluble analog of forskolin) as expected for a Gi protein-coupled receptor agonist. All the other compounds showed a classical antagonist/inverse agonist behavior as suggested by their capability of antagonizing the CB2-ligand challenge (4 µM, JWH-133) and, in the case of **15** and **18,** of increasing the level of NKH-477-induced cAMP (Figure 4).

### 2.5. Structure–Activity Relationship (SAR)

A crucial effect on receptor affinity was demonstrated by modifications in the amide function (Table 1). While the rest of the structure remained essentially unchanged, compounds **13**, **14**, and **17**, in which the *N*-adamantyl group was replaced by aliphatic groups or groups containing an aromatic ring, were without activity up to a concentration of 10,000 nM. However, the introduction of benzhydryl, tetrahydronaphthalene-1-yl, and cyclooctyl groups resulted in compounds **19**, **18**, and **15**, respectively, showing gradually decreasing *K*_i_ values for the CB2 receptor, with the last two compounds (especially **15**) proving to be potent and selective ligands. Apparently, the cyclooctyl system is able to reproduce quite well the properties of an adamantyl group in terms of lipophilicity and steric requirements, while this result cannot be achieved when smaller aliphatic groups (compounds **13** and **14**) or an extended aromatic system (e.g., **19**) are inserted at the amide nitrogen. However, any modification of the amide moiety appears to be completely ineffective in providing pharmacological activity when combined with the insertion of a short aliphatic chain (namely, isopropyl) at the N4 position, as in the case of compounds **21–25**, which are completely inactive even when the adamantyl group is retained (e.g., **21** and **22**). Surprisingly, the presence of a benzyl group at N4 makes compound **26** one of the most potent CB2 ligands among the 30 compounds studied, with also nonnegligible selectivity toward the CB1 receptor (SI = 241). Such a striking effect on pharmacological activity, achieved by replacing an *n*-butyl chain with a benzyl group, might be the consequence of a moderate increase in lipophilicity and a concomitant certain decrease in the conformational freedom of the molecule. On the other hand, caution should be exercised in interpreting this experimental result because the introduction of a 2,4-dimethoxybenzyl group in place of the simple benzyl leads to the completely inactive compound **27**.

The application of two simultaneous structural modifications (compounds **33**–**35**) gave positive results. For example, **34** with an ethyl group at N2 and a *tert*-butylamide showed an affinity for the CB2 receptor increased by two orders of magnitude compared to compound **13**, whereas in the case of **33**, with an ethyl substituent at both the N2 and N4 positions, the affinity decreased by an order of magnitude but was accompanied by a significant selectivity advantage over **63**. The third compound in this subgroup, namely, **35**, which differs from compound **63** in having an ethyl instead of a methyl at N2, resulted in even better binding to both CB1 and CB2 receptors. Of all the compounds tested, compound **35** can be considered the best in terms of CB2 affinity. Comparing its in vitro activity with that of **3** and **63**, compound **35** actually falls between **3** and **63** in terms of CB1 and CB2 affinity. The lengthening of the N2 substituent in **35** from one to two carbon atoms seems to partially compensate for the shortening of the N4 chain by one carbon atom during the transition from **3** to **63**.

Very interesting results were obtained with the introduction of another methyl group in position 3 of the pyrazole ring. Although the length (four or five carbon atoms) of the linear chain in the N4 position seems to be irrelevant, the relative position of the two methyl groups has a very significant effect. While the simultaneous presence of two methyl groups at N1/C3 (compounds **51** and **52**) produces ligands that are sufficiently potent but much more selective than the analogous compounds methylated only at the N1 position [34], the combination of two methyl groups at N2/C3 (compounds **54** and **55**) maintains affinity for the CB2 receptor (cf. **63**), but completely abolishes the ability to interact with the CB1 receptor, with the respective SI values exceeding 4600. The last compound **53**, which has two N2/C3 methyl groups and the isopropyl chain in the N4 position at the same time, proved to be inactive, as expected based on the results described above for compounds **21**–**25**. Nevertheless, it should be borne in mind that the overall pharmacological profile of a given molecule is probably due to the combined effects of the various substituents present rather than to the action of any single one of them. This is indeed evident in the case of the subgroup of compounds **56**–**62**: all N4-isopropyl-substituted compounds that do not have substituents in the N1 position of the pyrazole ring (namely, **56**, **59**, **61**, and **62**) are inactive, as expected. In contrast, the compounds (**57**, **58**, and **60**) that are N1-substituted with a methyl, propargyl, or cyanomethyl group, respectively, all have pharmacological activity that is far from negligible and comparable among themselves.

The results of the functional activity assay are consistent with the presence of a substituent on the pyrazole ring at the N1 position (for agonists) or at the N2 position (for antagonists), independently of its nature (methyl, ethyl, or other chains). The introduction of a second methyl group into the pyrazole ring (compound **55**) or even a significant change at N4 of the pyrazolo[4,3-*b*]pyridine system (compound **26**) confirmed or enhanced the antagonistic effect as suggested by the increased levels of NKH-477-induced cAMP upon a challenge with an EC_80_ concentration of the CB2 agonist JWH-133. Similarly, we observed that compounds **15** and **18**, whose adamantyl group was replaced by different cycles, were still able to antagonize JWH-133-induced inhibition of NKH-477-induced cAMP formation.

### 2.6. Molecular Modeling

**Figure 5:**
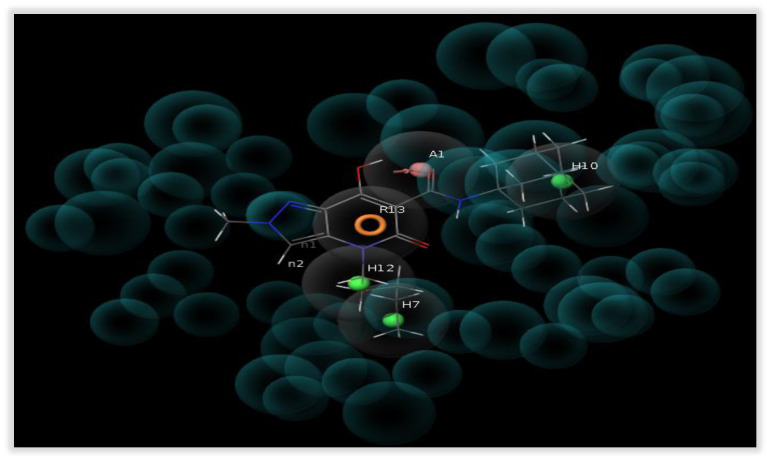
Graphical representation of the interaction pattern between one of the most active compounds (compound **3** [30], *K*_i_(CB2) = 0.18 nM) used in the development of the 3D-QSAR model (Training set) and the five feature CB2 structure-based pharmacophoric model.

Previously, a CB2 3D-QSAR affinity model had been developed [29], which led to the identification of a new CB2 receptor ligand with high affinity and selectivity [30]. In this study, we extended the 3D-QSAR model by creating a structure-based CB2 pharmacophore that comprised the excluded volumes (Figure 5) and by introducing new compounds to increase the predictability and accuracy of the 3D-QSAR model. The structure of the CB2 receptor co-crystallized with AM-10257 (Protein Data Bank code: 5ZTY, resolution: 2.80 Å) was selected and prepared for calculations using Protein Preparation Wizard to assign bond orders, add missing hydrogen atoms, cap termini, and generate tautomeric states.

The structure-based CB2 pharmacophore was used for further 3D-QSAR analysis. The atom-based 3D-QSAR model was constructed using Phase’s Partial Least Square (PLS) statistical analysis. To develop the final 3D-QSAR model, 155 CB2 ligands were selected regardless of their functional activity. The molecules used to construct the 3D-QSAR CB2 affinity model were derivatives of different structural classes, in particular 7-hydroxy-5-oxopyrazole[4,3-*b*]pyridine-6-carboxamide [34], tetrahydrobenzo[*b*]thiophenes, 2-phenylthiophenes, *N*-phenylpyrroles and pyrroles [49], 2-alkylthieno[2,3-*c*]thiopyran-3-carboxylate, pyridines and isoxazoles [50], 1-phenylpyrazoles and pyrazoles [50,51], 1,3-benzothiazoles and thiazoles [49], 1*H*-benzimidazoles [52], imidazoles [53], diphenylpurines [54], dibenzopyrans [55,56,57], indoles [58,59,60], 4-quinolone-3-carboxylic acids [28,61,62], phenylsulfonamides [63,64], hexahydrocycloocta[*b*]thiophene [62], 2-phenylbenzofuran [63], decahydrotetraphene [64], CP-55,940 [28], WIN 55,212-2 [65], and 5-pentyl-2-(2-phenylpropan-2-yl)-2,5-dihydro-1*H*-pyrido[4,3-*b*]indol-1-one [66]. The binding affinities of these compounds were measured using the same protocol, i.e., displacement of the radioligand [3*H*]-(-)-*cis*-3-[2-hydroxy-4-(1,1-dimethylheptyl)phenyl]-trans-4-(3-hydroxypropyl)cyclohexanol, also known as [3*H*]-CP-55.940, assuming that they bind to the same receptor binding pocket. The range of binding affinity of the ligands spans five orders of magnitude, an important requirement for the development of a statistically significant and stable 3D-QSAR model. 

The entire set of ligands, which consisted of 155 molecules, was randomly divided into a training set and a test set, which contained 95 and 60 compounds, respectively. The constructed 3D-QSAR model was tested using two independent test sets of derivatives, and it was decided that it could be used for the development of new high-affinity CB2 compounds. The 3D-QSAR contour plots were analyzed to understand the effects of the spatial arrangement of the structural features. By default, the blue cubes indicate favorable features that contribute to ligand interactions with the CB2 receptor, while the red cubes indicate unfavorable features.

**Figure 6:**
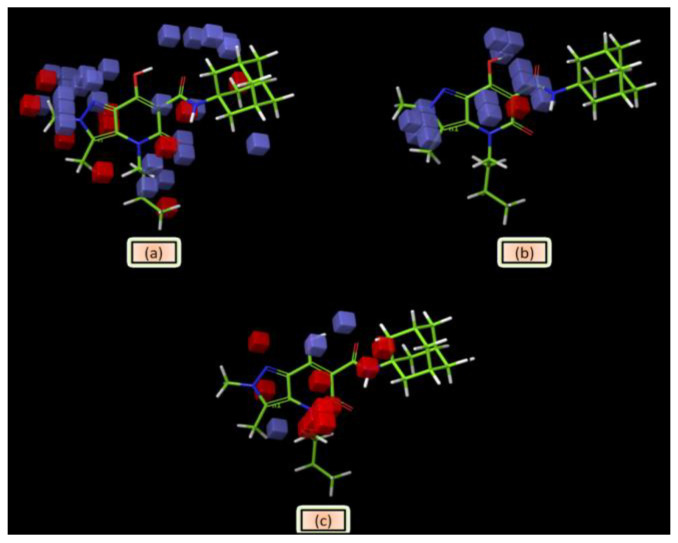
Pictorial representation of the contour plots generated for compound **54** in the context of (**a**) hydrophobic/non-polar interactions, (**b**) hydrogen bond donor effects, and (**c**) electron-withdrawing groups.

According to our predictions, the alkyl chain at N4 had a major effect on receptor affinity. The presence of blue cubes around the alkyl chain at N4 (Figure 6a) indicates the preference of hydrophobic/nonpolar functional groups at this position. Moreover, the substitution of the alkyl chain at N4 with an isopropyl group leads to a decrease in binding affinity due to the shortness of this group. The presence of red cubes around the alkyl chain suggests that further elongation could decrease the binding affinity at the CB2 receptor.

Indeed, the alkyl chain was not further elongated to avoid an excessive increase in lipophilicity. According to our contour analysis, the substitution of the alkyl chain at N4 with a benzyl group could lead to high-affinity compounds (see compounds **26** and **3**), which was confirmed by the experimental results. Surprisingly, this benzyl group also increased the selectivity toward the CB2 receptor. Another result of our 3D-QSAR contour plot analysis was the N1, N2, and N3 methylation of the pyrazolopyridine moiety. Methylation of these positions could lead to high-affinity compounds. The presence of red cubes next to the methyl groups at positions N1, N2, and N3 indicated that more than one methyl group in this region of the molecule could decrease the binding affinity at the CB2 receptor, which was also confirmed by the experimental results (Figure 6a). The blue cubes around the methyl groups indicate that the substituents in these positions may interact as H-bond donors in the CB2 binding pocket (Figure 6b). The appearance of blue cubes around the hydroxyl group in position 7 and the oxygen of the amide moiety in position 3 (Figure 6c) of the pyrazolopyridine core indicates the preference of electron-withdrawing groups at these positions. On the other hand, the red cubes around the alkyl chain indicate that the electron-withdrawing groups are not preferred in this position.

**Figure 7:**
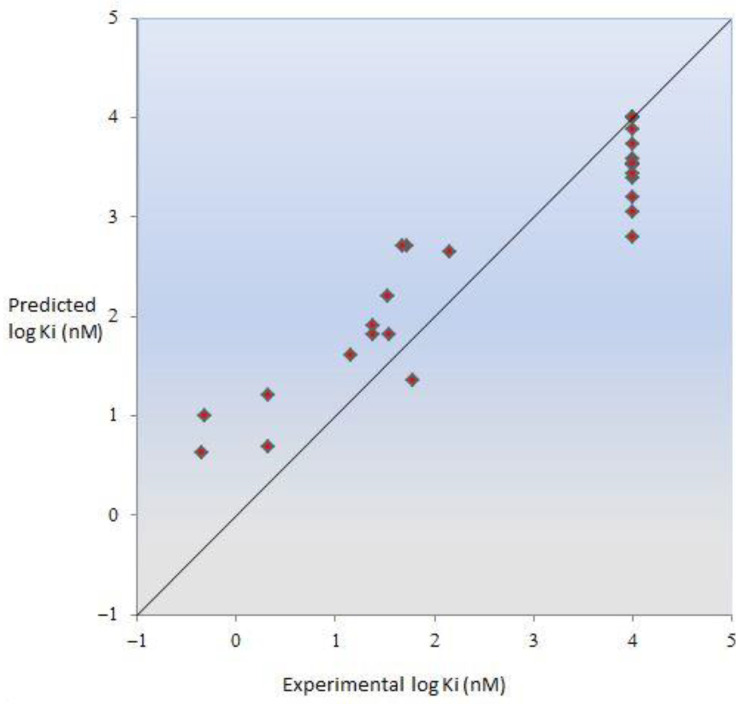
Plot of predicted vs. experimental CB2 receptor affinity. The log*K*_i_ (nM) values are reported in Table 1.

At this point, we decided to compute the binding affinity of the new CB2 compounds mentioned in this article to obtain some information before planning the synthesis of further series of compounds. The experimental results herein reported were in agreement with our predictions (Figure 7) (further details on the model will be reported elsewhere in due time). These findings on the predictive ability of our newly developed computational approach will allow us to further rationalize the structure–affinity relationship through its microscopic interpretation in view of the design of other CB2 ligands.

## 3. Materials and Methods

### 3.1. Synthesis

Commercially available reagents were used as received unless otherwise specified. Solvents were treated before use with suitable drying agents and used after distillation in an atmosphere of N_2_. Reactions requiring anhydrous conditions were performed under N_2_. Organic solutions were dried over anhydrous Na_2_SO_4_. Evaporation was carried out at reduced pressure using a rotating evaporator. Melting points were determined on a Gallenkamp apparatus and are uncorrected. For flash chromatography, Merck Kieselgel 60 (0.040–0.063 mm) was used. Thin layer chromatography (TLC) was performed on silica gel plates (Merck 60 F254) eluting with solvents indicated, visualized by a 254 nm UV lamp (λ = 254 nm), and stained with aqueous KMnO_4_, Pancaldi, and phosphomolybdic acid solutions. ^1^H NMR and ^13^C NMR spectra were recorded on a Bruker Avance DPX-400 NMR spectrometer at 400 MHz (^1^H) or 100 MHz (^13^C), respectively, or on a Bruker Avance DRX-600 spectrometer operating at 600 MHz (^1^H) or 150 MHz (^13^C), respectively. Mass spectra were recoded with an LC-MSD 110 series AGILENT instrument, with electrospray interface and 0.4 mL/min flow rate using a binary solvent system of 95:5 methanol/water. The UV detector was set at 254 nm and mass spectra were acquired either in positive or in negative mode scanning over the mass range of 105–1500. Elemental analyses were performed in our analytical laboratory and agreed with theoretical values to within ±0.4%.

#### 3.1.1. General Procedure for the Synthesis of Compounds **5**, **29**, **30**, **41**, **42**, **44**, **45**

The appropriate primary alkyl halide (2.26 mmol) was added to a solution of primary amine derivative **4**, **28**, **39**, **40** (2.26 mmol) in dry DMF (3 mL) under a nitrogen atmosphere. The solution was gradually warmed to 50 °C and maintained at this temperature for 7 h. After cooling to rt, the solution was diluted with water (20 mL) and extracted with ethyl acetate. The organic layer was washed with 5% lithium chloride solution (4×), water (2×), then brine, and dried. Removal of solvent gave an oily residue, which was purified by flash column chromatography on silica gel to give the title compounds.

**5**: prepared from **4** and 1-iodobutane as reported previously [34].

**29**: prepared from **28** and ethyl iodide. Eluent: EtOAc/PE (1:2 to 1:1). Yellow oil. Yield: 21%. %. ^1^H NMR (400 MHz, CDCl_3_): δ 7.03 (s, 1H), 5.41 (s, 1H), 4.11 (q, *J* = 6.4 Hz, 1H), 4.04 (q, *J* = 6.4 Hz, 1H), 3.99 (s, 3H), 3.30 (q, *J* = 6.3 Hz, 2H), 1.32 (t, *J* = 6.4 Hz, 3H), 1.20 (t, *J* = 6.3 Hz, 3H). MS (ESI): *m*/*z* 417 [2M+Na]^+^, 220 [M+Na]^+^, 198 [M+H]^+^.

**30**: prepared from **28** and 1-iodobutane. Yellow oil. Used in the next step without purification.

**41**: prepared from **39** and 1-iodobutane. Eluent: EtOAc/PE (1:4). Orange oil. Yield: 50%. ^1^H NMR (400 MHz, CDCl_3_): δ 3.96 (s, 3H), 3.89 (s, 3H), 3.16 (t, *J* = 6.9 Hz, 2H), 2.29 (s, 3H), 1.51 (m, 2H), 1.37 (m, 2H), 0.92 (t, *J* = 7.3 Hz, 3H). MS (ESI): *m*/*z* 248 [M+Na]^+^, 226 [M+H]^+^.

**42**: prepared from **39** and 1-iodopentane. Eluent: EtOAc/PE (1:3). Yellow oil. Yield: 78%. ^1^H NMR (400 MHz, CDCl_3_): δ 3.96 (s, 3H), 3.89 (s, 3H), 3.16 (t, *J* = 6.9 Hz, 3H), 2.29 (s, 3H), 1.51 (m, 2H), 1.37 (m, 4H), 0.92 (t, *J* = 7.3 Hz, 3H). MS (ESI): *m*/*z* 240 [M+H]^+^.

**44**: prepared from **40** and 1-iodobutane. Eluent: EtOAc/PE (1:1). Orange oil. Yield: 43%. ^1^H NMR (400 MHz, CDCl_3_): δ 3.96 (s, 3H), 3.89 (s, 3H), 3.16 (t, *J* = 6.9 Hz, 2H), 2.29 (s, 3H), 1.50 (m, 2H), 1.37 (m, 2H), 0.92 (t, *J* = 7.3 Hz, 3H). MS (ESI): *m*/*z* 226 [M+H]^+^.

**45** prepared from **40** and 1-iodopentane. Eluent: EtOAc/PE (1:3). Yellow oil. Yield: 58%. ^1^H NMR (600 MHz, CDCl_3_): δ 3.92 (s, 3H), 3.43 (t, *J* = 7.5 Hz, 3H), 3.33 (t, *J* = 7.5 Hz, 3H), 2.23 (s, 3H), 1.54 (m, 2H), 1.41–1.28 (m, 4H), 0.99 (t, *J* = 6.4 Hz, 3H). MS (ESI): *m*/*z* 240 [M+H]^+^.

#### 3.1.2. General Procedure for the Synthesis of Compounds **6**, **43**

A mixture of **4** or **40** (5 mmol), 2-iodopropane (2 mL, 20 mmol), and potassium carbonate (690 mg, 5 mmol) in dry acetonitrile was refluxed under N_2_ for 16 h. After cooling, the mixture was diluted with water and EtOAc. The organic layer was separated, washed with 5% solution of sodium thiosulfate, brine, and water, then dried and evaporated. The residue was purified by flash column chromatography on silica gel.

**6**: prepared from **4**. Eluent: EtOAc. Colorless oil. Yield: 85%. ^1^H NMR (400 MHz, CDCl_3_): δ 6.74 (s, 1H), 3.86–3.75 (m, 6H), 3.17–3.13 (m, 1H), 1.12–1.10 (m, 6H). MS (ESI): *m*/*z* 198 [M+H]^+^.

**43**: prepared from **40**. Eluent: EtOAc/PE (1:1). Yellow oil. Yield: 67%. ^1^H NMR (600 MHz, CDCl_3_): δ 3.86 (s, 3H), 3.78 (s, 3H), 3.27 (m, 1H), 2.24 (s, 3H), 1.10 (d, *J* = 6.4 Hz, 6H). MS (ESI): *m*/*z* 234 [M+Na]^+^.

#### 3.1.3. General Procedure for the Synthesis of Compounds **7**, **8**

To a solution of **4** (0.1 g, 0.6 mmol) and the appropriate aromatic aldehyde (0.7 mmol) in dry dichloromethane (4 mL) was added anhydrous sodium sulfate (200–300 mg) and the mixture was stirred at room temperature overnight. Filtration of the solid and evaporation of the solution left an oily residue, which was taken up in methanol (4 mL). After adding sodium borohydride (20 mg, 0.6 mmol), the reaction mixture was stirred at rt for 2 h. Removal of methanol gave a residue, which was dissolved in dichloromethane, and this solution was washed with water, dried, and evaporated. The oily product was purified by flash column chromatography on silica gel, eluting with EtOAc/PE (1:1).

**7**: prepared from **4** and benzaldehyde. Pinky solid. Yield: 82%. ^1^H NMR (400 MHz, CDCl_3_): δ 7.23 (m, 6H), 5.53 (s, 1H), 4.68 (s, 2H), 3.98 (s, 3H), 3.88 (s, 3H). MS (ESI): *m*/*z* 513 [2M+Na]^+^, 268 [M+Na]^+^, 246 [M+H]^+^.

**8**: prepared from **4** and 2,4-dimethoxybenzaldehyde. White needles. Yield: 83%. ^1^H NMR (400 MHz, CDCl_3_): δ 7.19 (s, 1H), 7.10 (d, *J* = 8.2 Hz, 1H), 6.78 (s, 1H), 6.47–6.28 (m, 1H), 4.08 (s, 3H), 3.83 (s, 3H), 3.76 (s, 6H), 3.72 (s, 2H). MS (ESI): *m*/*z* 328 [M+Na]^+^.

#### 3.1.4. General Procedure for the Synthesis of Compounds **9**–**12**, **31**, **32**, **46**–**50**

A solution of methyl 3-chloro-3-oxopropionate (16 mL, 15 mmol) in dry dichloromethane (30–40 mL) was added dropwise to an ice-cooled solution of amines **5**–**8**, **29**, **30**, **41–45** (10 mmol) and triethylamine (20 mL, 15 mmol) in dry dichloromethane (50 mL). After stirring at rt for 2–6 h, the solution was washed with saturated solution of sodium hydrogen carbonate, then 2 N HCl and brine. The organic layer was dried and evaporated to give an oily residue, which was purified by flash column chromatography on silica gel.

**9**: prepared from **5** as previously described [34].

**10**: prepared from **6**. Eluent: EtOAc/PE (1:2 to 1:1) to EtOAc. Yellow oil. Yield: 73%. ^1^H NMR (400 MHz, CDCl_3_): δ 6.49 (s, 1H), 4.45 (hept, *J* = 6.0 Hz, 1H), 3.90 (s, 3H), 3.83 (s, 3H), 3.75 (s, 3H), 3.14 (s, 2H), 1.44 (d, *J* = 6.0 Hz, 6H). MS (ESI): *m*/*z* 320 [M+Na]^+^.

**11**: prepared from **7**. Eluent: EtOAc/PE (2:1) to EtOAc. Colorless oil. Yield: 99%. ^1^H NMR (400 MHz, CDCl_3_): δ 7.20–7.06 (m, 5H), 6.96 (s, 1H), 3.73 (s, 9H), 3.54 (s, 2H), 3.18 (s, 2H). MS (ESI): *m*/*z* 368 [M+Na]^+^, 346 [M+H]^+^.

**12**: prepared from **8**. Eluent: EtOAc/PE (1:1). Yellow oil. Yield: 98%. ^1^H NMR (400 MHz, CDCl_3_): δ 7.19 (s, 1H), 7.14 (d, *J* = 8.3 Hz, 1H), 7.00 (s, 1H), 6.36–6.34 (m, 1H), 3.78 (s, 8H), 3.71 (s, 5H), 3.60 (s, 3H), 3.53 (s, 3H). MS (ESI): *m*/*z* 428 [M+Na]^+^.

**31**: prepared from **29**. Eluent: EtOAc/PE (1:2). Orange solid. Yield: 98%. ^1^H NMR (400 MHz), CDCl_3_): δ 3.89 (s, 3H), 3.88 (s, 3H), 3.64 (s, 3H), 3.64–3.50 (m, 2H), 3.16 (d, *J* = 2.1 Hz, 2H), 2.21 (s, 3H), 1.42 (m, 2H), 1.23 (m, 4H), 0.84 (t, *J* = 7.0 Hz, 3H). MS (ESI): *m*/*z* 320 [M+Na]^+^.

**32**: prepared from **30**. Eluent: EtOAc/PE (1:2). Yellow oil. Yield: 36%. ^1^H NMR (400 MHz), CDCl_3_): δ 6.50 (s, 1H), 4.18 (q, *J* = 6.5 Hz, 1H), 4.14 (q, *J* = 6.4 Hz, 1H), 3.91 (t, *J* = 7.3 Hz, 1H), 3.83 (s, 3H), 3.75 (s, 3H), 3.59 (t, *J* = 7.3 Hz, 1H), 3.14 (s, 2H), 1.52 (m, 2H), 1.45 (m, 2H), 1.32 (t, *J* = 6.4 Hz, 3H), 1.00 (t, *J* = 6.4 Hz, 3H). MS (ESI): *m*/*z* 378 [M+Na]^+^.

**46**: prepared from **41**. Eluent: EtOAc/PE (3:1). Dark yellow oil. Yield: 94%. ^1^H NMR (400 MHz), CDCl_3_): δ 4.10 (s, 3H), 3.89 (s, 3H), 3.66 (s, 3H), 3.57 (q, *J* = 8.0 Hz, 2H), 3.14 (d, *J* = 2.2 Hz, 2H), 2.17 (s, 3H), 1.41 (m, 2H), 1.27 (m, 2H), 0.88 (t, *J* = 7.3 Hz, 3H). MS (ESI): *m*/*z* 326 [M+H]^+^.

**47**: prepared from **42**. Eluent: EtOAc/PE (1:1). Yellow oil. Yield: 99%. ^1^H NMR (600 MHz), CDCl_3_): δ 4.11 (s, 3H), 3.87 (s, 3H), 3.65 (s, 3H), 3.55 (m, 2H), 3.14 (d, *J* = 3.5 Hz, 2H), 2.17 (s, 3H), 1.45 (m, 2H), 1.25 (m, 4H), 0.85 (t, *J* = 7.1 Hz, 3H). MS (ESI): *m*/*z* 362 [M+Na]^+^.

**48**: prepared from **43**. Eluent: EtOAc/PE (1:1). Yellow oil. Yield: 76%. ^1^H NMR (600 MHz), CDCl_3_): δ 4.90 (m, 1H), 3.89 (s, 3H), 3.88 (s, 3H), 3.66 (s, 3H), 3.17 (d, *J* = 15.3 Hz, 1H), 3.11 (d, *J* = 15.3 Hz, 1H), 2.22 (s, 3H), 1.05 (d, *J* = 6.7 Hz, 3H), 0.95 (d, *J* = 6.8 Hz, 3H). MS (ESI): *m*/*z* 334 [M+Na]^+^.

**49**: prepared from **44**. Eluent: EtOAc/PE (3:1). Dark yellow oil. Yield: 96%. ^1^H NMR (600 MHz), CDCl_3_): δ 3.89 (s, 3H), 3.88 (s, 3H), 3.64 (s, 3H), 3.64–3.50 (m, 2H), 3.16 (d, *J* = 2.1 Hz, 2H), 2.21 (s, 3H), 1.38 (m, 2H), 1.26 (m, 2H), 0.86 (t, *J* = 7.3 Hz, 3H). MS (ESI): *m*/*z* 348 [M+Na]^+^.

**50**: prepared from **45**. Eluent: EtOAc/PE (1:1). Yellow oil. Yield: 99%. ^1^H NMR (600 MHz), CDCl_3_): δ 3.89 (s, 3H), 3.88 (s, 3H), 3.64 (s, 3H), 3.64–3.50 (m, 2H), 3.16 (d, *J* = 2.1, 2H), 2.21 (s, 3H), 1.42 (m, 2H), 1.23 (m, 4H), 0.84 (t, *J* = 7.0 Hz, 3H). MS (ESI): *m*/*z* 362 [M+Na]^+^.

#### 3.1.5. General Procedure for the Synthesis of the Final Compounds **13**–**27**, **33**–**35**, **51**–**55**

A solution of the corresponding precursor **9**–**12**, **31**, **32**, **46**–**50** (0.96 mmol) in anhydrous THF (35 mL) was added dropwise to a suspension of 60% sodium hydride (77 mg, 1.92 mmol) in the same solvent (20 mL) containing dry methanol (20 µL, 0.48 mmol). The reaction mixture was stirred at 60 °C for 2 h and then evaporated to leave a solid residue, which was taken up in 5% sodium carbonate solution. The aqueous solution was extracted with diethyl ether to remove some starting material, then brought to pH 1 by addition of 6 N HCl and extracted with EtOAc (3 × 20 mL). The organic layer was dried, filtered, and evaporated to give the intermediates of the Dieckmann condensation as a solid residue insoluble in most organic solvents, which was used in the next step without purification. A mixture of the bicyclic ester (0.96 mmol), the corresponding primary amine (1.92 mmol) in THF (3 mL), and toluene (10 mL) was refluxed for 1–2 h, with further toluene added dropwise and the azeotropic mixture methanol/toluene distilled off. After completion of the reaction, the solvent was removed under reduced pressure, and the solid residue was taken up in EtOAc. The organic solution was washed successively with 2 N HCl and brine, then dried, filtered, and concentrated to give a solid residue, which was purified as indicated below to yield the final compounds **13**–**27**, **33**–**35**, **51**–**55.**

4-(1-Butyl)-*N*-(*tert*-butyl)-7-hydroxy-2-methyl-5-oxo-4,5-dihydro-2*H*-pyrazolo[4,3-*b*]pyridine-6-carboxamide (**13**)

Obtained from **9** and *tert*-butylamine after purification by flash column chromatography on silica gel (EtOAc/PE 1:1 as eluent) and subsequent recrystallization from methanol. Yield: 54%. White crystals. Mp 173–176 °C. ^1^H NMR (400 MHz, CDCl_3_): δ 17.72 (s, 1H), 10.29 (s, 1H), 7.23 (s, 1H), 4.02 (s, 3H), 3.86–3.77 (m, 2H), 1.63–1.57 (m, 2H), 1.40 (s, 9H), 1.36–1.23 (m, 2H), 0.88 (t, *J* = 7.3 Hz, 3H). ^13^C NMR (100 MHz, CDCl_3_): δ 171.5, 169.2, 162.6, 132.1, 128.9, 114.2, 96.8, 51.5, 45.1, 40.6, 29.7, 28.9, 20.3, 13.8. MS (ESI): *m*/*z* 343 [M+Na]^+^, 321 [M+H]^+^. Anal. Calcd for C_16_H_24_N_4_O_3_: C, 59.98; H, 7.55; N, 17.49. Found: C, 60.19; H, 7.61; N, 17.37.

4-(1-Butyl)-*N*-cyclopentyl-7-hydroxy-2-methyl-5-oxo-4,5-dihydro-2*H*-pyrazolo[4,3-*b*]pyridine-6-carboxamide (**14**)

Prepared by cyclization of **9** and subsequent reaction with cyclopentylamine. Purified by flash column chromatography on silica gel (EtOAc/PE 1:1 as eluent) and subsequent recrystallization from methanol. Yield: 98%. White crystals. Mp 156–159 °C. ^1^H NMR (400 MHz, CDCl_3_): δ 17.48 (s, 1H), 10.26 and 10.24 (s, 1H overall), 7.24 (s, 1H), 4.31–4.25 (m, 1H), 4.03 (s, 3H), 3.87–3.78 (m, 2H), 2.00–1.97 (m, 2H), 1.73–1.43 (m, 6H), 1.36–1.28 (m, 2H), 0.89 (t, *J* = 7.3 Hz, 3H). ^13^C NMR (100 MHz, CDCl_3_): δ 171.2, 168.7, 162.5, 131.8, 129.1, 114.4, 96.5, 50.7, 45.1, 40.6, 33.1, 29.6, 23.8, 20.3, 13.8. MS (ESI): *m*/*z* 355 [M+Na]^+^, 333 [M+H]^+^. Anal. Calcd for C_17_H_24_N_4_O_3_: C, 61.43; H, 7.28; N, 16.86. Found: C, 61.32; H, 7.35; N, 16.77.

4-(1-Butyl)-*N*-cyclooctyl-7-hydroxy-2-methyl-5-oxo-4,5-dihydro-2*H*-pyrazolo[4,3-*b*]pyridine-6-carboxamide (**15**)

Prepared from **9** and cyclooctylamine. Purified by recrystallization from methanol/water (10:1). Yield: 65%. Light yellow solid. Mp 179–181 °C. ^1^H NMR (400 MHz, CDCl_3_): δ 17.53 (s, 1H), 10.29 and 10.27 (s, 1H overall), 7.24 (s, 1H), 4.15–4.05 (m, 1H), 4.03 (s, 3H), 3.86–3.80 (m, 2H), 1.92–1.80 (m, 2H), 1.73–1.45 (m, 14H), 1.40–1.25 (m, 2H), 0.89 (t, *J* = 7.3 Hz, 3H). ^13^C NMR (100 MHz, CDCl_3_): δ 170.4, 168.8, 162.6, 131.9, 129.1, 114.3, 96.5, 49.3, 45.1, 40.7, 32.0, 29.7, 27.2, 25.4, 23.7, 20.3, 13.8. MS (ESI): *m*/*z* 397 [M+Na]^+^, 375 [M+H]^+^. Anal. Calcd for C_20_H_30_N_4_O_3_: C, 64.15; H, 8.08; N, 14.96. Found: C, 64.32; H, 7.99; N, 14.80.

4-(1-Butyl)-*N*-(4-fluorophenyl)-7-hydroxy-2-methyl-5-oxo-4,5-dihydro-2*H*-pyrazolo[4,3-*b*]pyridine-6-carboxamide (**16**)

Prepared from **9** and 4-fluoroaniline and subsequent recrystallization from methanol. Yield: 47%. White needles. Mp 190–192 °C. ^1^H NMR (400 MHz, CDCl_3_): δ 16.63 (s, 1H), 12.45 (s, 1H), 7.65–7.51 (m, 2H), 7.29 (s, 1H), 7.00–6.96 (m, 2H), 4.06 (s, 3H), 3.92–3.86 (m, 2H), 1.69–1.62 (m, 2H), 1.40–1.33 (m, 2H), 0.91 (t, *J* = 7.3 Hz, 3H). ^13^C NMR (100 MHz, CDCl_3_): δ 169.9, 168.5, 162.5, 160.9, 158.5, 133.3, 131.4–129.2 (d, C-F), 122.9, 122.9, 115.7, 155.5, 114.6, 96.9, 45.4, 40.8, 29.7, 20.3, 13.8. MS (ESI): *m*/*z* 381 [M+Na]^+^, 359 [M+H]^+^. Anal. Calcd for C_18_H_19_FN_4_O_3_: C, 60.33; H, 5.34; N, 15.63. Found: C, 60.48; H, 5.25; N, 15.80.

- 4-(1-Butyl)-*N*-(4-fluorobenzyl)-7-hydroxy-2-methyl-5-oxo-4,5-dihydro-2*H*-pyrazolo[4,3-*b*]pyridine-6-carboxamide (**17**)

Prepared from **9** and 4-fluorobenzylamine and subsequent recrystallization from methanol. Yield: 54%. Yellow crystals. Mp 178–180 °C. ^1^H NMR (400 MHz, CDCl_3_): δ 17.17 (s, 1H), 10.85 (s, 1H), 7.84 (s, 1H), 7.40 (dd, *J* = 7.9, 5.6 Hz, 2H), 7.10–7.01 (m, 2H), 4.56 (d, *J* = 5.9 Hz, 2H), 4.03 (s, 3H), 3.87 (t, *J* = 7.4 Hz, 2H), 1.63–1.53 (m, 2H), 1.34–1.21 (m, 2H), 0.84 (t, *J* = 7.3 Hz, 3H). ^13^C NMR (100 MHz, acetone-d_6_): δ 172.1, 168,7, 163.3, 162.1, 160.9, 134.8, 130.8, 129.8, 129.7, 129.8, 115.7, 115.3, 115.1, 95.9, 44.5, 41.6, 40.0, 19.8, 13.2. MS (ESI): *m*/*z* 395 [M+Na]^+^, 373 [M+H]^+^. Anal. Calcd for C_19_H_21_FN_4_O_3_: C, 61.28; H, 5.68; N, 15.05. Found: C, 61.48; H, 5.59; N, 14.91.

4-(1-Butyl)-7-hydroxy-2-methyl-5-oxo-*N*-(1,2,3,4-tetrahydronaphthalen-1-yl)-4,5-dihydro-2*H*-pyrazolo[4,3-*b*]pyridine-6-carboxamide (**18**)

Prepared from **9** and 1,2,3,4-tetrahydro-1-naphthylamine followed by recrystallization from methanol/water (10:1). Yield: 60%. Light orange crystals. Mp 180–182 °C. ^1^H NMR (400 MHz, CDCl_3_): δ 17.36 (s, 1H), 10.58 e 10.56 (s, 1H overall), 7.31–7.27 (m, 2H), 7.24 (s, 1H), 7.11–7.06 (m, 2H), 5.30 (m, 1H), 4.04 (s, 3H), 3.90–3.69 (m, 2H), 2.86–2.64 (m, 2H), 2.14–2.01 (m, 2H), 1.95–1.76 (m, 2H), 1.65–1.52 (m, 2H), 1.35–1.25 (m, 2H), 0.87 (t, *J* = 7.3 Hz, 3H). ^13^C NMR (100 MHz, CDCl_3_): δ 171.0, 168.6, 162.4, 137.4, 136.4, 131.7, 129.2, 129.1, 128.8, 127.2, 126.3, 114.4, 96.7, 47.1, 45.1, 40.7, 30.1, 29.6, 20.3, 20.2, 13.8. MS (ESI): *m*/*z* 417 [M+Na]^+^, 395 [M+H]^+^. Anal. Calcd for C_22_H_26_N_4_O_3_: C, 66.99; H, 6.64; N, 14.20. Found: C, 67.18; H, 6.58; N, 14.06.

*N*-Benzhydryl-4-(1-butyl)-7-hydroxy-2-methyl-5-oxo-4,5-dihydro-2*H*-pyrazolo[4,3-*b*]pyridine-6-carboxamide (**19**)

Prepared from **9** and benzhydrylamine. Purified by trituration with PE. Yield: 74%. White flakes. Mp 173–176 °C. ^1^H NMR (400 MHz, CDCl_3_): δ 16.89 (s, 1H), 11.17 (s, 1H), 7.37–7.12 (m, 11H), 6.35 (d, *J* = 8.2 Hz, 1H), 4.03 (s, 3H), 3.88–3.79 (m, 2H), 1.67–1.59 (m, 2H), 1.38–1.30 (m, 2H), 0.90 (t, *J* = 7.3 Hz, 3H). ^13^C NMR (100 MHz, CDCl_3_): δ 170.9, 168.4, 162.5, 141.6, 131.5, 129.2, 128.8, 127.4, 127.4, 127.2, 114.5, 96.8, 56.8, 45.2, 40.7, 29.7, 20.3, 13.8. MS (ESI): *m*/*z* 453 [M+Na]^+^, 431 [M+H]^+^. Anal. Calcd for C_25_H_26_N_4_O_3_: C, 69.75; H, 6.09; N, 13.01. Found: C, 69.90; H, 6.16; N, 12.88.

*tert*-Butyl (4-Butyl-7-hydroxy-2-methyl-5-oxo-4,5-dihydro-2*H*-pyrazolo[4,3-*b*]pyridine-6-carbonyl)-*L*-phenylalaninate (**20**)

Prepared from **9** and L-phenylalanine *tert*-butyl ester hydrochloride in the presence of DBU. Purified by flash column chromatography on silica gel (EtOAc/PE 1:2 to 1:1 as eluent). Yield: 34%. Yellow amorphous solid. ^1^H NMR (400 MHz, CDCl_3_): δ 16.69 (s 1H), 10.80 e 10.78 (s, 1H overall), 7.26–7.11 (m, 6H), 4.75 (q, *J* = 6.9 Hz, 1H), 4.01 (s, 3H), 3.90–3.75 (m, 2H), 3.19–3.02 (m, 2H), 1.67–1.53 (m, 2H), 1.32 (s, 9H), 1.18 (t, *J* = 7.7 Hz, 2H), 0.88 (t, *J* = 7.3 Hz, 3H). ^13^C NMR (100 MHz, CDCl_3_): δ 171.3, 170.2, 168.1, 162.3, 136.6, 131.4, 129.5, 129.3, 128.5, 128.3, 126.8, 114.4, 96.8, 82.1, 60.4, 54.2, 45.0, 40.7, 38.5, 29.6, 27.9, 20.2, 14.2, 13.8. MS (ESI): *m*/*z* 491 [M+Na]^+^, 469 [M+H]^+^. Anal. Calcd for C_25_H_32_N_4_O_5_: C, 64.09; H, 6.88; N, 11.96. Found: C, 63.90; H, 6.79; N, 12.18.

*N*-(Adamantan-1-yl)-7-hydroxy-4-isopropyl-2-methyl-5-oxo-4,5-dihydro-2*H*-pyrazolo[4,3-*b*]pyridine-6-carboxamide (**21**)

Obtained from **10** and 1-aminoadamantane after recrystallization from methanol. Yield: 87%. White solid. Mp 267–269 °C. ^1^H NMR (400 MHz, CDCl_3_): δ 17.68 (s, 1H), 10.30 (s, 1H), 7.37 (s, 1H), 5.32 (s, 1H), 4.03 (s, 3H), 2.08–2.05 (m, 9H), 1.66–1.67 (m, 7H), 1.40–1.35 (m, 5H). ^13^C NMR (100 MHz, CDCl_3_): δ 171.3, 168.8, 162.8, 132.9, 125.5, 114.9, 96.8, 52.4, 41.5, 40.6, 36.4, 29.5, 19.3. MS (ESI): *m*/*z* 407 [M+Na]^+^, 385 [M+H]^+^. Anal. Calcd for C_21_H_28_N_4_O_3_: C, 65.60; H, 7.34; N, 14.57. Found: C, 65.82; H, 7.43; N, 14.38.

7-Hydroxy-*N*-(1-hydroxyadamantan-3-yl)-4-isopropyl-2-methyl-5-oxo-4,5-dihydro-2*H*-pyrazolo[4,3-*b*]pyridine-6-carboxamide (**22**)

Obtained from **10** and 3-hydroxy-1-adamantanamine followed by recrystallization from methanol. Yield: 69%. White needles. Mp > 270 °C. ^1^H NMR (400 MHz, CDCl_3_): δ 17.45 (s, 1H), 10.40 (s, 1H), 7.38 (s, 1H), 5.30 (s, 1H), 3.99 (s, 3H), 2.24–1.95 (m, 5H), 1.69–1.41 (m, 15H). ^13^C NMR (100 MHz, CDCl_3_): δ 171.4, 168.6, 162.7, 132.7, 155.0, 96.8, 69.2, 54.6, 49.0, 44.2, 40.6, 40.2, 34.9, 30.7, 19.3. MS (ESI): *m*/*z* 423 [M+Na]^+^, 401 [M+H]^+^. Anal. Calcd for C_21_H_28_N_4_O_4_: C, 62.98; H, 7.05; N, 13.99. Found: C, 63.17; H, 7.13; N, 13.87.

*N*-Cyclooctyl-7-hydroxy-4-isopropyl-2-methyl-5-oxo-4,5-dihydro-2*H*-pyrazolo[4,3-*b*]pyridine-6-carboxamide (**23**)

Prepared from **10** and cyclooctylamine. Purified by recrystallization from methanol. Yield: 91%. Yellow solid. Mp 188–191 °C. ^1^H NMR (400 MHz, CDCl_3_): δ 17.47 (s, 1H), 10.35 and 10.33 (s, 1H overall), 7.38 (s, 1H), 5.30 (s, 1H), 4.07–3.98 (m, 4H), 1.64–1.50 (m, 14H), 1.39–1.33 (m, 6H). ^13^C NMR (100 MHz, CDCl_3_): δ 170.6, 168.7, 162.8, 132.9, 125.8, 115.2, 96.6, 49.2, 45.4, 40.6, 31.9, 27.3, 25.4, 23.6, 19.3. MS (ESI): *m*/*z* 383 [M+Na]^+^, 361 [M+H]^+^. Anal. Calcd for C_19_H_28_N_4_O_3_: C, 63.31; H, 7.83; N, 15.54. Found: C, 63.18; H, 7.74; N, 15.37.

7-Hydroxy-4-isopropyl-2-methyl-5-oxo-*N*-(1,2,3,4-tetrahydronaphthalen-1-yl)-4,5-dihydro-2*H*-pyrazolo[4,3-*b*]pyridine-6-carboxamide (**24**)

Obtained from **10** and 1,2,3,4-tetrahydro-1-naphthylamine. Purified by recrystallization from methanol. Yield: 96%. Yellow-ocher solid. Mp 214–216 °C. ^1^H NMR (400 MHz, CDCl_3_): δ 17.32 (s, 1H), 10.65 and 10.63 (s, 1H overall), 7.39 (s, 1H), 7.31–7.29 (m, 2H), 7.10–7.06 (m, 2H), 5.33–5.28 (m, 1H), 4.05 (s, 3H), 2.84–2.68 (m, 2H), 2.10–2.04 (m, 2H), 1.93–1.81 (m, 2H), 1.34 (d, *J* = 7.0 Hz, 6H). ^13^C NMR (100 MHz, CDCl_3_): δ 170.9, 168.2, 162.5, 137.3, 136.5, 132.5, 129.1, 128.8, 127.2, 126.3, 125.8, 115.1, 96.7, 47.1, 45.3, 40.7, 30.1, 29.2, 20.1, 19.3. MS (ESI): *m*/*z* 403 [M+Na]^+^, 381[M+H]^+^. Anal. Calcd for C_21_H_24_N_4_O_3_: C, 66.30; H, 6.36; N, 14.73. Found: C, 66.51; H, 6.28; N, 14.60.

*N*-Benzhydryl-7-hydroxy-4-isopropyl-2-methyl-5-oxo-4,5-dihydro-2*H*-pyrazolo[4,3-*b*]pyridine-6-carboxamide (**25**)

Prepared from **10** and benzhydrylamine. Purified by trituration with PE. Yield: 71%. White solid. Mp 205–208 °C. ^1^H NMR (400 MHz, CDCl_3_): δ 16.86 (s, 1H), 11.26 and 11.24 (s, 1H overall), 7.41 (s, 1H), 7.31–7.18 (m, 10H), 6.34–6.32 (m, 1H), 5.36 (s, 1H), 4.03 (s, 3H), 1.37 (d, *J* = 7.0, 6H). ^13^C NMR (100 MHz, CDCl_3_): δ 170.9, 167.8, 162.6, 155.9, 141.6, 132.3, 128.8, 127.4, 127.3, 127.2, 115.2, 103.7, 96.8, 56.9, 40.7, 19.4. MS (ESI): *m*/*z* 439 [M+Na]^+^, 417 [M+H]^+^. Anal. Calcd for C_24_H_24_N_4_O_3_: C, 69.21; H, 5.81; N, 13.45. Found: C, 69.41; H, 5.88; N, 13.26.

*N*-(Adamantan-1-yl)-4-benzyl-7-hydroxy-2-methyl-5-oxo-4,5-dihydro-2*H*-pyrazolo[4,3-*b*]pyridine-6-carboxamide (**26**)

Prepared from **11** and 1-aminoadamantane followed by recrystallization from methanol. Yield: 68%. White solid. Mp 180–183 °C. ^1^H NMR (400 MHz, CDCl_3_): δ 17.90 (s, 1H), 10.21 (s, 1H), 7.29–7.13 (m, 5H), 7.04 (s, 1H), 5.09 (s, 2H), 3.91 (s, 3H), 2.09–2.05 (m, 7H), 1.68–1.61 (m, 5H), 1.20–1.16 (m, 3H). ^13^C NMR (100 MHz, CDCl_3_): δ 171.4, 169.9, 163.0, 135.8, 132.2, 128.9, 128.6, 127.7, 126.9, 114.8, 52.6, 47.7, 41.6, 40.6, 36.4, 29.5. MS (ESI): *m*/*z* 455 [M+Na]^+^, 433 [M+H]^+^. Anal. Calcd for C_25_H_28_N_4_O_3_: C, 69.42; H, 6.53; N, 12.95. Found: C, 69.61; H, 6.44; N, 12.76.

*N*-(Adamantan-1-yl)-4-(2,4-dimethoxybenzyl)-7-hydroxy-2-methyl-5-oxo-4,5-dihydro-2*H*-pyrazolo[4,3-*b*]pyridine-6-carboxamide (**27**)

Prepared from **11** and 1-aminoadamantane followed by recrystallization from methanol/dichloromethane (10:1). Yield: 37%. Glossy white solid. Mp >270 °C. ^1^H NMR (400 MHz, CDCl_3_): δ 17.85 (s, 1H), 10.31 (s, 1H), 7.18 (s, 1H), 6.90 (d, *J* = 8.4 Hz, 1H), 6.44 (s, 1H), 6.36 (dd, *J* = 8.4, 2.2 Hz, 1H), 5.07 (s, 2H), 3.98 (s, 3H), 3.85 (s, 3H) 3.74 (s, 3H), 2.13 (m, &H), 2.09 (m, 3H), 1.69 (m, 6H). ^13^C NMR (100 MHz, CDCl_3_): δ 171.4, 169.5, 163.1, 160.3, 157.9, 132.0, 129.0, 128.7, 116.2, 115.2, 104.5, 98.5, 96.7, 55.5, 55.3, 52.4, 42.0, 41.5, 40.6, 36.4, 29.5. MS (ESI): m/z 515 [M+Na]^+^, 493 [M+H]^+^. Anal. Calcd for C_27_H_32_N_4_O_5_: C, 65.84; H, 6.55; N, 11.37. Found: C, 65.61; H, 6.47; N, 11.16.

*N*-(Adamantan-1-yl)-2,4-diethyl-7-hydroxy-5-oxo-4,5-dihydro-2*H*-pyrazolo[4,3-*b*]pyridine-6-carboxamide (**33**)

Obtained from **31** and 1-adamantanamine followed by recrystallization from methanol. Yield: 83%. White needles. Mp 245–247 °C. ^1^H NMR (400 MHz, CDCl_3_): δ 17.71 (s, 1H), 10.24 (s, 1H), 7.28 (s, 1H), 4.31–4.26 (m, 2H), 3.93–3.88 (m, 2H), 2.09–2.05 (m, 8H), 1.69–1.50 (m, 10H), 1.25–1.19 (m, 3H). ^13^C NMR (100 MHz, CDCl_3_): δ 171.3, 169.4, 162.5, 131.9, 128.2, 112.4, 96.7, 52.4, 48.9, 41.5, 39.9, 36.4, 36.3, 29.5, 15.5, 12.7. MS (ESI): *m*/*z* 407 [M+Na]^+^. Anal. Calcd for C_21_H_28_N_4_O_3_: C, 65.60; H, 7.34; N, 14.57. Found: C, 65.42; H, 7.25; N, 14.40.

4-(1-Butyl)-*N*-(tert-butyl)-2-ethyl-7-hydroxy-5-oxo-4,5-dihydro-2*H*-pyrazolo[4,3-*b*]pyridine-6-carboxamide (**34**)

Prepared from **32** and *tert*-butylamine. Purified by flash column chromatography on silica gel (EtOAc/PE 1:2) and recrystallization from methanol. Yield: 61%. White crystals. Mp 151–153 °C. ^1^H NMR (400 MHz, CDCl_3_): δ 17.75 (s, 1H), 10.30 (s, 1H), 7.25 (s, 1H), 4.31–4.26 (m, 2H), 3.85–3.81 (m, 2H), 1.66–1.60 (m, 2H), 1.54–1.51 (m, 3H), 1.41 (s, 9H), 1.37–1.31 (m, 2H), 0.90 (t, *J* = 7.3 Hz, 3H). ^13^C NMR (100 MHz, CDCl_3_): δ 171.5, 169.3, 162.6, 131.8, 128.7, 112.7, 96.7, 51.5, 48.9, 45.1, 29.7, 28.9, 20.3, 15.5, 13.8. MS (ESI): *m*/*z* 357 [M+Na]^+^, 335 [M+H]^+^. Anal. Calcd for C_17_H_26_N_4_O_3_: C, 61.06; H, 7.84; N, 16.75. Found: C, 60.81; H, 7.77; N, 16.86.

*N*-(Adamantan-1-yl)-4-(1-butyl)-2-ethyl-7-hydroxy-5-oxo-4,5-dihydro-2*H*-pyrazolo[4,3-*b*]pyridine-6-carboxamide (**35**)

Prepared from **32** and 1-adamantanamine. Purified by flash column chromatography on silica gel (EtOAc/PE 1:2) and recrystallization from methanol. Yield: 81%. Pale brown solid. Mp 152–154 °C. ^1^H NMR (400 MHz, CDCl_3_): δ 17.77 (s, 1H), 10.28 (br s, 1H), 7.29 (s, 1H), 4.33 (q, *J* = 7.3 Hz, 2H), 3.87 (t, *J* = 7.7 Hz, 2H), 2.13 (m, 6H), 2.09 (m, 3H), 1.68 (m, 6H), 1.65 (m, 2H), 1.56 (t, *J* = 7.3 Hz, 3H), 1.36 (m, 2H), 0.94 (t, *J* = 7.3 Hz, 3H). ^13^C NMR (100 MHz, CDCl_3_): δ 171.3, 169.3, 162.6, 131.9, 128.6, 112.6, 96.7, 52.4, 49.0, 45.1, 41.5, 36.4, 29.7, 29.4, 20.3, 15.6, 13.8. MS (ESI): *m*/*z* 411 [M−H]^−^, 413 [M+H]^+^. Anal. Calcd for C_23_H_32_N_4_O_3_: C, 66.96; H, 7.82; N, 13.58. Found: C, 60.81; H, 7.77; N, 16.86.

*N*-(Adamantan-1-yl)-4-(1-butyl)-7-hydroxy-1,3-dimethyl-5-oxo-4,5-dihydro-1*H*-pyrazolo[4,3-*b*]pyridine-6-carboxamide (**51**)

Obtained from **46** and 1-adamantanamine followed by flash column chromatography on silica gel (EtOAc/PE 1:1) and recrystallization from methanol. Yield: 45%. White solid. Mp 162–165 °C. ^1^H NMR (400 MHz, CDCl_3_): δ 18.82 and 18.08 (s, 1H overall), 11.04 and 10.47 (s, 1H overall), 4.20 and 4.12 (s, 3H overall), 4.10–3.97 (m, 2H), 2.51 (s, 3H), 2.12 (m, 6H), 2.09 (m, 3H), 1.76–1.58 (m, 8H), 1.36 (m, 4H), 0.89 (t, *J* = 6.9 Hz, 3H). ^13^C NMR (100 MHz, CDCl_3_): δ 171.6, 171.4, 169.3, 166.0, 165.4, 162.1, 131.5, 131.2, 128.1, 127.0, 124.3, 122.6, 95.5, 92.4, 53.0, 52.5, 43.2, 43.0, 41.5, 41.4, 38.7, 38.5, 36.3, 36.2, 31.6, 29.4, 19.9, 19.8, 14.1, 14.0, 13.8, 13.7. MS (ESI) *m*/*z* 413 [M+H]^+^. Anal. Calcd for C_23_H_32_N_4_O_3_: C, 66.96; H, 7.82; N, 13.58. Found: C, 67.19; H, 7.89; N, 13.37.

*N*-(Adamantan-1-yl)-7-hydroxy-1,3-dimethyl-5-oxo-4-(1-pentyl)-4,5-dihydro-1*H*-pyrazolo[4,3-*b*]pyridine-6-carboxamide (**52**)

Prepared from **47** and 1-adamantanamine followed by flash column chromatography on silica gel (EtOAc/PE 4:1) and recrystallization from methanol. Yield: 47%. White solid. Mp 167–169 °C. ^1^H NMR (400 MHz, CDCl_3_): δ 18.82 and 18.08 (s, 1H overall), 11.04 and 10.47 (s, 1H overall), 4.20 and 4.12 (s, 1H overall), 4.10–3.97 (m, 2H), 2.51 (s, 3H), 2.12 (m, 6H), 2.09 (m, 3H), 1.76–1.58 (m, 8H), 1.38–1.30 (m, 4H), 0.89 (t, *J* = 6.9 Hz, 3H). ^13^C NMR (100 MHz, CDCl_3_): δ 171.6, 171.5, 169.4, 166.0, 165.5, 162.2, 131.5, 131.3, 128.1, 127.1, 124.4, 122.7, 95.6, 94.4, 53.0, 52.5, 43.4, 43.2, 41.6, 41.4, 38.8, 38.5, 36.3, 29.4, 29.3, 28.8, 28.6, 22.5, 22.4, 14.1, 14.0, 13.9. MS (ESI) *m*/*z* 427 [M+H]^+^. Anal. Calcd for C_24_H_34_N_4_O_3_: C, 67.58; H, 8.03; N, 13.13. Found: C, 67.29; H, 7.85; N, 12.95.

*N*-(Adamantan-1-yl)-7-hydroxy-4-isopropyl-2,3-dimethyl-5-oxo-4,5-dihydro-2*H*-pyrazolo[4,3-*b*]pyridine-6-carboxamide (**53**)

Prepared from **48** and 1-adamantanamine followed by flash column chromatography on silica gel (EtOAc/PE 1:1) and recrystallization from methanol. Yield: 5%. White solid. Mp >260 °C. ^1^H NMR (400 MHz, CDCl_3_): δ 17.45 (s, 1H), 10.31 (s, 1H), 3.98 (s, 3H), 3.72 (d, *J* = 6.6 Hz, 1H), 2.55 (s, 3H), 2.14 (m, 6H), 2.09 (m, 3H), 1.75–1.63 (m, 6H), 1.59 (d, *J* = 6.9 Hz, 6H). ^13^C NMR (100 MHz, CDCl_3_): 171.2, 168.5, 164.0, 131.6, 122.2, 121.3, 96.7, 52.2, 41.5, 38.6, 36.4, 29.7, 29.4, 20.2, 12.4. MS (ESI) *m*/*z* 339 [M+H]^+^. Anal. Calcd for C_22_H_30_N_4_O_3_: C, 66.31; H, 7.59; N, 14.06. Found: C, 66.06; H, 7.47; N, 14.25.

*N*-(Adamantan-1-yl)-4-(1-butyl)-7-hydroxy-2,3-dimethyl-5-oxo-4,5-dihydro-2*H*-pyrazolo[4,3-*b*]pyridine-6-carboxamide (**54**)

Prepared from **49** and 1-adamantanamine followed by flash column chromatography on silica gel (EtOAc/PE 1:1) and recrystallization from methanol. Yield: 20%. White solid. Mp 213–216 °C. ^1^H NMR (400 MHz, CDCl_3_): δ 17.49 (s, 1H), 10.29 (s, 1H), 3.99 (t, *J* = 8.0 Hz, 2H), 3.94 (s, 3H), 2.50 (s, 3H), 2.11 (m, 6H), 2.11–2.03 (m, 3H), 1.75–1.52 (m, 8H), 1.49–1.38 (m, 2H), 0.90 (t, *J* = 7.3 Hz, 3H). ^13^C NMR (100 MHz, CDCl_3_): 171.2, 168.6, 162.8, 131.3, 124.7, 121.6, 96.5, 52.3, 42.5, 41.5, 38.4, 36.4, 31.2, 29.4, 19.9, 13.9, 11.1. MS (ESI) *m*/*z* 435 [M+Na]^+^. Anal. Calcd for C_23_H_32_N_4_O_3_: C, 66.96; H, 7.82; N, 13.58. Found: C, 67.17; H, 7.74; N, 13.40.

*N*-(Adamantan-1-yl)-7-hydroxy-2,3-dimethyl-5-oxo-4-(1-pentyl)-4,5-dihydro-2*H*-pyrazolo[4,3-*b*]pyridine-6-carboxamide (**55**)

Prepared from **50** and 1-adamantanamine followed by flash column chromatography on silica gel (EtOAc/PE 1:1) and recrystallization from methanol. Yield: 10%. White solid. Mp 67 °C. ^1^H NMR (400 MHz, CDCl_3_): δ 17.52 (s, 1H), 10.30 (s, 1H), 4.01 (t, *J* = 8.0 Hz, 2H), 3.95 (s, 3H), 2.53 (s, 3H), 2.13 (m, 6H), 2.09 (m, 3H), 1.76–1.57 (m, 8H), 1.36 (m, 4H), 0.90 (t, *J* = 7.0 Hz, 3H). ^13^C NMR (100 MHz, CDCl_3_): 171.2, 168.7, 162.8, 131.4, 124.8, 121.6, 96.5, 52.3, 42.8, 41.5, 38.4, 36.4, 29.4, 28.9, 28.7, 22.5, 14.0, 11.2. MS (ESI) *m*/*z* 427 [M+H]^+^. Anal. Calcd for C_24_H_34_N_4_O_3_: C, 67.58; H, 8.03; N, 13.13. Found: C, 67.80; H, 8.12; N, 12.89.

#### 3.1.6. General Procedure for the Synthesis of Compounds **37** and **38**

A mixture of methyl 3-methyl-4-nitro-2*H*-pyrazole-5-carboxylate (**36**) (0.400 g, 2.16 mmol), potassium carbonate (0.328 g, 2.38 mmol), and dimethyl sulfate (0.2 mL, 2.38 mmol) in acetone (30 mL) was stirred at room temperature overnight, then the solvent was removed under reduced pressure. The yellow solid residue was diluted with water (6 mL) and extracted with EtOAc. The organic layer was washed with brine, dried, filtered, and evaporated to give an orange oily residue, which was purified by flash column chromatography on silica gel.

Methyl 1,3-Dimethyl-4-nitro-1*H*-pyrazole-5-carboxylate (**37**). Elution with EtOAc:PE (1:3) gave the first regioisomer as a white solid. Yield 50%. Mp 77–79 °C. ^1^H NMR (400 MHz, CDCl3): δ 3.98 (s, 3H), 3.93 (s, 3H), 2.47 (s, 3H). MS (ESI): *m*/*z* 222 [M+Na]^+^.

Methyl 1,5-Dimethyl-4-nitro-1*H*-pyrazole-3-carboxylate (**38**). Further elution with EtOAc furnished the second isomer **41** as a dark yellow solid. Yield 50%. Mp: 62–66 °C. ^1^H NMR (400 MHz, CDCl3): δ 3.96 (s, 3H), 3.88 (s, 3H), 2.60 (s, 3H). MS (ESI): *m*/*z* 222 [M+Na]^+^.

#### 3.1.7. General Procedure for the Synthesis of Compounds **39** and **40**

Ammonium formate (1.2 g, 19 mmol) and 10% Pd/C (0.024 g, 0.228 mmol) were added under a nitrogen atmosphere to a solution of **37** or **38** (0.379 g, 1.90 mmol) in EtOH (10 mL) and H_2_O (1 mL). The reaction mixture was stirred at 50 °C for 2–3 h. After cooling to room temperature, the mixture was filtered through a pad of celite and the filtrate was diluted with H_2_O. The aqueous phase was extracted with EtOAc and the organic layer was dried, filtered and the solvent was removed under reduced pressure to give a residue that was sufficiently pure to be used directly in the next reaction.

Methyl 4-Amino-1,3-dimethyl-1*H*-pyrazole-5-carboxylate (**39**). Prepared from **37**. Orange solid. Yield 99%. Mp: 82–85 °C. ^1^H NMR (400 MHz, CDCl_3_): δ 3.98 (s, 3H), 3.90 (s, 3H), 2.15 (s, 3H). MS (ESI): *m*/*z* 170 [M+H]^+^.

Methyl 4-Amino-1,5-dimethyl-1*H*-pyrazole-3-carboxylate (**40**). Prepared from **38**. White solid. Yield 99%. Mp: 75–78 °C. ^1^H NMR (400 MHz, CDCl3): δ 3.91 (s, 3H), 3.79 (s, 3H), 2.18 (s, 3H). MS (ESI): *m*/*z* 169 [M+Na]^+^.

### 3.2. Preparation of Salts of Compounds ***63***

#### 3.2.1. *N*-(Adamantan-1-yl)-4-(1-butyl)-7-hydroxy-2-methyl-5-oxo-4,5-dihydro-2H-pyrazolo[4,3-*b*]pyridine-6-carboxamide Sodium Salt

The mother solution of NaOH was prepared by dissolving NaOH (40 g) in distilled water (80 mL). An aliquot of 20 mL of this solution (about 10 M) was then diluted in 250 mL of water to give a solution of about 0.8 M (dilution factor: 12.5), which was titrated with a 1 N solution of oxalic acid dihydrate. Four titrations were performed, yielding the following concentrations of NaOH: 0.9562 M, 0.9580 M, 0.9627 M, and 0.9530 M, with arithmetic mean: 0.9557 M. Considering the dilution factor, the exact molarity of the NaOH mother solution was found to be 11.947 M. To a suspension of **63** (300 mg, 0.754 mmol), previously crystallized from MeOH, in EtOH (99.5% *v*/*v*, 2.02 mL) was added the 11.947 M NaOH solution (63 µL, 0.753 mmol). The suspension was kept at room temperature for 2 h with stirring. The EtOH/water azeotrope was removed under vacuum to give **63-sodium salt** as a white solid. Yield: 100%. Mp > 300 °C. ^1^H NMR (400 MHz, CD_3_OD): δ 7.49 (s, 1H), 3.93 (s, 3H), 3.79 (t, 2H), 2.12–2.00 (m, 10H), 1.70–1.64 (m, 7H), 1.63–1.56 (m, 2H), 1.38–1.28 (m, 2H), 0.87 (t, *J* = 7.3 Hz, 3H).

#### 3.2.2. *N*-(Adamantan-1-yl)-4-(1-butyl)-7-hydroxy-2-methyl-5-oxo-4,5-dihydro-2H-pyrazolo[4,3-*b*]pyridine-6-carboxamide Potassium Salt

The mother solution of KOH was prepared by dissolving KOH (56 g) in distilled water (80 mL). An aliquot of 20 mL of this solution (about 10 M) was then diluted in 250 mL of water to give a solution of about 0.8 M (dilution factor: 12.5), which was titrated with a 1 N solution of oxalic acid dihydrate. Four titrations were performed, yielding the following concentrations of KOH: 0.6143 M, 0.6141 M, 0.6117 M, and 0.6151 M, with arithmetic mean: 0.6145 M. Considering the dilution factor, the exact molarity of the NaOH mother solution was found to be 7.681 M. To a suspension of **63** (200 mg, 0.503 mmol), previously crystallized from MeOH, in EtOH (99.5% *v*/*v*, 1.35 mL) was added the 7.681 M KOH solution (65 µL, 0.500 mmol). The resulting solution was kept at room temperature for 2 h with stirring. The EtOH/water azeotrope was removed under vacuum to give **63**-potassium salt as a white solid. Yield: 100%. Mp >300 °C. ^1^H NMR (400 MHz, CD_3_OD): δ 7.48 (s, 1H), 3.92 (s, 3H), 3.77 (t, 2H), 2.10–1.99 (m, 10H), 1.69–1.64 (m, 7H), 1.61–1.54 (m, 2H), 1.34–1.28 (m, 2H), 0.87 (t, *J* = 7.3 Hz, 3H).

#### 3.2.3. *N*-(Adamantan-1-yl)-4-(1-butyl)-7-hydroxy-2-methyl-5-oxo-4,5-dihydro-2H-pyrazolo[4,3-*b*]pyridine-6-carboxamide Cesium Salt

To a suspension of **63** (120 mg, 0.302 mmol), previously crystallized from MeOH, in EtOH (99.5% *v*/*v*, 1.3 mL) was added a commercial solution of CsOH (50% *w*/*v*, 90 µL, 0.300 mmol). The resulting solution was stirred at room temperature for 15 min, then the EtOH/water azeotrope was removed under vacuum to give **63**-cesium salt as a white solid. Yield: 100%. Mp > 300 °C. ^1^H NMR (400 MHz, CD_3_OD): δ 7.47 (s, 1H), 3.92 (s, 3H), 3.77 (t, 2H), 2.11–1.99 (m, 10H), 1.82–1.68 (m, 7H), 1.64–1.56 (m, 2H), 1.35–1.29 (m, 2H), 0.88 (t, *J* = 7.3 Hz, 3H).

#### 3.2.4. *N*-(Adamantan-1-yl)-4-(1-butyl)-7-hydroxy-2-methyl-5-oxo-4,5-dihydro-2H-pyrazolo[4,3-*b*]pyridine-6-carboxamide Tetramethylammonium Salt

To a suspension of **63** (120 mg, 0.302 mmol), previously crystallized from MeOH, in EtOH (99.5% *v*/*v*, 1.3 mL) was added a commercial solution of tetramethylammonium hydroxide (25% *w*/*v*, 0.11 mL, 0.302 mmol). The resulting solution was stirred at room temperature for 15 min, then the EtOH/water azeotrope was removed under vacuum to give **63**-tetramethylammonium salt as a colorless and glassy solid. Yield: 100%. Mp > 300 °C. ^1^H NMR (400 MHz, CDCl_3_): δ 7.26 (s, 1H), 4.08 (s, 3H), 3.91 (t, 2H), 3.40 (s, 6H), 3.22 (s, 6H), 2.11–2.04 (m, 10H), 1.50–1.38 (m, 7H), 1.36–1.28 (m, 2H), 1.21–1.15 (m, 2H), 0.87 (t, *J* = 7.3 Hz, 3H).

### 3.3. Dissolution of ***63***-Salts in 0.9% NaCl Solution

An excess amount (~4 to 5 mg) of each salt of compound **63** was added to 5 mL of a 0.9% NaCl solution, kept under constant agitation, and thermostated at room temperature (21 ± 2 °C). At fixed time points, an aliquot was withdrawn from the solution, and centrifuged (3000 g, 10 min, RT) to separate undissolved compound and the supernatant, after dilution with MeOH, was analyzed by LC-MS/MS. An Accela UHPLC system coupled with a TSQ Quantum Access Max triple quad mass spectrometer (Thermo Fisher, Waltham, MA, USA), was employed to quantify dissolved **63** over time. UHPLC separation occurred by gradient elution, employing a Synergy Fusion C18 80Å RP-column (2.0 × 100 mm, 4 µm; Phenomenex, Bologna, Italy). Mobile phases A and B were acetonitrile and water, both with 0.1% *v/v* formic acid added. The following elution conditions were employed: linear gradient from 60 to 95%A in 5 min; 95% A between 5 and 8 min, returning to 60% A in 0.5 min with 2.5 min reconditioning time. Total run time: 11 min. The flow rate was 0.35 mL/min and injection volume was 10 µL. Heated electrospray (H-ESI) ion source voltage was set at 4.0 KV; capillary temperature at 270 °C. Nitrogen served both as sheath and auxiliary gas at 35 psi and 15 psi, respectively. Argon at a pressure of 1.5 mtorr was employed as collision gas. H-ESI ion source operated in positive ion mode (ESI+) and acquisition occurred in multiple reaction monitoring (MRM). Tube lens voltages (TL) and collision energies (CE) for each parent-product ion transition were optimized by flow injection analysis of 5 µM solutions of each standard in MeOH. Compound **63**: *m*/*z* = 399.2 [M+H]^+^ → *m*/*z* = 135.1, 107.1, 93.1 (TL = 98 V; CE = 20, 33, 37 eV, respectively). Its 1-hydroxy derivative, compound 4-(1-butyl)-4,5-dihydro-7-hydroxy-*N*-(1-hydroxyadamantan-3-yl)-2-methyl-5-oxo-2H-pyrazolo[4,3-*b*]pyridine-6-carboxamide [33], was employed as Internal Standard: *m*/*z* = 415.2 [M+H]^+^ → *m*/*z* = 266.1, 248.1 (TL = 99 V; CE = 14, 31 eV). Calibration curves were prepared in MeOH in the 1000–10 nM concentration range starting and coefficients of determination of regression models were >0.99. Xcalibur software version 2.2 (Thermo Fisher Scientific, Inc., Waltham, MA, USA) was employed for both data acquisition and processing.

### 3.4. In Vitro Pharmacology

#### 3.4.1. Competition Binding Assay

Binding at equilibrium was performed as previously reported [49]. Briefly, membranes from HEK-293 cells over-expressing the respective human recombinant CB1 receptor (Bmax = 2.5 pmol/mg protein) and human recombinant CB2 receptor (Bmax = 4.7 pmol/mg protein) were incubated with [^3^H]-CP-55,940 (0.14 nM/Kd = 0.18 nM and 0.084 nM/Kd = 0.31 nM, respectively, for CB1 and CB2 receptor) as the high-affinity ligand. Competition curves were performed by displacing [^3^H]-CP-55,940 with increasing concentration of compounds (0.1–10 μM). Nonspecific binding was defined by 10 µM of WIN55,212-2 as the heterologous competitor (*K*_i_ values 9.2 nM and 2.1 nM, respectively, for CB1 and CB2 receptors). All compounds were tested following the procedure described by the manufacturer (Perkin Elmer, Italy). Displacement curves were generated by incubating compounds with [^3^H]-CP-55,940 for 90 min at 30 °C. *K*_i_ values were calculated by applying the Cheng–Prusoff equation to the IC_50_ values (obtained by GraphPad) for the displacement of the bound radioligand by increasing concentrations of the test compound. Data represent mean values for at least three separate experiments performed in duplicate and are expressed as *K*_i_ (nM), average SEM < 5%.

#### 3.4.2. Functional Activity at CB2 Receptor In Vitro

The cAMP Hunter™ assay enzyme fragment complementation chemiluminescent detection kit was used to characterize the functional activity in CB2 receptor-expressing cell lines, as previously reported [67]. Gi-coupled cAMP modulation was measured following the manufacturer’s protocol (DiscoveRx, Fremont, CA, USA). Briefly, CHO-K1 cells overexpressing the human CB2 receptor were plated into a 96-well plate (10,000 cells/well), and incubated overnight at 37 °C, 5% CO_2_. Media were aspirated and replaced with assay buffer. In the case of *Agonist mode*, cells were treated with dose–response solutions of samples prepared in presence of cell assay buffer containing 25 µM NKH477 solution (a water-soluble analog of Forskolin) to stimulate adenylate cyclase and enhance basal cAMP levels. In the case of *Antagonist mode*, cells were pre-treated (15 min at 37 °C) with a dose–response solution of samples prepared in presence of cell assay buffer and further treated with a challenge of a known CB2 agonist (JWH-133, 4 µM) in the presence of cell assay buffer containing 25 µM NKH477. Following stimulation, cell lysis and cAMP detection were performed as per the manufacturer’s protocol. Luminescence measurements were measured using a GloMax Multi Detection System (Promega, Milan, Italy). Data are reported as mean ± SEM of three independent experiments conducted in triplicate and were normalized considering the NKH477 stimulus alone as 100% of the response. Data were analyzed using PRISM software 9.3.1 (GraphPad Software, Inc., San Diego, CA, USA).

## 4. Conclusions

Our efforts to expand the pyrazolo[4,3-*b*]pyridine-6-carboxamide family have successfully yielded 23 new cannabinoid ligands, 10 of which display remarkable CB2R affinity (*K*_i_ < 50 nM) and also good selectivity (SI > 200) over CB1R. Most of the compounds in the series also exhibit excellent physicochemical properties, in particular lipophilicity values determining LLE values between 5.15 and 6.21, except for compound **18** (LLE = 4.90). In addition, the conversion of **63**, taken as a prototypical exponent of the series, to alkaline salts drastically improved both water solubility and dissolution rate. Both agonists and inverse agonists/antagonists have been identified in this series of compounds, with functional activity determined mainly or even exclusively by the substitution pattern of the pyrazole ring. It is therefore quite clear that in the future it will be possible to obtain both agonists and inverse agonists/antagonists in this class of compounds by N1 and N2 substitution of the pyrazole ring, respectively. The affinity and selectivity of the new compounds for the CB2 receptor can be achieved by wisely choosing the optimal substituents for the other parts of the molecule using the newly developed 3D-QSAR model, which herein was applied to validate its prediction ability on receptor affinity and selectivity of the new compounds: the calculated *K*_i_ values are in agreement with the experimental *K*_i_ values.

Taken together, these data suggest that the pyrazolo[4,3-*b*]pyridine-6-carboxamides described here represent an excellent starting point for the further chemical optimization of CB2R agonists and inverse agonist/antagonists, as well as a remarkable tool for exploring the involvement of CB2R in various physiopathological conditions.

## Figures and Tables

**Table 1:** CB1R and CB2R affinity values for compounds **13**–**27**, **33**–**35**, **51**–**55**, and **56**–**62** in comparison with compounds **3**, **63**, and SR144528 *^a,b^*.

Compd.	Structure	CB1R *^c^ K*_i_ *^f^* (nM)	CB2R *^d^ K*_i_ *^f^* (nM)	SI *^e^*	log*K*_iCB2_ (nM) Exp.	log*K*_iCB2_ (nM) Pred.
**13**	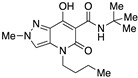	>10,000	>10,000	–	4	3.19
**14**	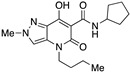	>10,000	>10,000	–	4	3.53
**15**	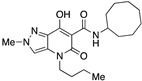	>10,000	9.03 ± 4.84	>1107	1.16	1.6
**16**	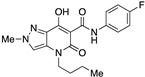	>10,000	>10,000	–	4	4
**17**	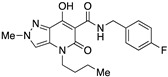	>10,000	>10,000	–	4	3.73
**18**	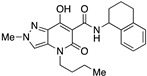	>10,000	17.31 ± 8.31	>578	1.38	1.9
**19**	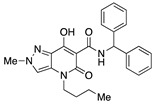	>10,000	142.25 ± 109.73	>70	2.15	2.65
**20**	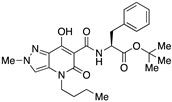	>10,000	>10,000	–	4	4
**21**	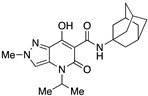	>10,000	>10,000	–	4	3.59
**22**	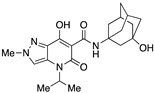	>10,000	>10,000	–	4	3.39
**23**	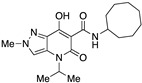	>10,000	>10,000	–	4	3.05
**24**	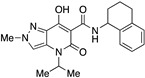	>10,000	>10,000	–	4	3.43
**25**	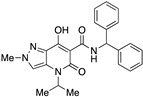	>10,000	>10,000	–	4	4
**26**	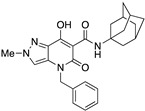	115.63 ± 23.66	0.48 ± 0.12	241	−0.32	1
**27**	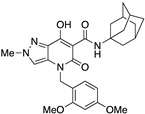	>10,000	>10,000	–	4	3.52
**33**	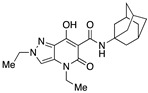	>10,000	24.04 ± 15.34	>416	1.38	1.81
**34**	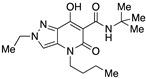	>10,000	105.85 ± 18.12	>95	2.02	1.61
**35**	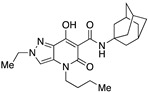	102.30 ± 15.23	0.45 ± 0.01	227	−0.35	0.63
**51**	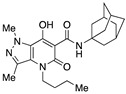	>10,000	35.61 ± 19.46	>280	1.55	1.81
**52**	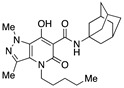	>10,000	34.10 ± 4.88	>293	1.53	2.2
**53**	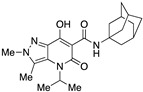	>10,000	>10,000	–	4	3.88
**54**	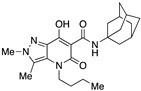	>10,000	2.16 ± 1.7	>4630	0.33	1.2
**55**	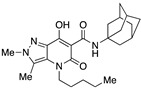	>10,000	2.11 ± 0.74	>4739	0.32	0.69
**56**	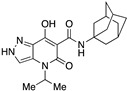	>10,000	>10,000	–	4	3.54
**57**	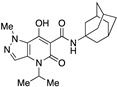	>10,000	54.31 ± 12.28	>184	1.73	2.7
**58**	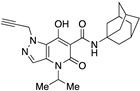	>10,000	61.76 ± 20.78	>162	1.79	1.36
**59**	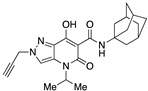	>10,000	>10,000	–	4	2.8
**60**	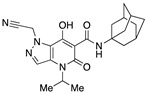	>10,000	48.46 ± 10.94	>206	1.68	2.7
**61**	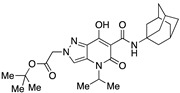	>10,000	>10,000	–	4	4
**62**	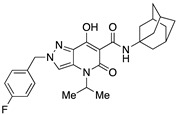	>10,000	>10,000	–	4	4
**63** [34]	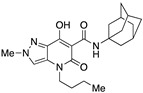	231 ± 76	2.5 ± 0.2	92	–	–
**3** [34]	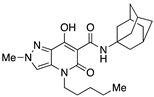	33.5 ± 0.9	0.18 ± 0.01	167	–	–
SR144528 *^g,h^*	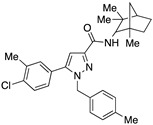	116 ± 22	1.8 ± 0.5	64	–	–

*^a^* Data represent mean values ± SD for at least three separate experiments performed in duplicate and are expressed as *K*_i_ (nM). *^b^* For both receptor binding assays, the new compounds were tested using membranes from HEK cells transfected with either CB1R or CB2R and [^3^H]-(–)-*cis*-3-[2-hydroxy-4-(1,1-dimethylheptyl)phenyl]-*trans*-4-(3-hydroxypropyl)cyclohexanol ([^3^H]CP-55,940). *^c^* CB1R: human cannabinoid type 1 receptor. *^d^* CB2R: human cannabinoid type 2 receptor. *^e^* SI: selectivity index for CB2R, calculated as *K*_i_(CB1R)/*K*_i_(CB2R) ratio. *^f^ K*_i_: inhibitor constant; that is, the concentration of the competing ligand that will bind to half the binding sites at equilibrium in the absence of radioligand or other competitors. *^g^* CB2 reference compound. *^h^* The binding affinities of reference compounds were evaluated in parallel with test compounds under the same conditions.

## Data Availability

Data are contained within the article.

## Supplementary Materials

The following supporting information can be downloaded at: https://www.mdpi.com/article/10.3390/molecules28134958/s1, Figure S1: X-ray crystal structure of compound **51**; Figure S2: Bioavailability radar plot of selected compounds using the SwissADME software; Figures S3-S48: NMR spectra of compounds **13**–**27**, **33**–**35**, **51**–**55**; Table S1: Calculated physicochemical and drug-like properties of selected compounds.

## Sample Availability

Not available.
