# Supplementary material for: Systematic Modification of the Substitution Pattern of the 7-Hydroxy-5-oxopyrazolo[4,3-b]pyridine-6-carboxamide Scaffold Enabled the Discovery of New Ligands with High Affinity and Selectivity for the Cannabinoid Type 2 Receptor
Source: Molecules. 2023 Jun 24;28(13):4958. doi: 10.3390/molecules28134958 (PMC10343475; doi:10.3390/molecules28134958)
Supplement: Supplementary file 1 [file molecules-28-04958-s001.zip › molecules-2446410-supplementary.pdf]

Article

Systematic Modification of the Substitution Pattern of the 7-Hydroxy-5-oxopyrazolo[4,3-*b*]pyridine-6-carboxamide Scaffold Enabled the Discovery of New Ligands with High Affinity and Selectivity for the Cannabinoid Type 2 Receptor

Claudia Mugnaini [1,*], Magdalena Kostrzewa [2], Marta Casini [1], Poulami Kumar [2], Valeria Catallo [1], Marco Allarà [2], Laura Guastaferro [1], Antonella Brizzi [1], Marco Paolino [1], Andrea Tafi [1], Christelos Kapatais [1], Gianluca Giorgi [1], Federica Vacondio [3], Marco Mor [3], Federico Corelli [1] and Alessia Ligresti [2,*]

[1] Department of Biotechnology, Chemistry and Pharmacy, University of Siena, 53100 Siena, Italy; marta.casini94@gmail.com (M.C.); valeriacatallo@gmail.com (V.C.); guastaferro.laura@gmail.com (L.G.); antonella.brizzi@unisi.it (A.B.); paolino3@unisi.it (M.P.); andrea.tafi@unisi.it (A.T.); kapatais@student.unisi.it (C.K.); gianluca.giorgi@unisi.it (G.G.); federico.corelli@unisi.it (F.C.)

[2] National Research Council of Italy, Institute of Biomolecular Chemistry, 80078 Pozzuoli, Italy; m.kostrzewka@gmail.com (M.K.); p.kumar@icb.cnr.it (P.K.); mallara@icb.cnr.it (M.A.)

[3] Department of Food and Drug, University of Parma, Parco Area delle Scienze 27/A, 43124 Parma, Italy; federica.vacondio@unipr.it (F.V.); marco.mor@unipr.it (M.M.)

* Correspondence: claudia.mugnaini@unisi.it (C.M.); alessia.ligresti@icb.cnr.it (A.L.)

Supplementary Information

Content page

X-Ray crystallographic analysis of compound **51** 2

Bioavailability radar plot of selected compounds 3

NMR spectra of the final compounds **13-27**, **33-35**, **51-55** 4-26

Calculated physicochemical and druglike properties of selected compounds 27

X-Ray crystallographic analysis of compound 51

A single crystal of compound **51** was submitted to X-ray data collection on an Oxford-Diffraction Xcalibur Sapphire 3 diffractometer with a graphite monochromated Mo-Kα radiation (λ = 0.71073 Å) at 293 K. The structure was solved by direct methods implemented in SHELXS-97 program [1]. The refinement was carried out by full-matrix anisotropic least-squares on F^2 for all reflections for non-H atoms by means of the SHELXL-97 program [2]. Crystallographic data have been deposited with the Cambridge Crystallographic Data Centre as supplementary publication no. 2265135. Copies of the data can be obtained, free of charge, on application to CCDC, 12 Union Road, Cambridge CB2 1EZ, UK; (fax: + 44 (0) 1223 336 033; or e- mail: deposit@ccdc.cam.ac.uk).

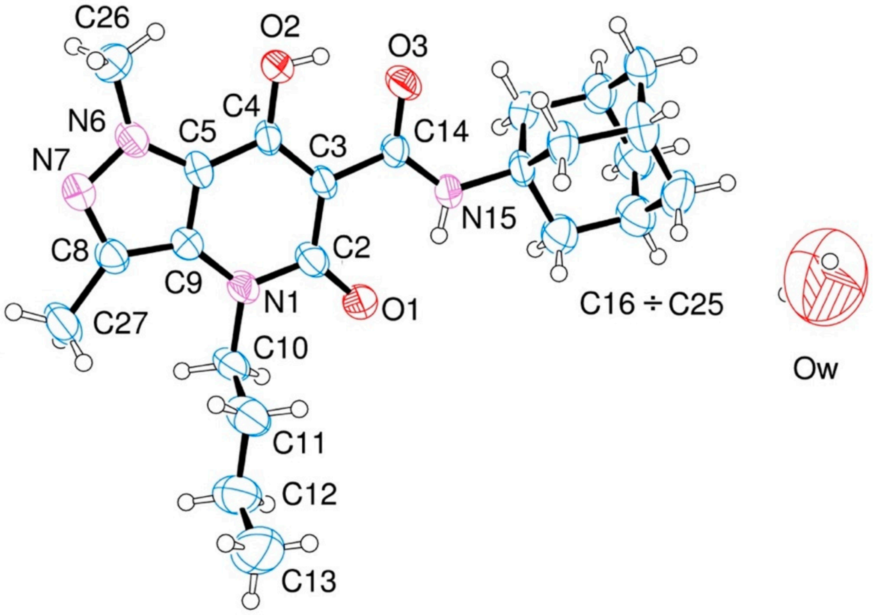

Figure S1. X-ray crystal structure of compound **51**. Ellipsoids enclose 50% probability

References

[1] Sheldrick, G. M. SHELXS-97, Rel. 97-2, A Program for Automatic Solution of Crystal Structures, University of Göttingen, Göttingen (Germany), 1997.
[2] Sheldrick, G. M. Crystal structure refinement with SHELXL. *Acta Cryst*. **2015**, *C71*, 3-8.

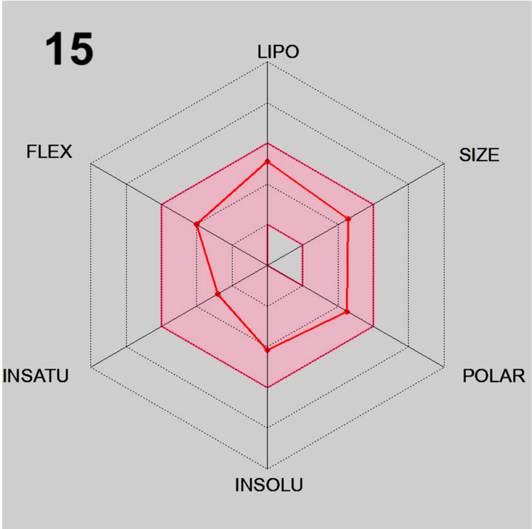

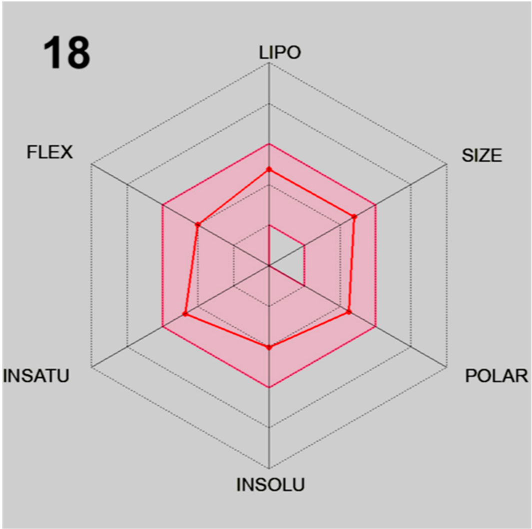

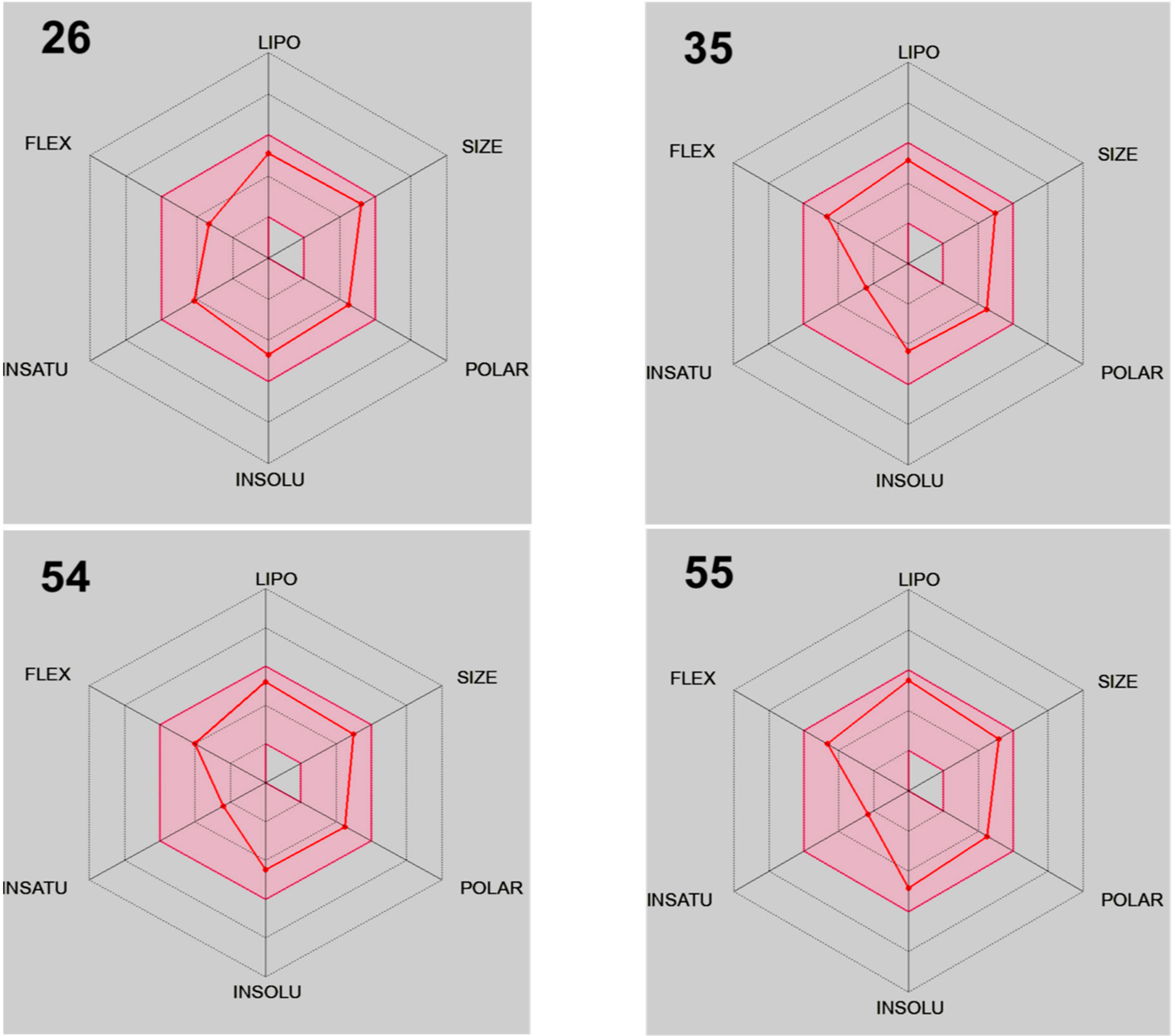

Figure S2. Bioavailability radar plot of selected compounds using the SwissADME software

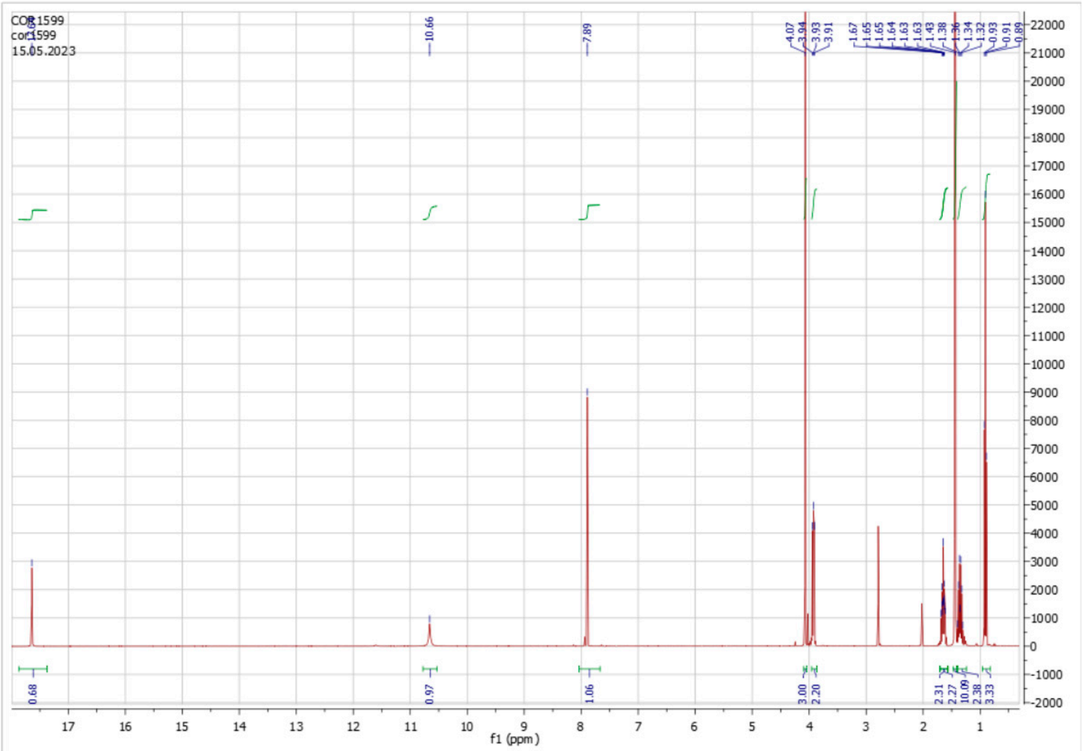

Figure S3. ^{1}H NMR spectrum of compound **13**.

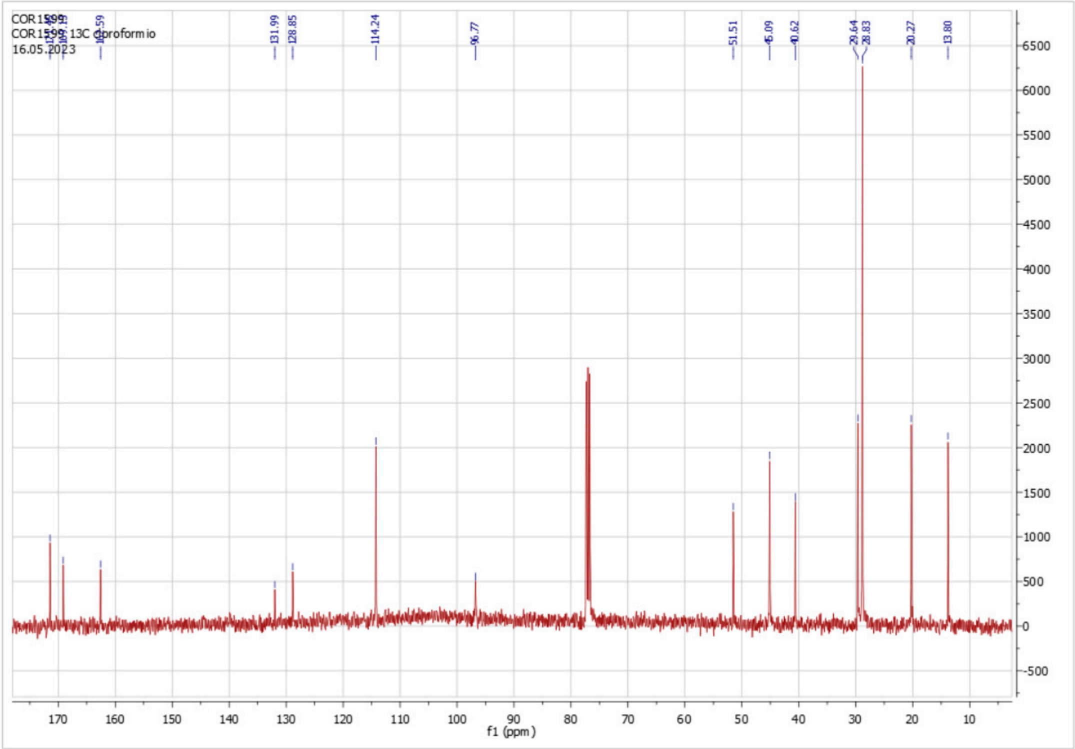

Figure S4. ^{13}C NMR spectrum of compound **13**.

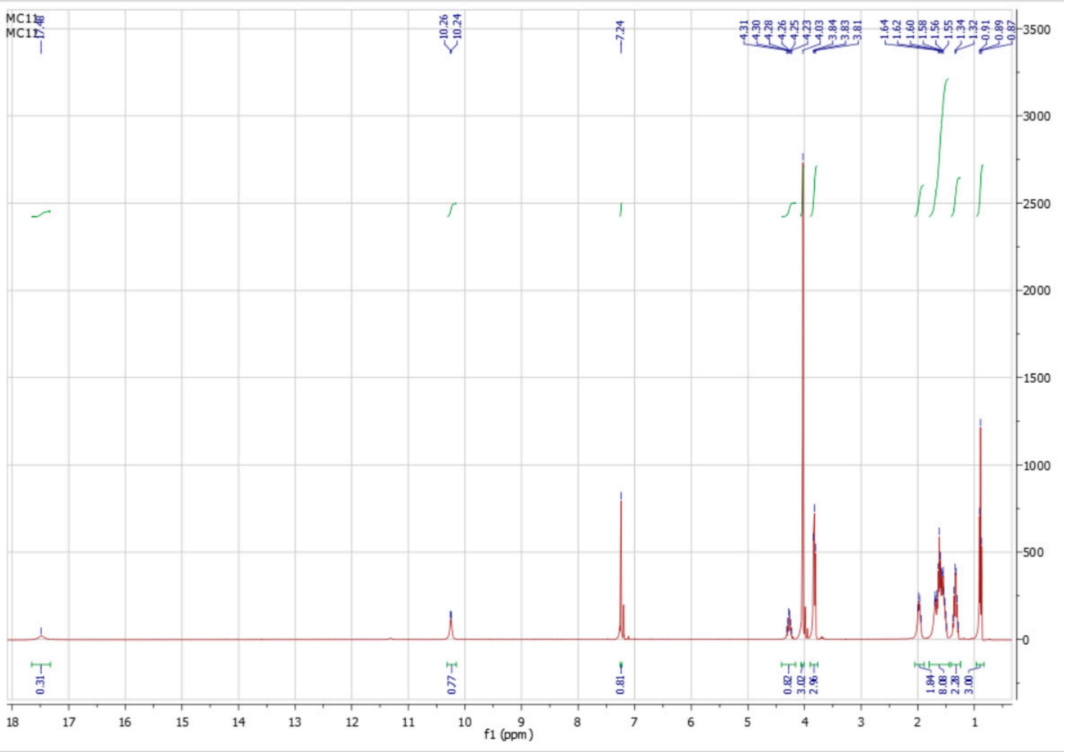

Figure S5. ^{1}H NMR spectrum of compound **14**.

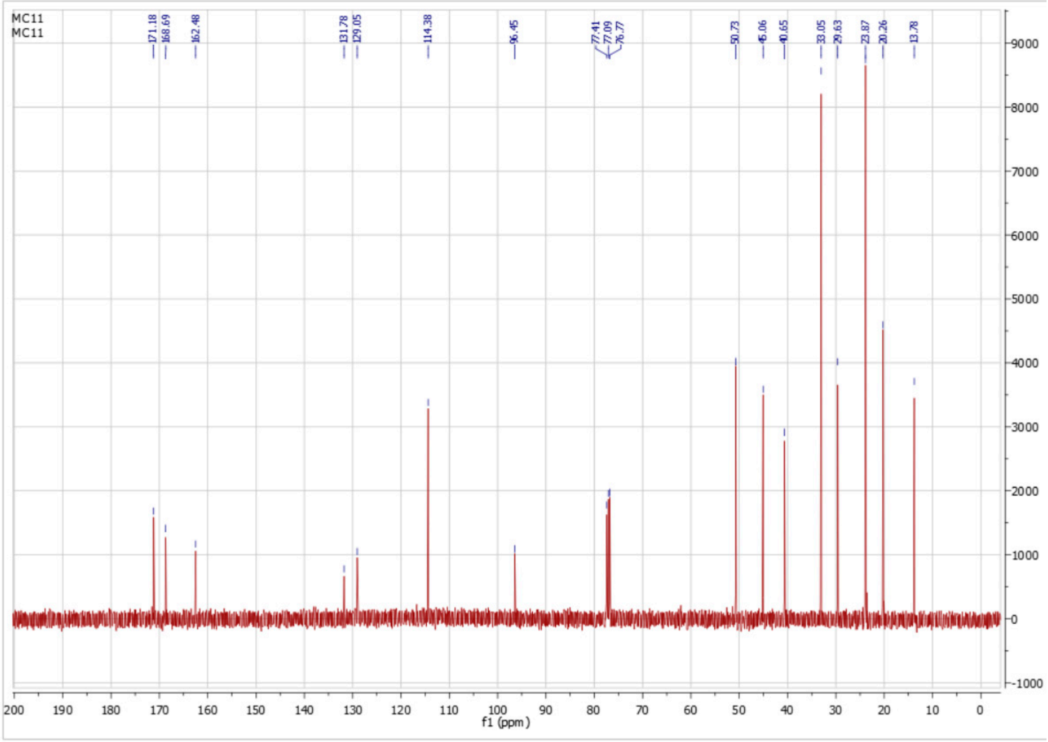

Figure S6. ^{13}C NMR spectrum of compound **14**.

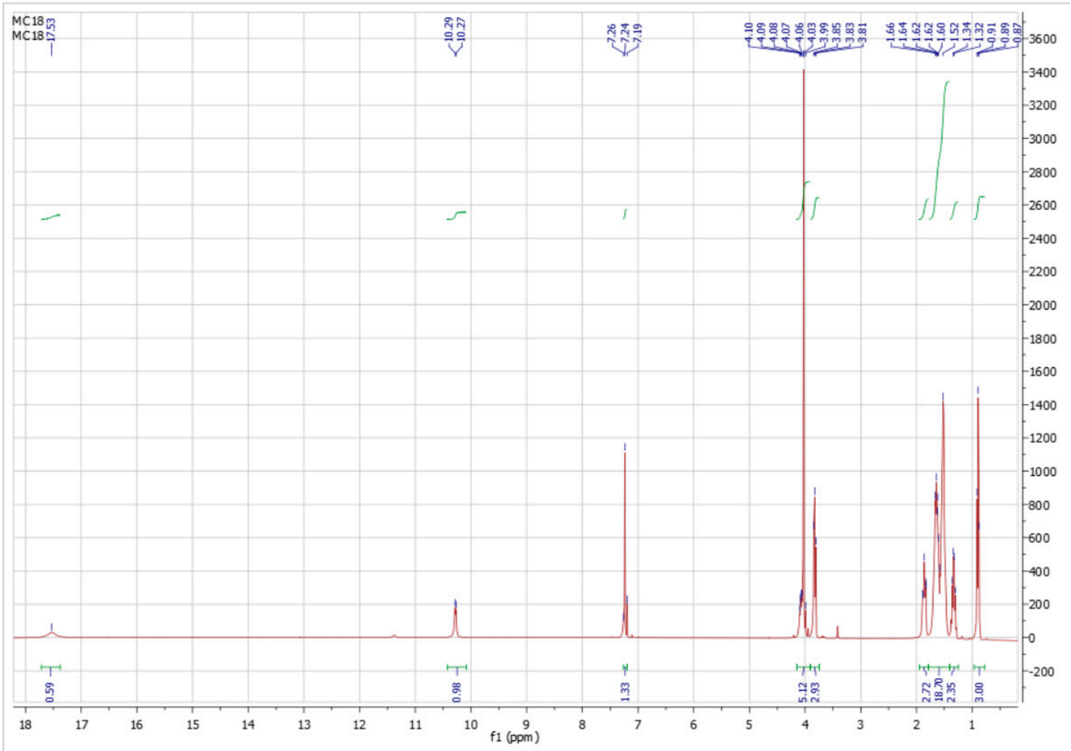

Figure S7. ^{1}H NMR spectrum of compound **15**.

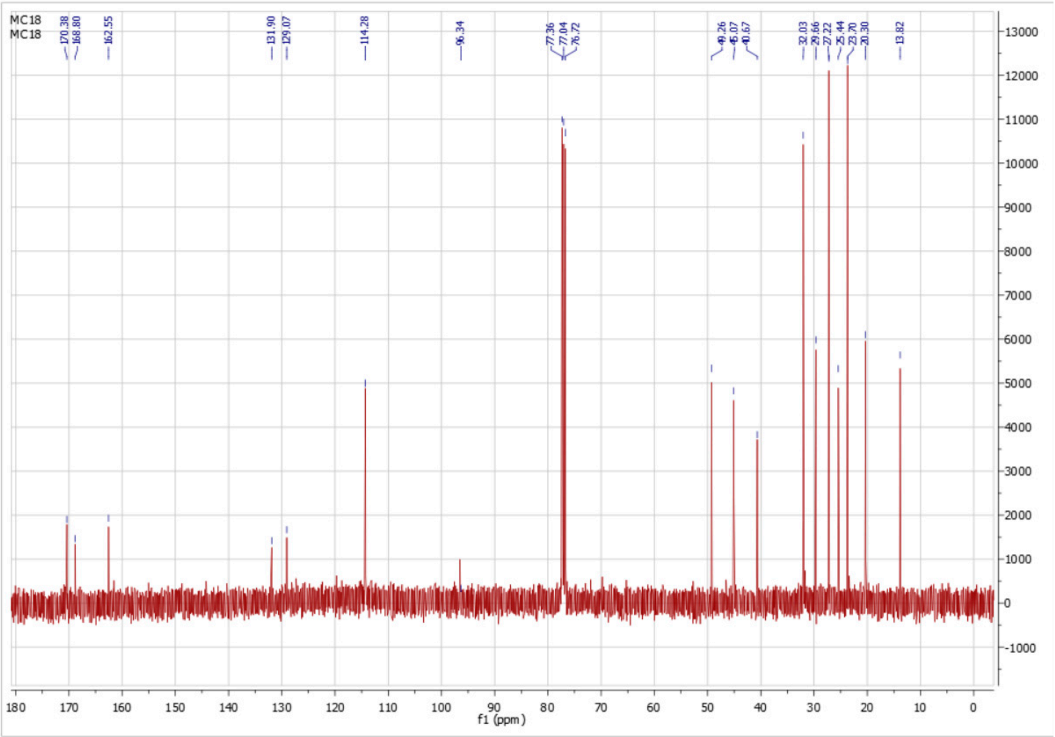

Figure S8. ^{13}C NMR spectrum of compound **15**.

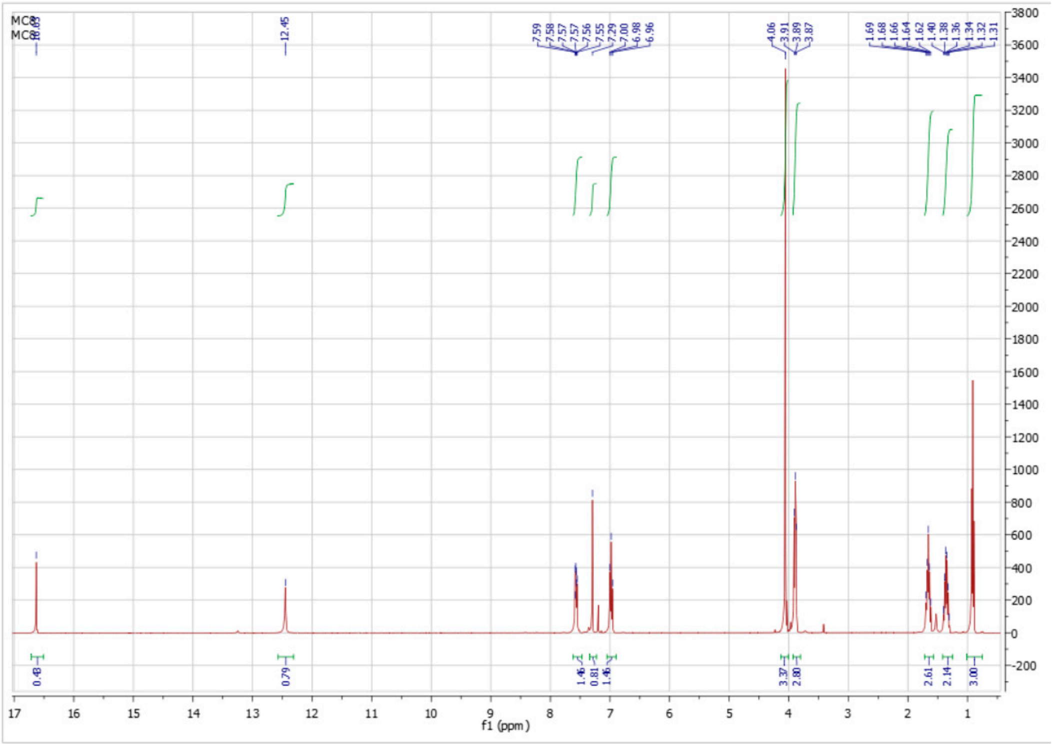

Figure S9. ^{1}H NMR spectrum of compound **16**.

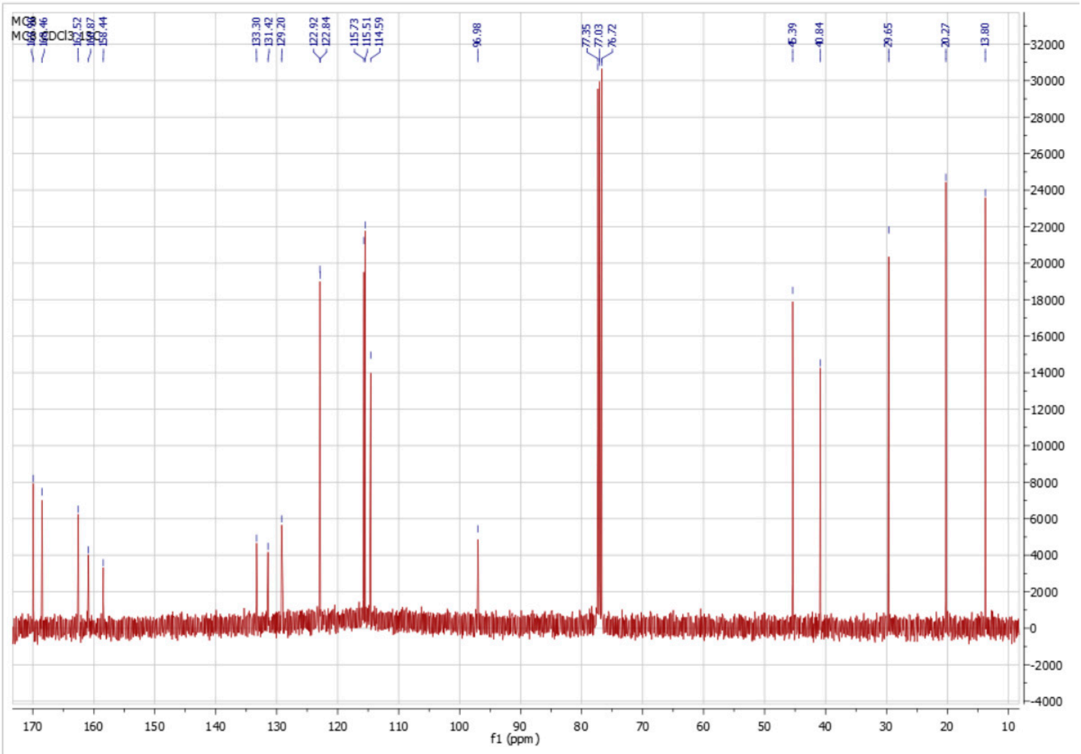

Figure S10. ^{13}C NMR spectrum of compound **16**.

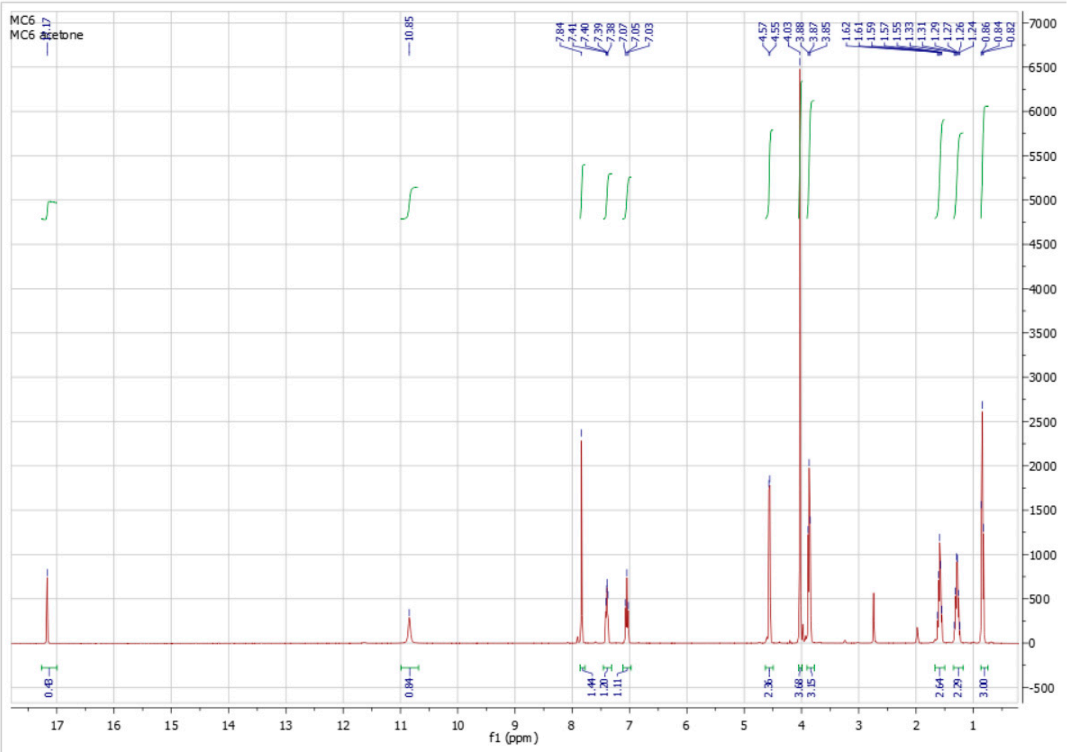

Figure S11. ^{1}H NMR spectrum of compound **17**.

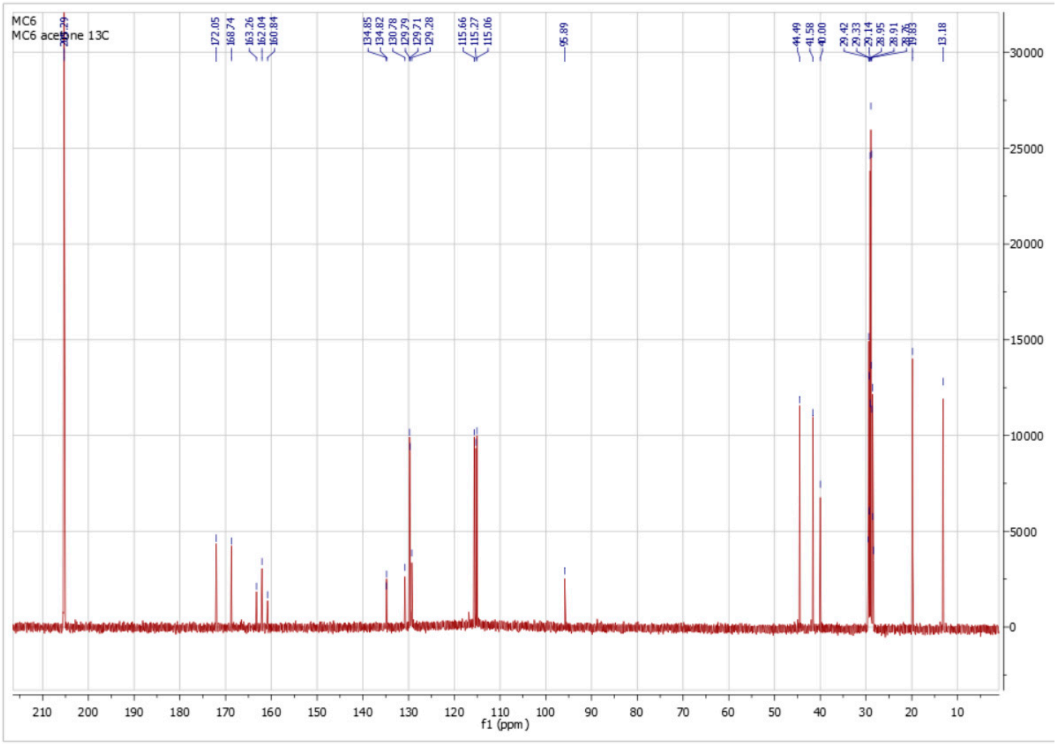

Figure S12. ^{13}C NMR spectrum of compound **17**.

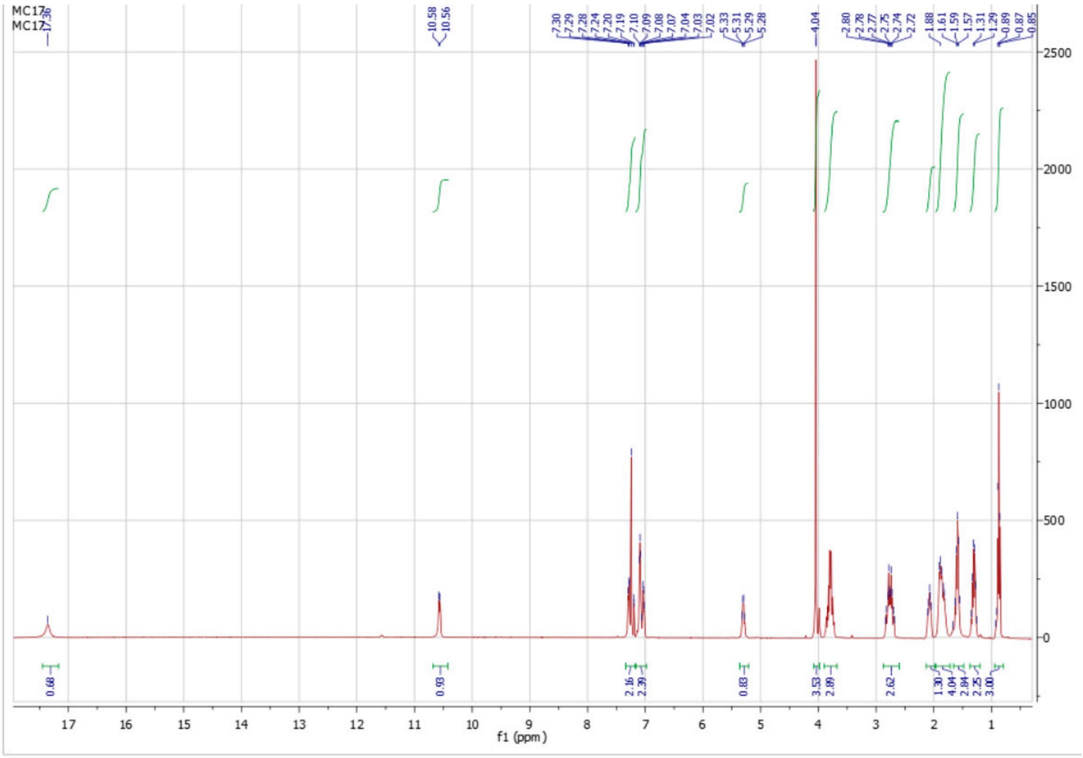

Figure S13. ^{1}H NMR spectrum of compound **18**.

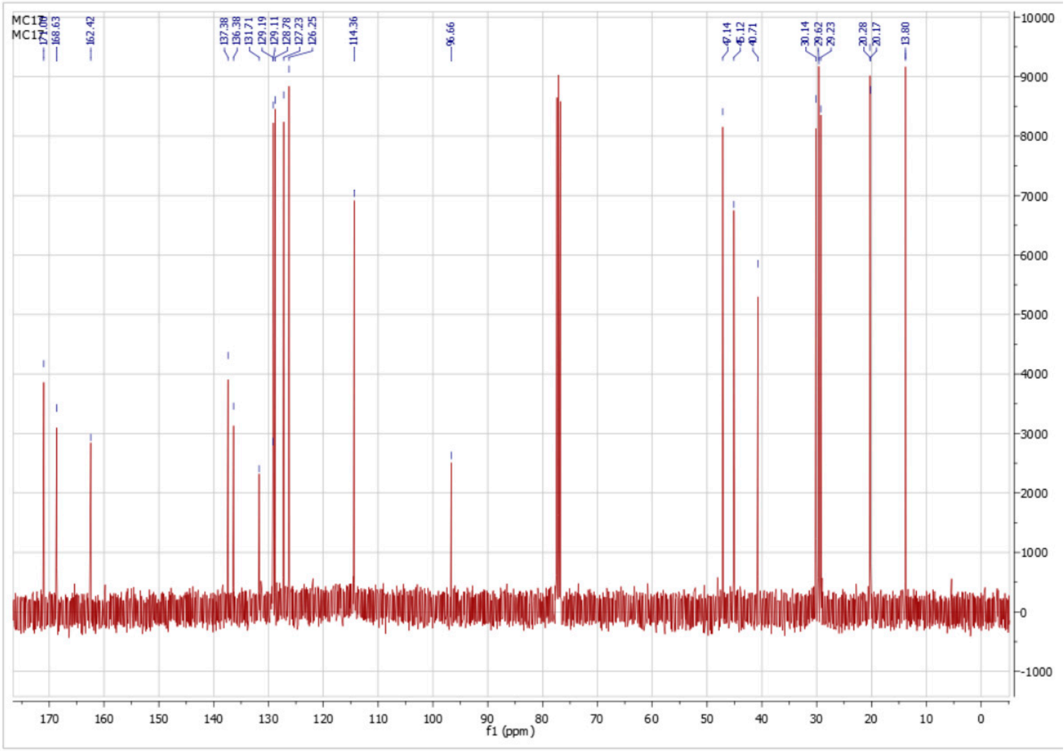

Figure S14. ^{13}C NMR spectrum of compound **18**.

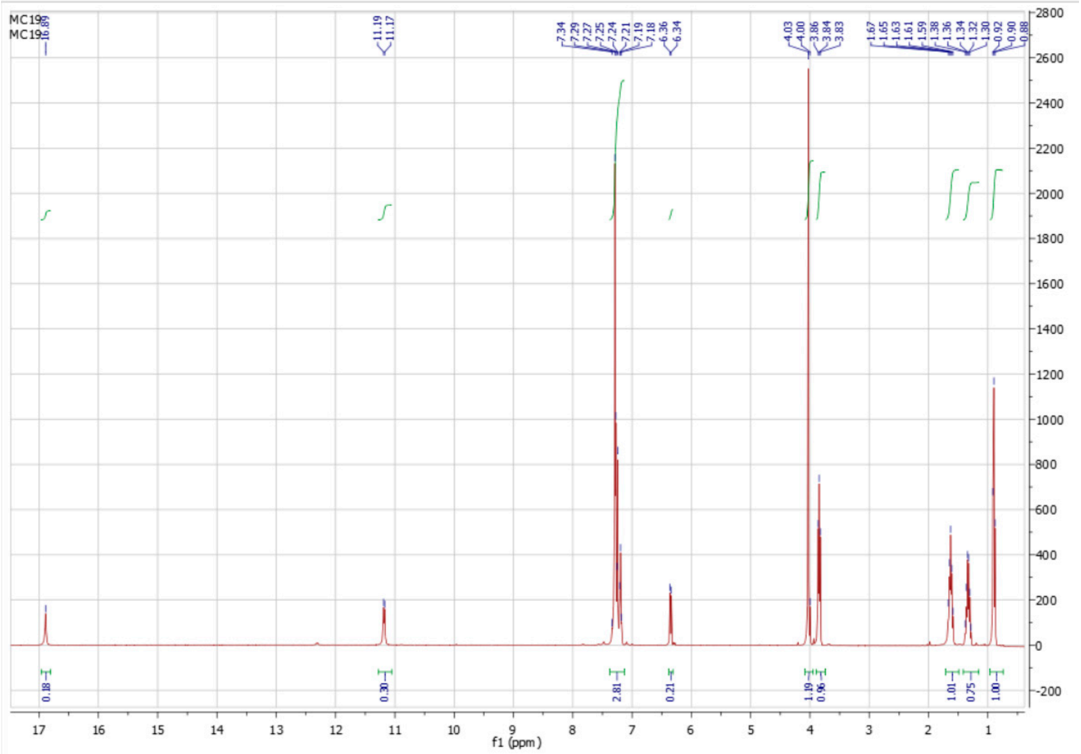

Figure S15. ^{1}H NMR spectrum of compound **19**.

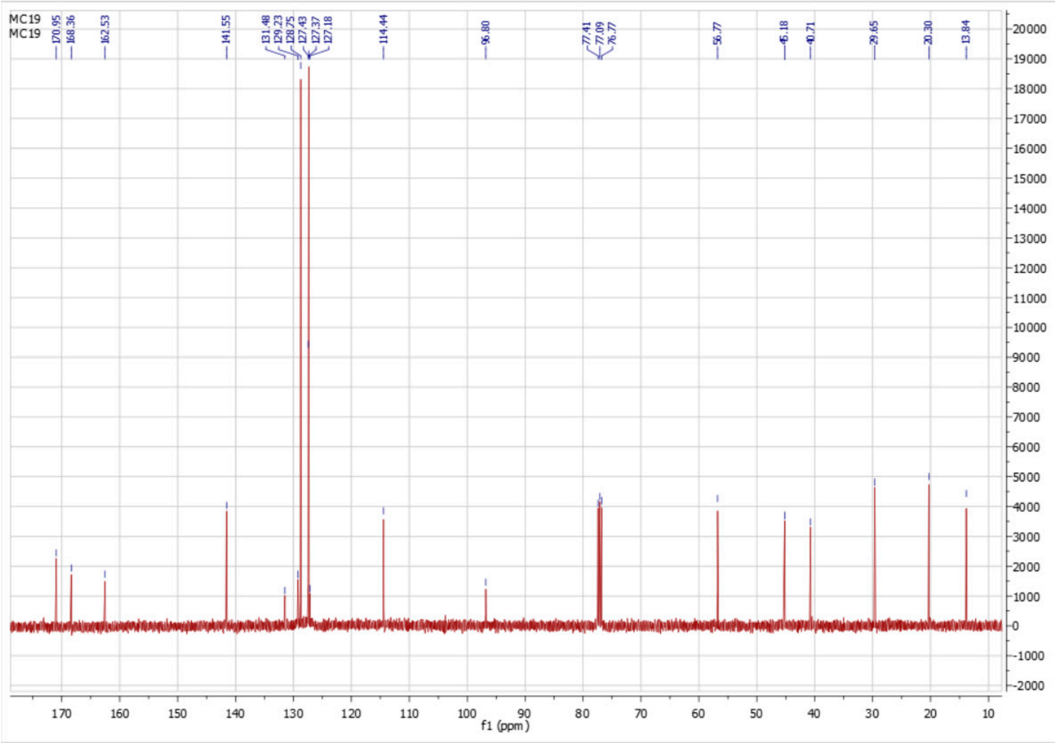

Figure S16. ^{13}C NMR spectrum of compound **19**.

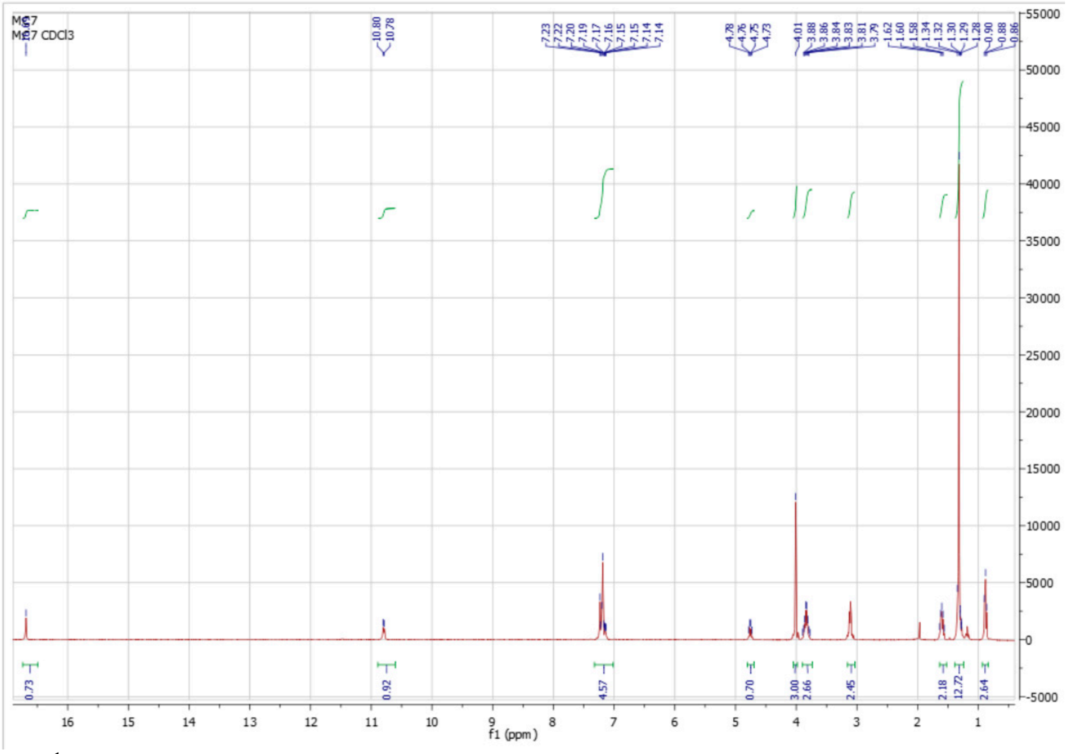

Figure S17. ^{1}H NMR spectrum of compound **20**.

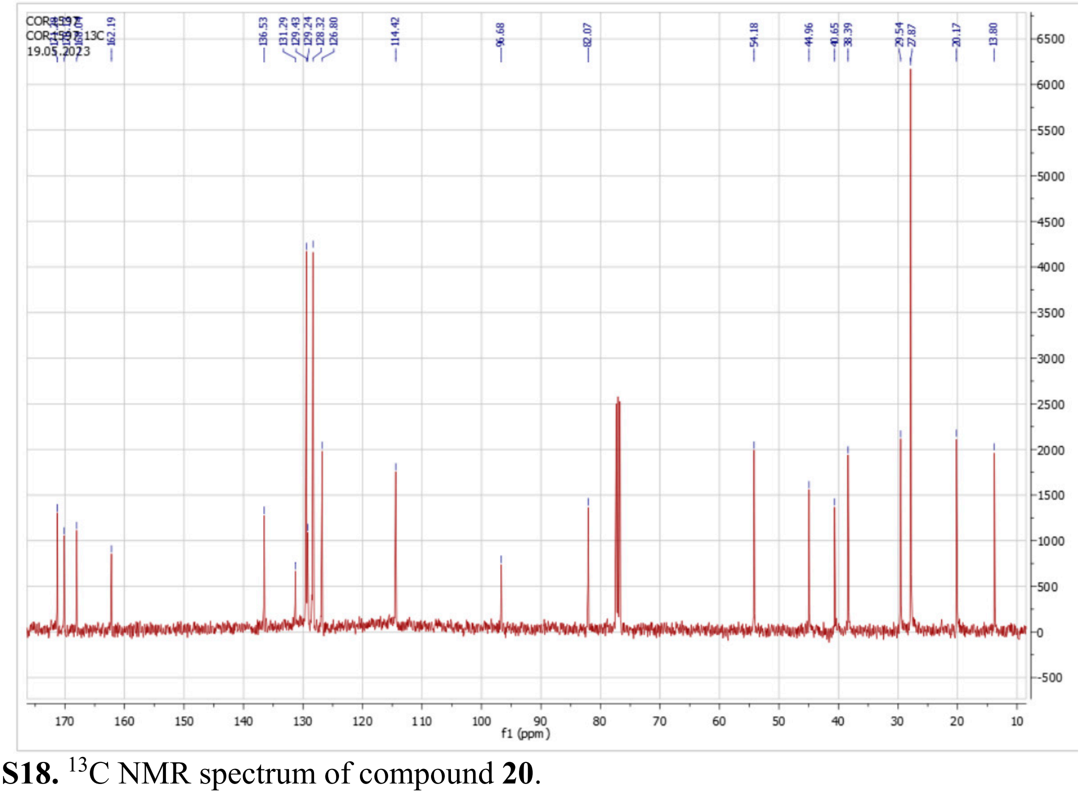

Figure S18. ^{13}C NMR spectrum of compound **20**.

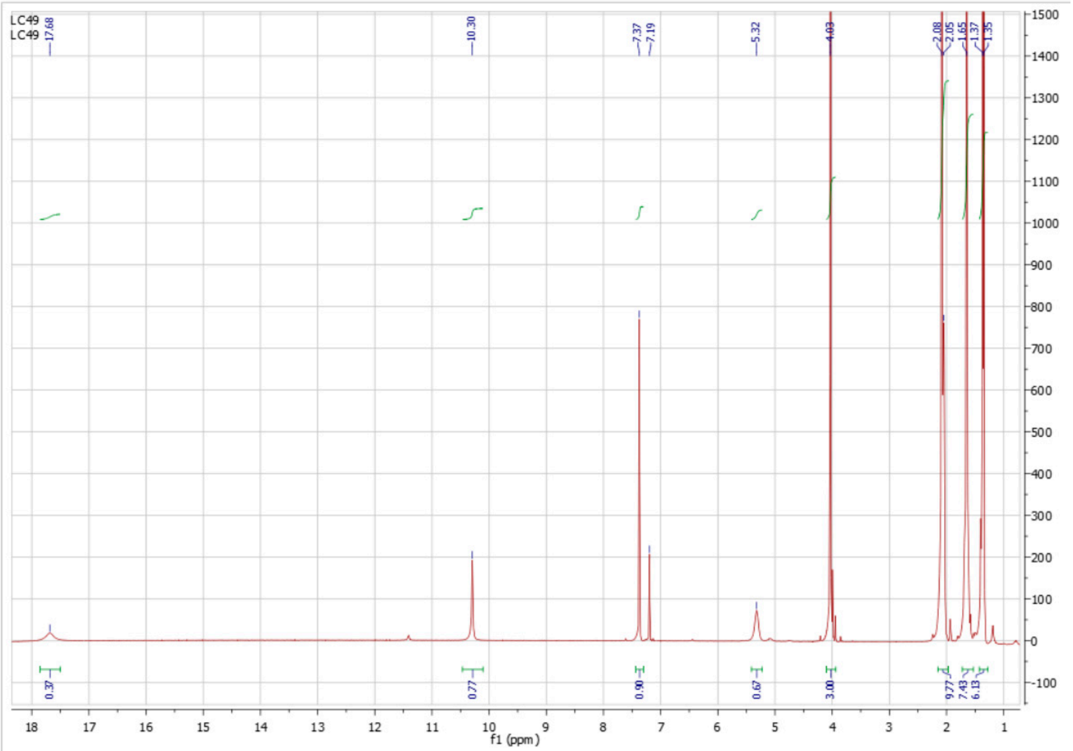

Figure S19. ^{1}H NMR spectrum of compound **21**.

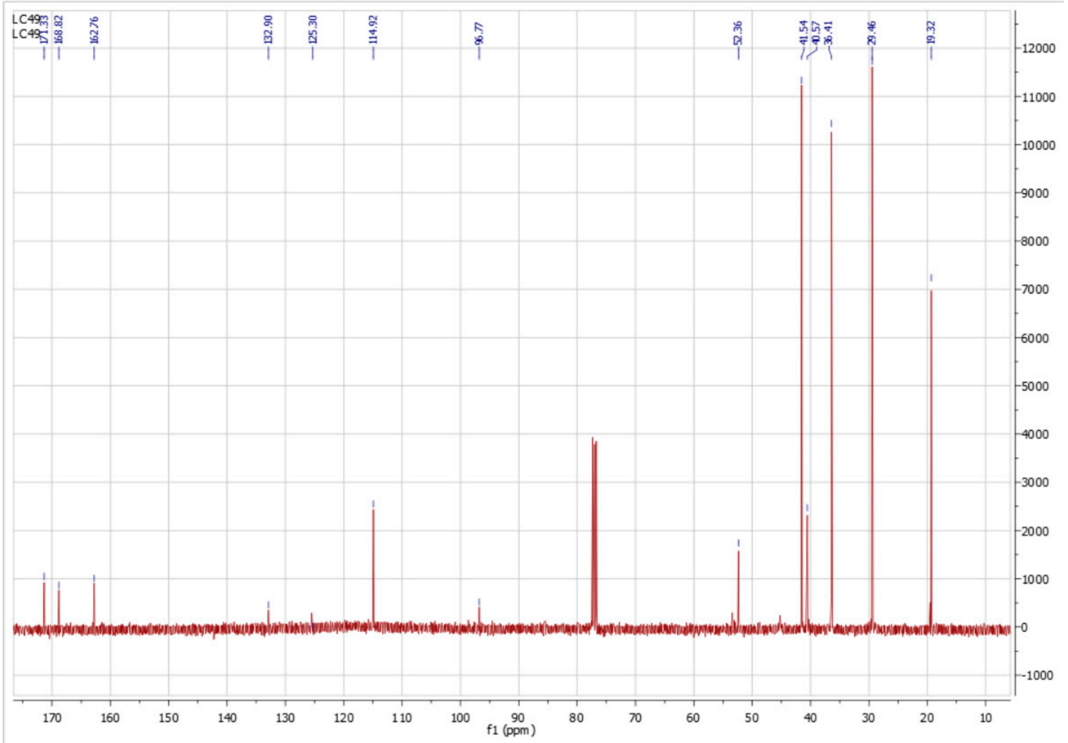

Figure S20. ^{13}C NMR spectrum of compound **21**.

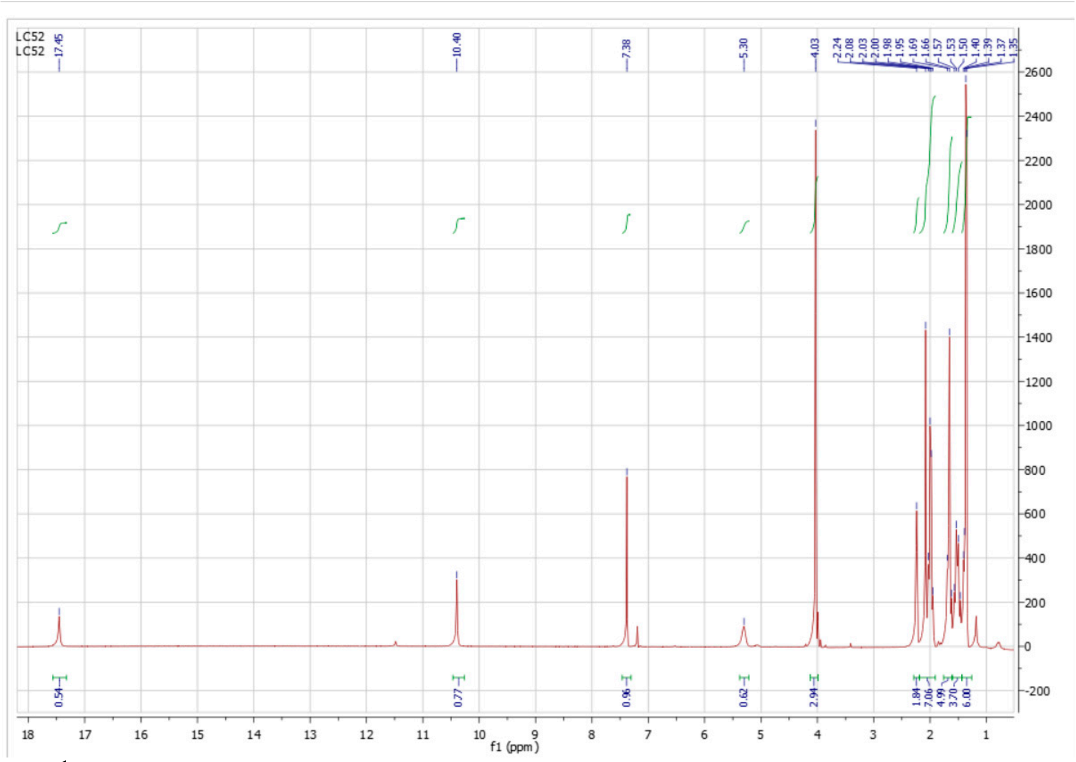

Figure S21. ^{1}H NMR spectrum of compound **22**.

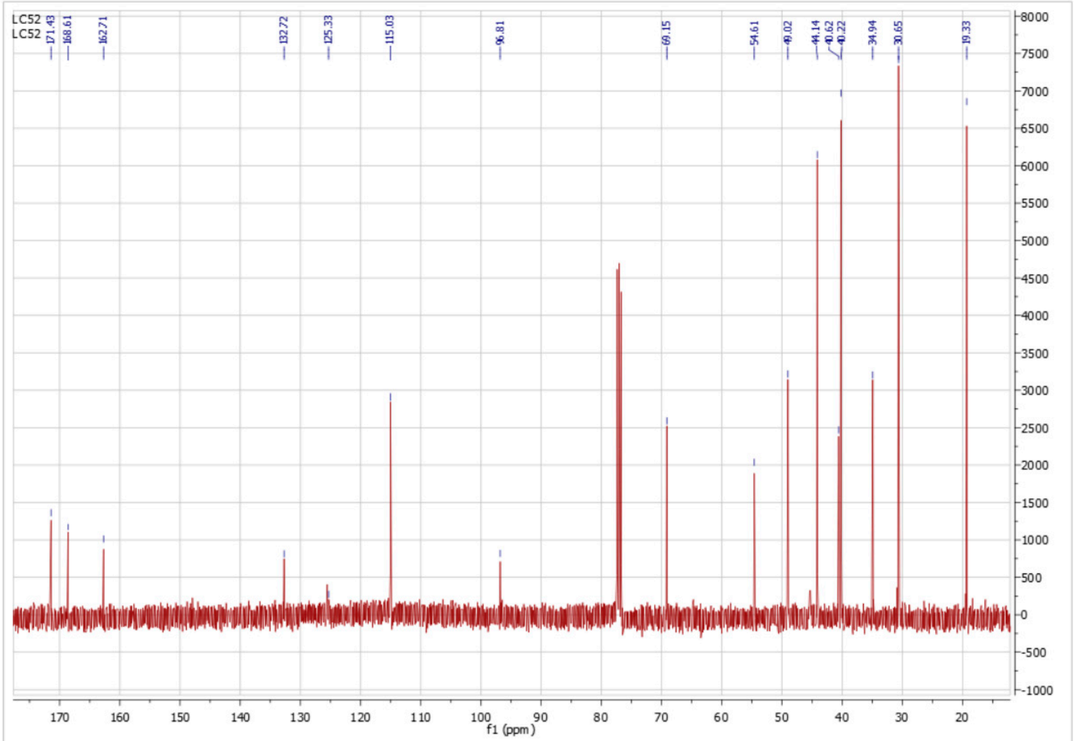

Figure S22. ^{13}C NMR spectrum of compound **22**.

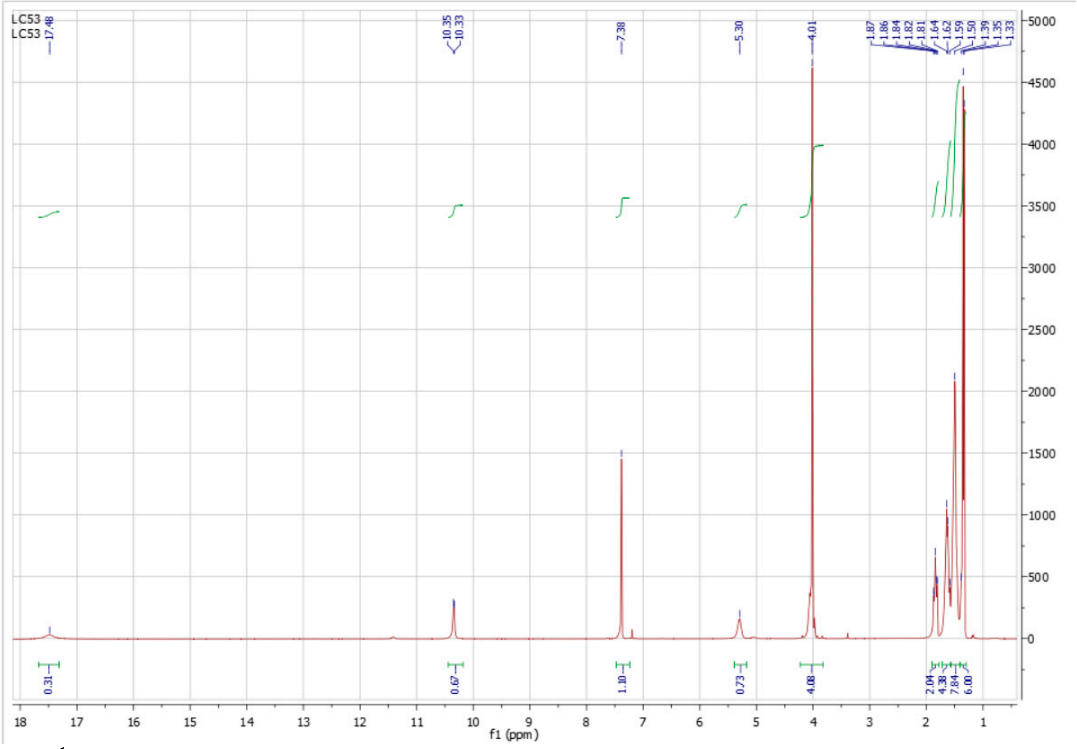

Figure S23. ^{1}H NMR spectrum of compound **23**.

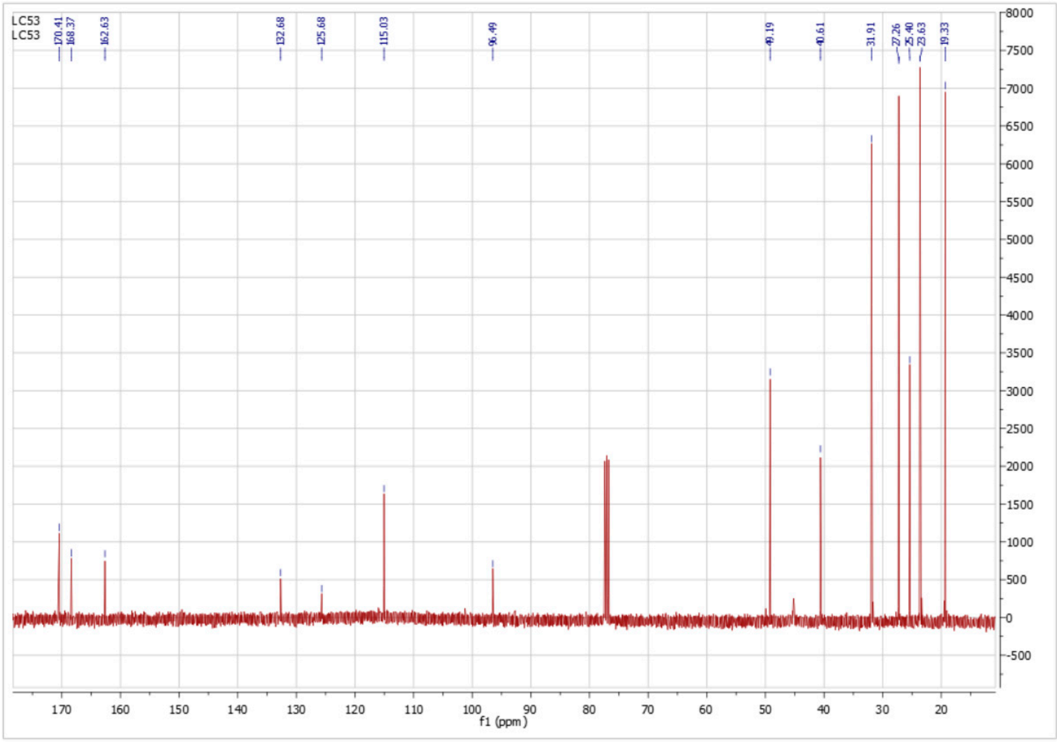

Figure S24. ^{13}C NMR spectrum of compound **23**.

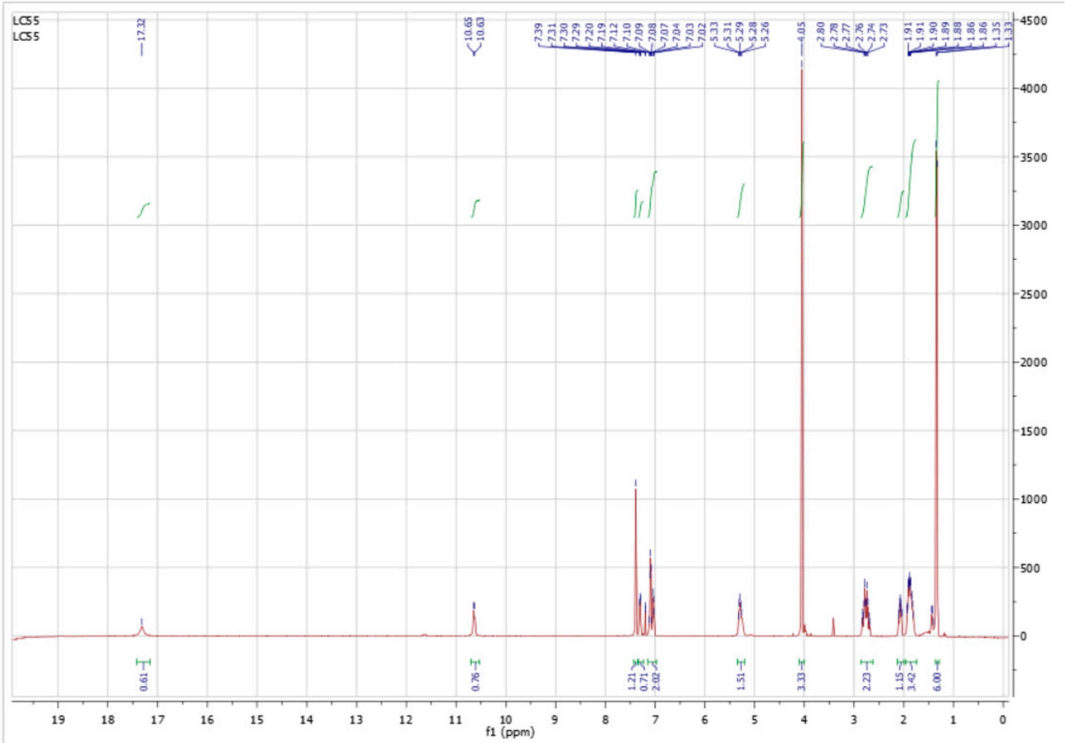

Figure S25. ^{1}H NMR spectrum of compound **24**.

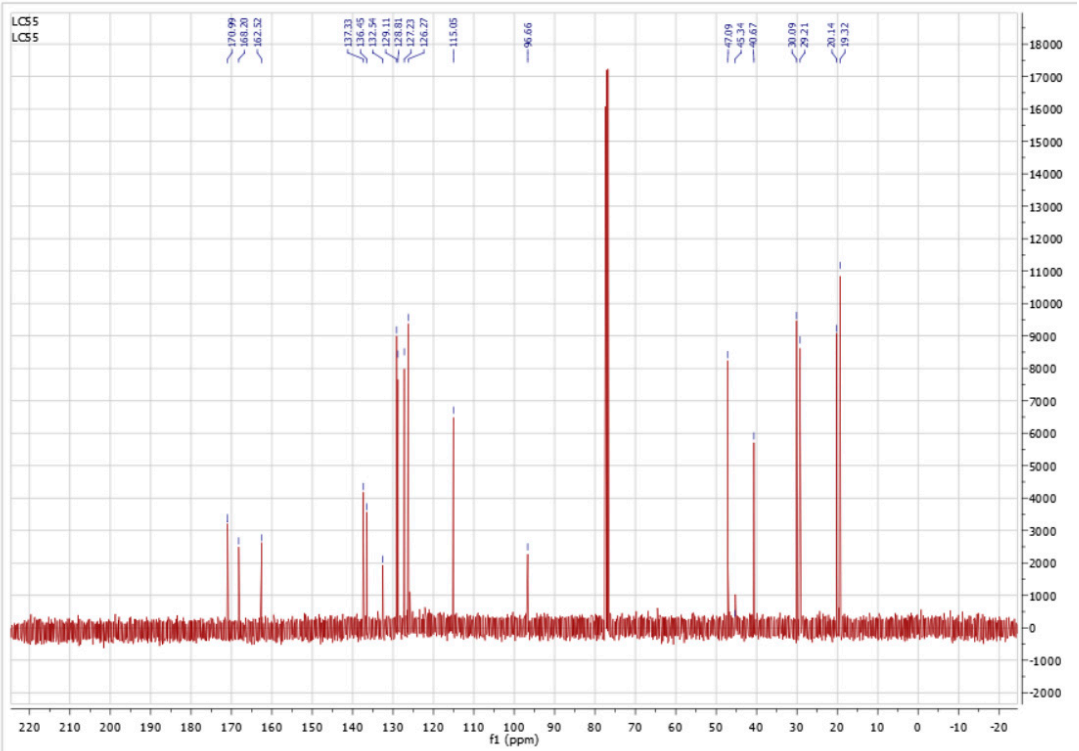

Figure S26. ^{13}C NMR spectrum of compound **24**.

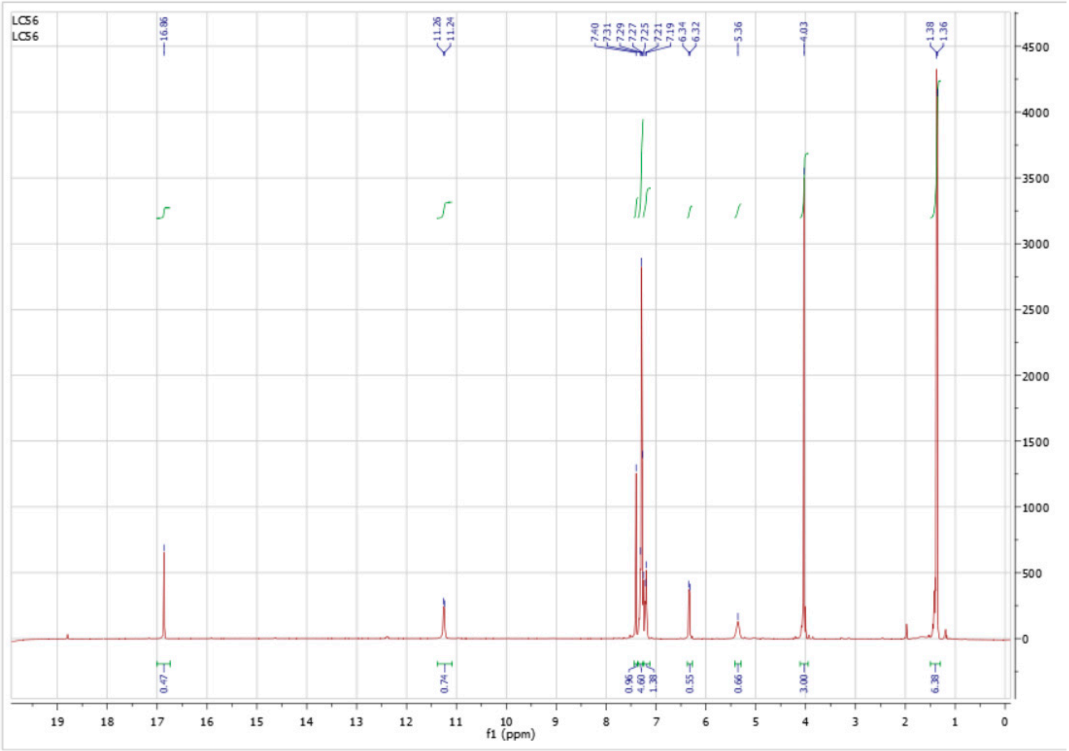

Figure S27. ^{1}H NMR spectrum of compound **25**.

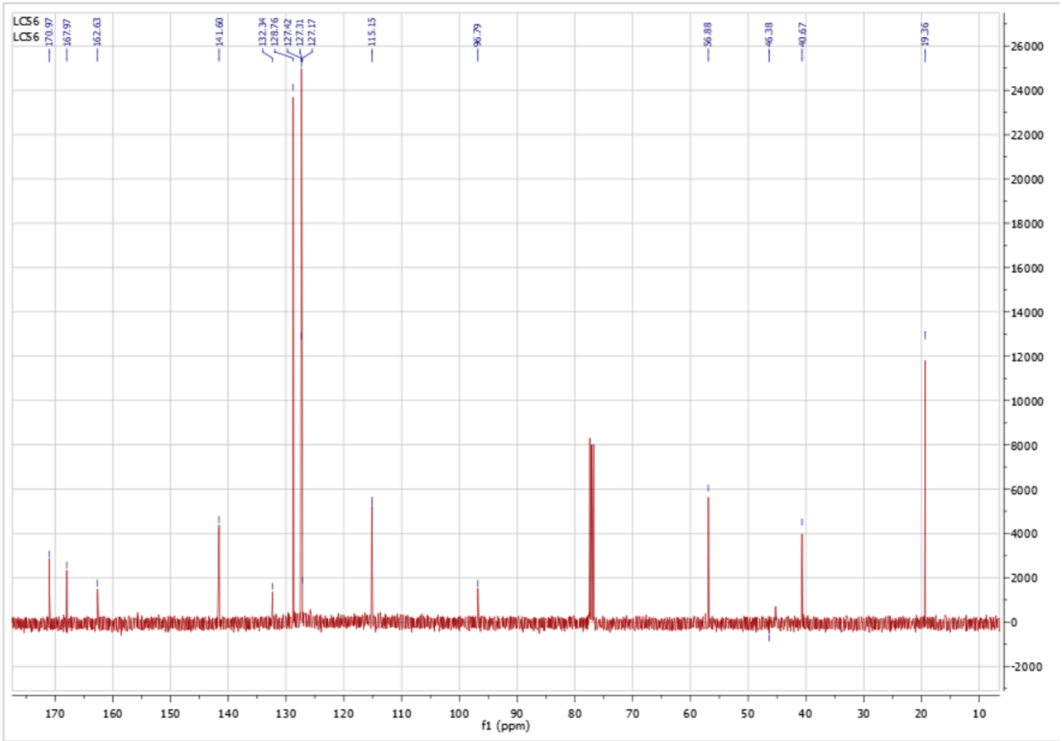

Figure S28. ^{13}C NMR spectrum of compound **25**.

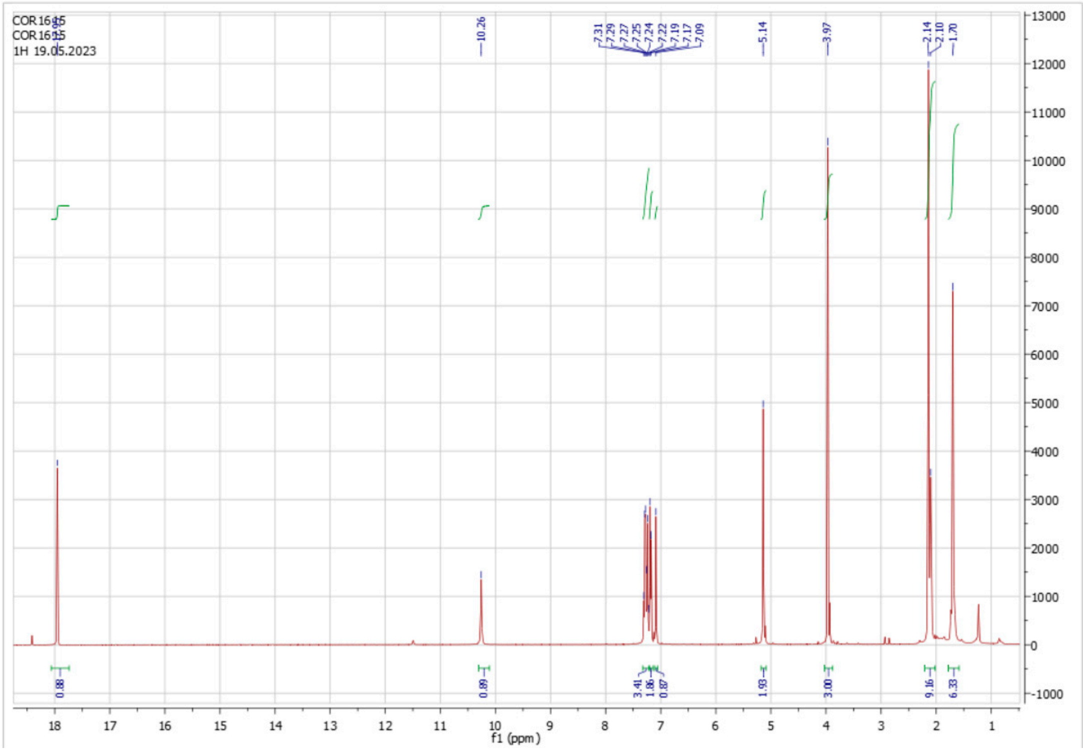

Figure S29. ^{1}H NMR spectrum of compound **26**.

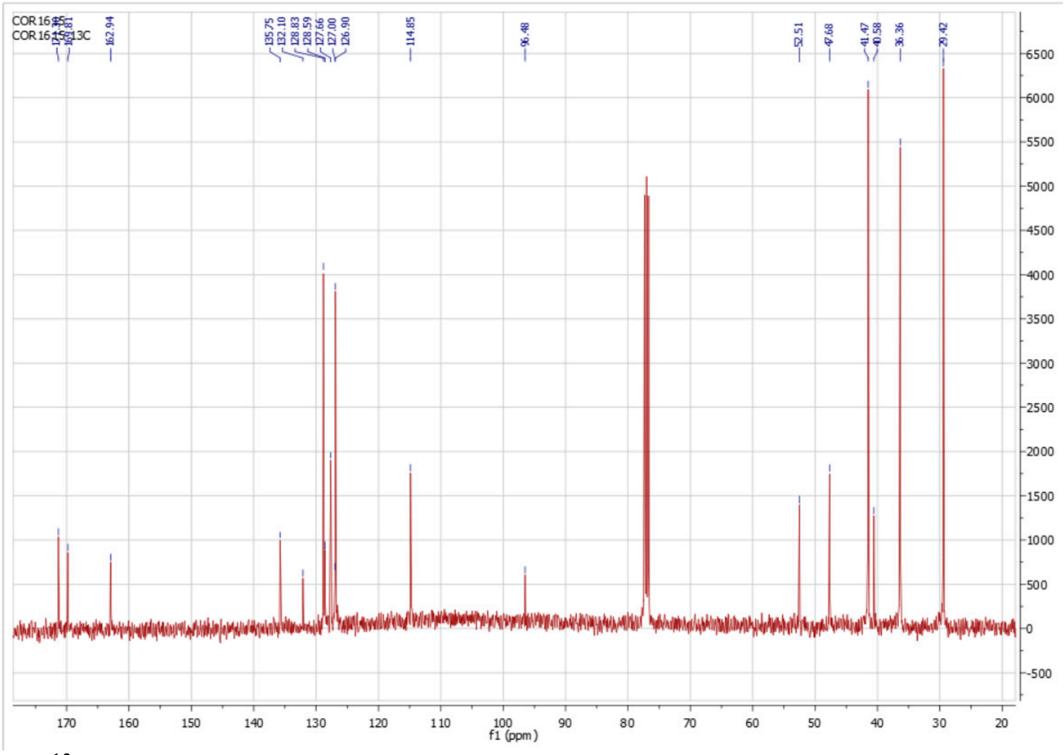

Figure S30. ^{13}C NMR spectrum of compound **26**.

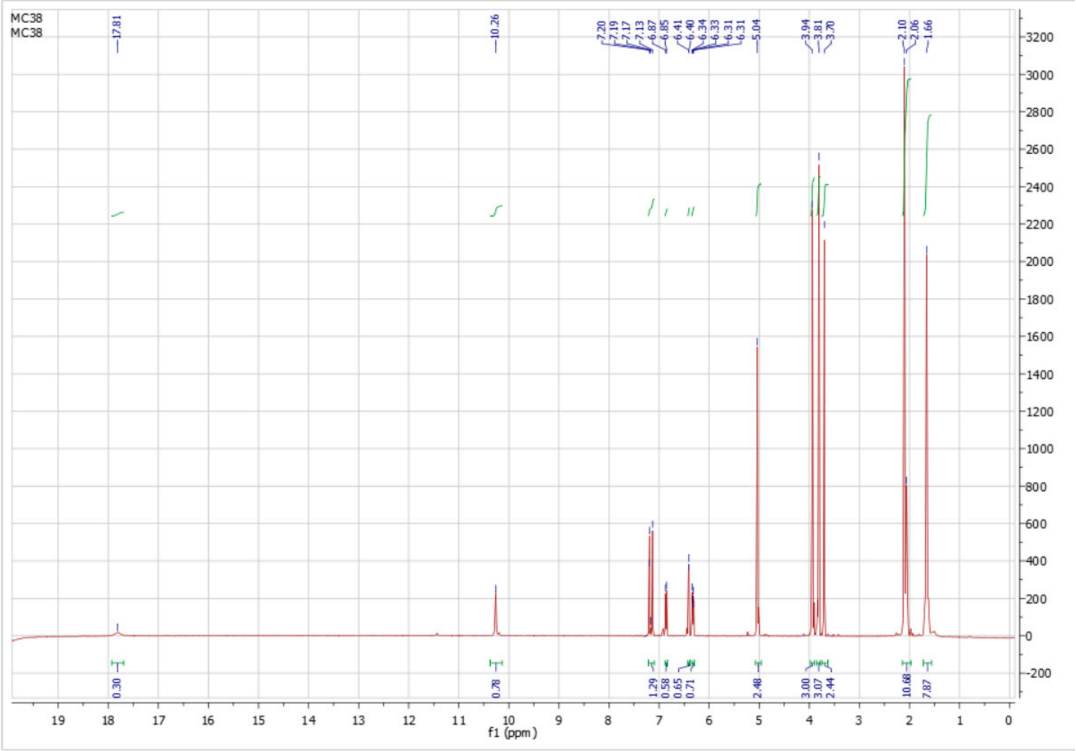

Figure S31. ^{1}H NMR spectrum of compound **27**.

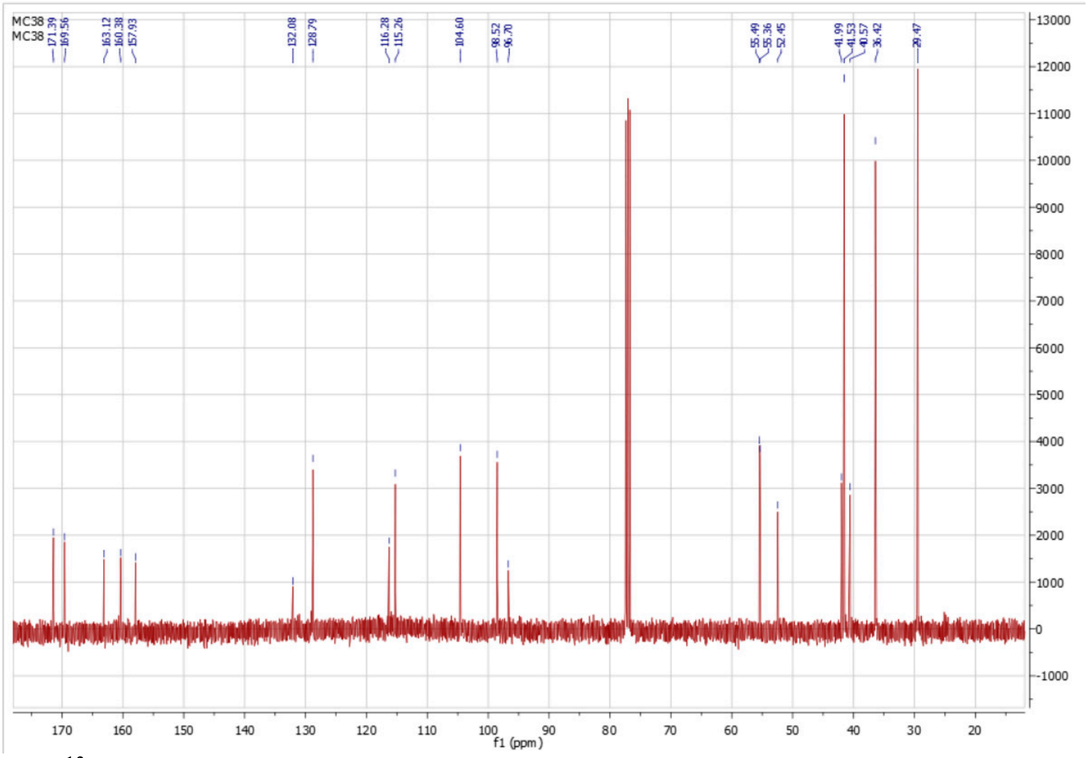

Figure S32. ^{13}C NMR spectrum of compound **27**.

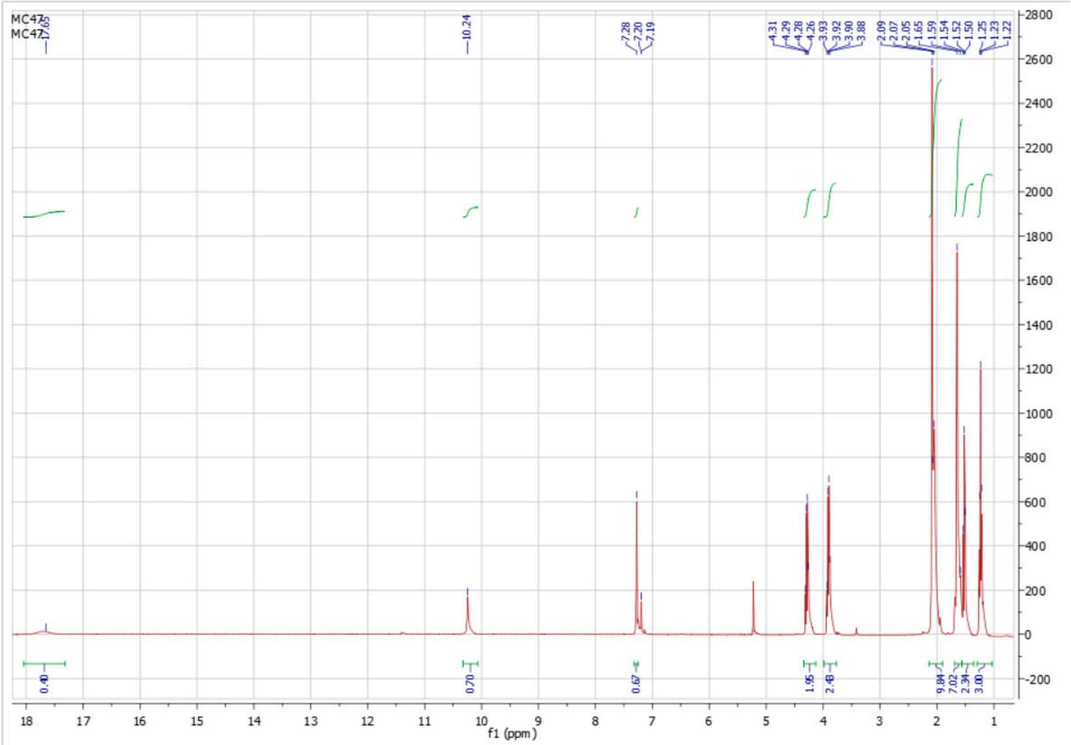

Figure S33. ^{1}H NMR spectrum of compound **33**.

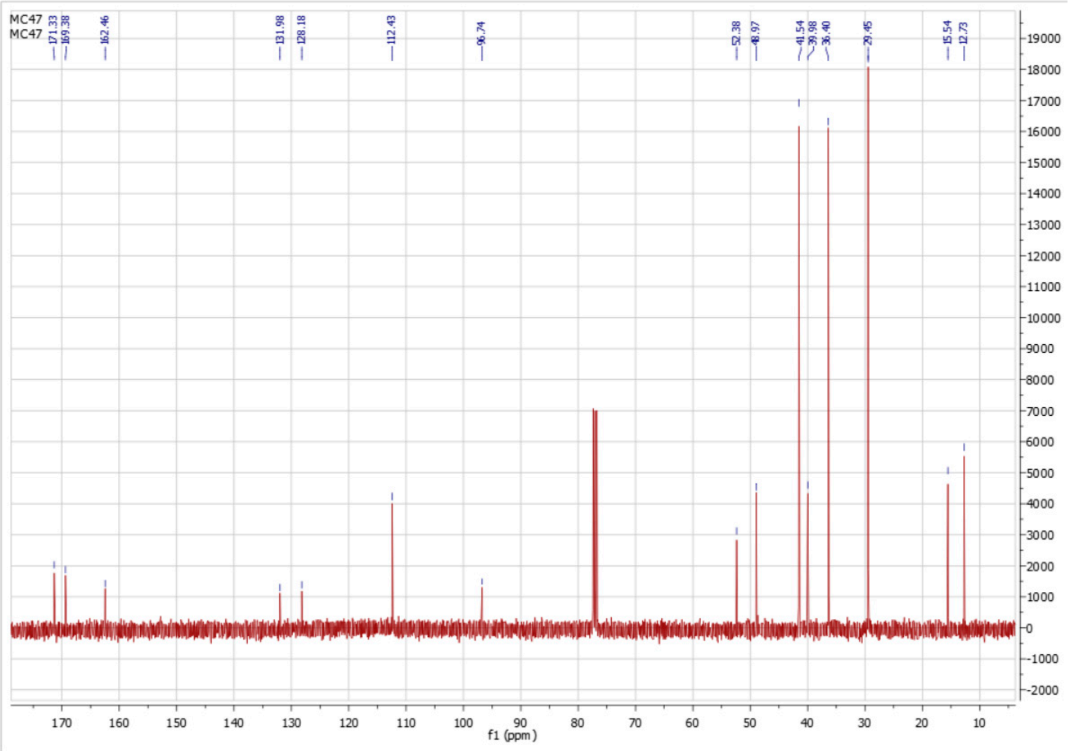

Figure S34. ^{13}C NMR spectrum of compound **33**.

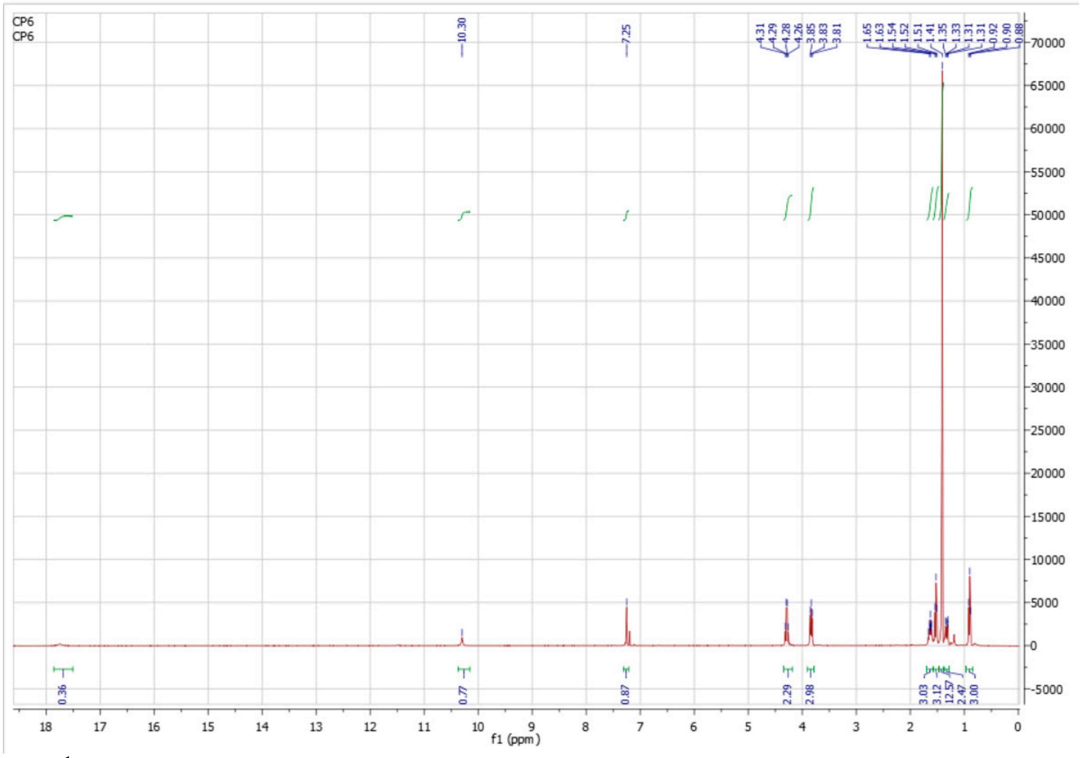

Figure S35. ^{1}H NMR spectrum of compound **34**.

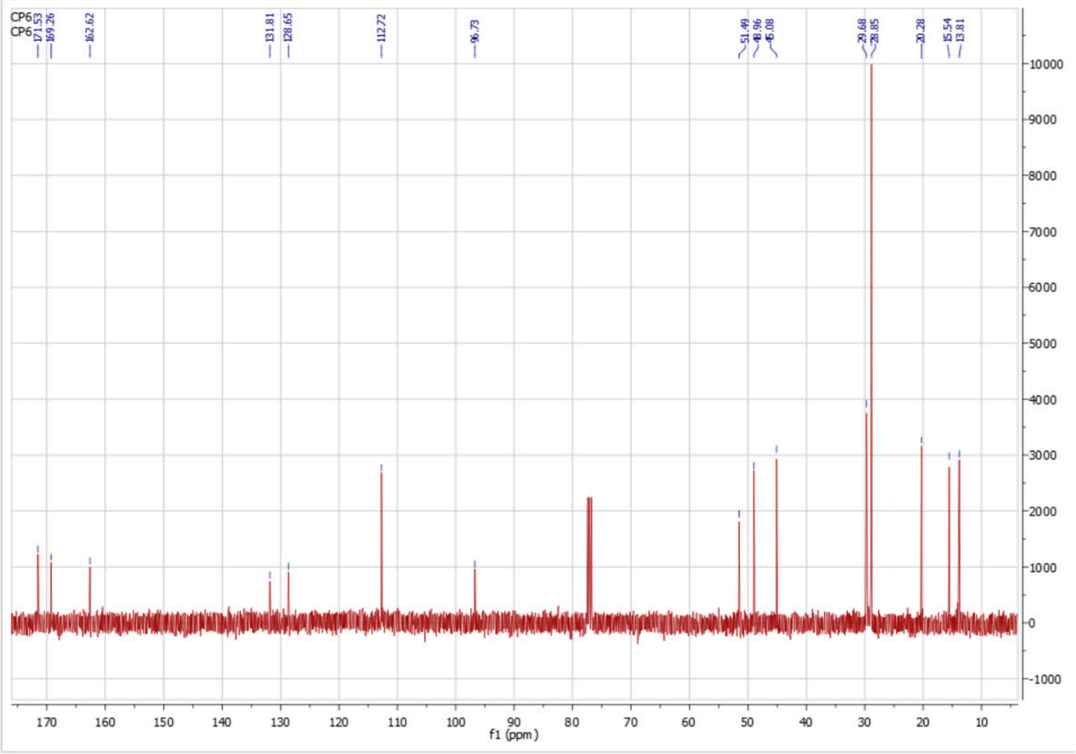

Figure S36. ^{13}C NMR spectrum of compound **34**.

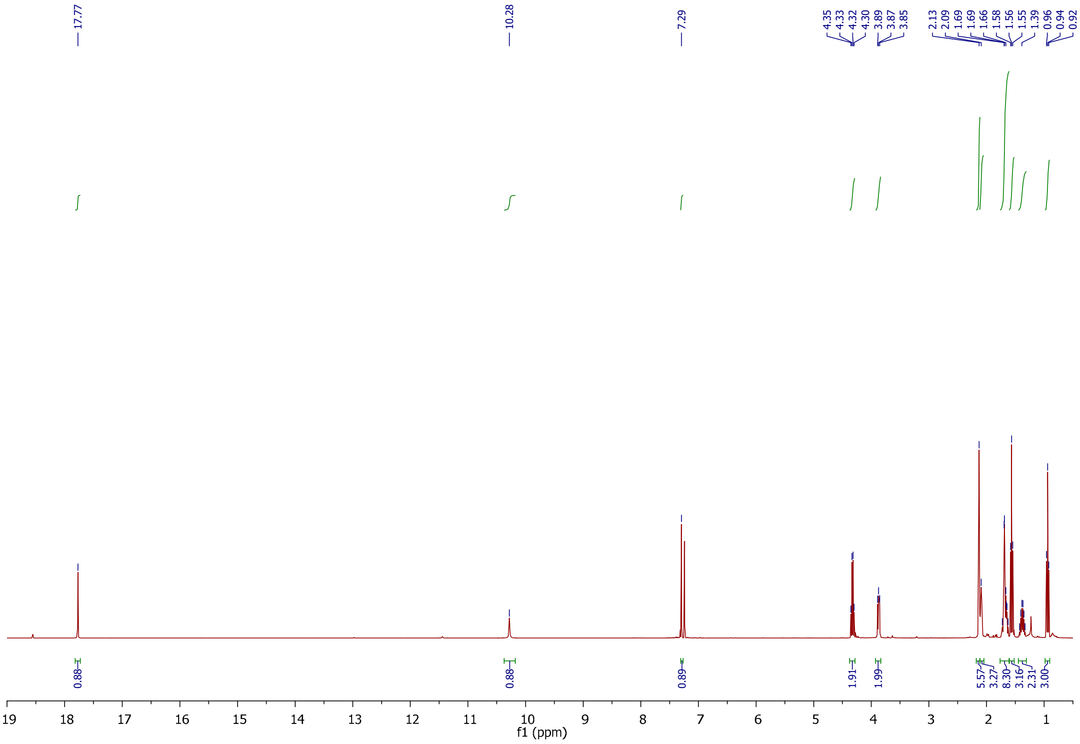

Figure S37. ^{1}H NMR spectrum of compound **35**.

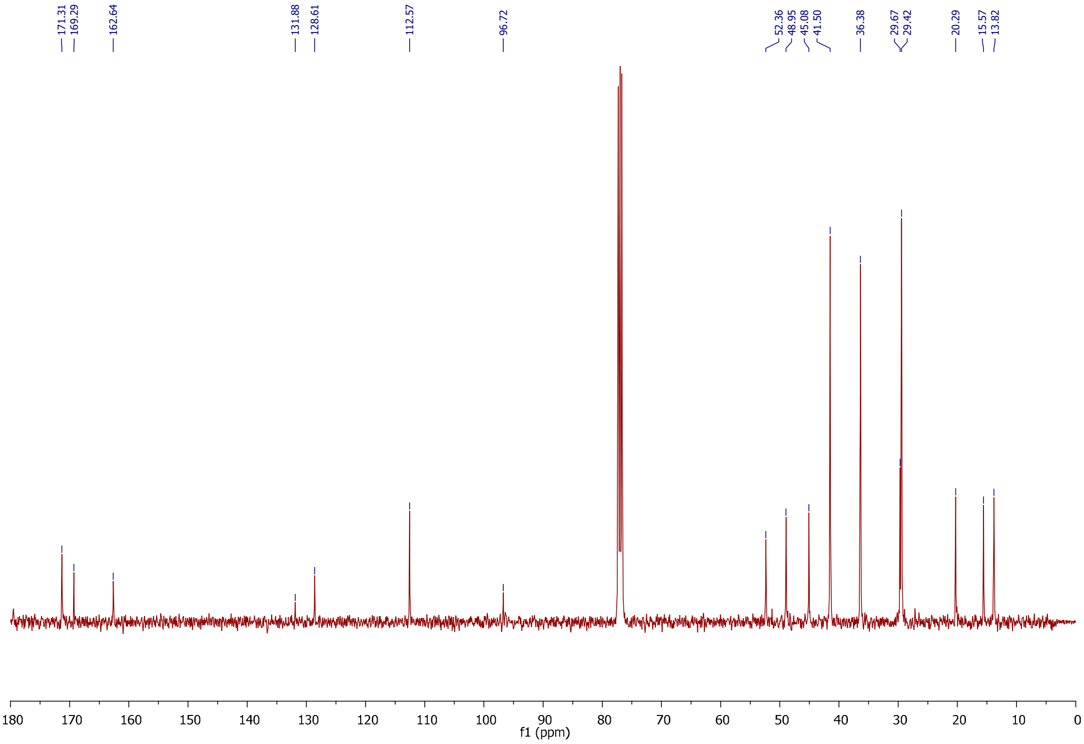

Figure S38. ^{13}C NMR spectrum of compound **35**.

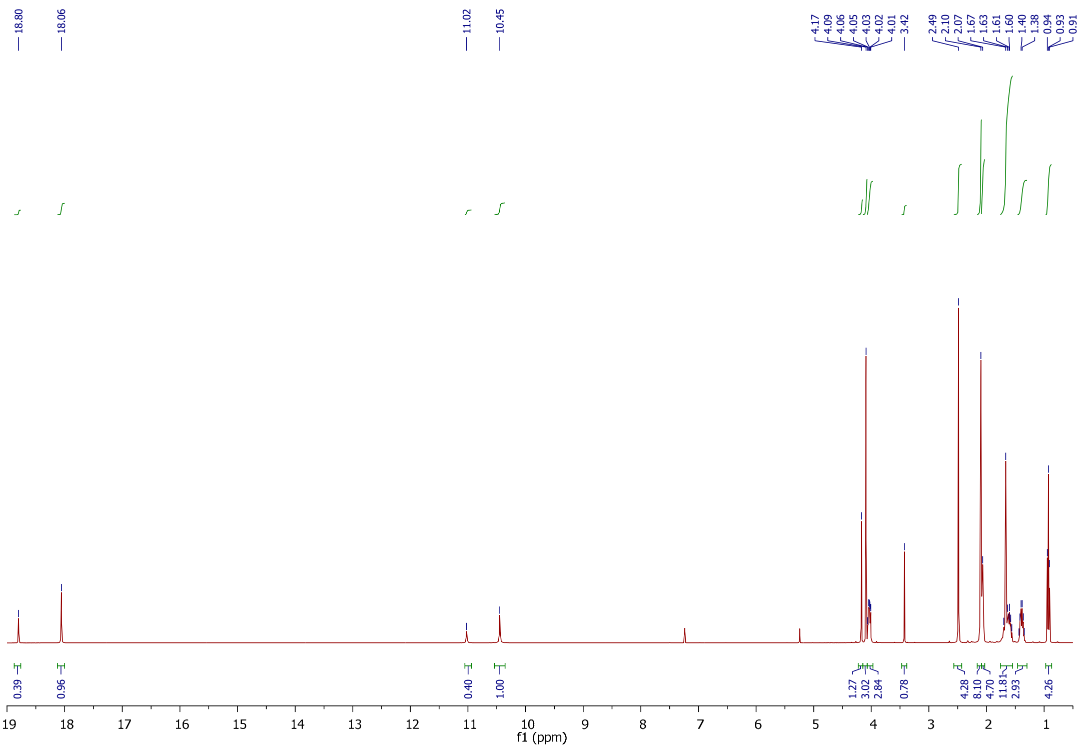

Figure S39. ^{1}H NMR spectrum of compound **51**.

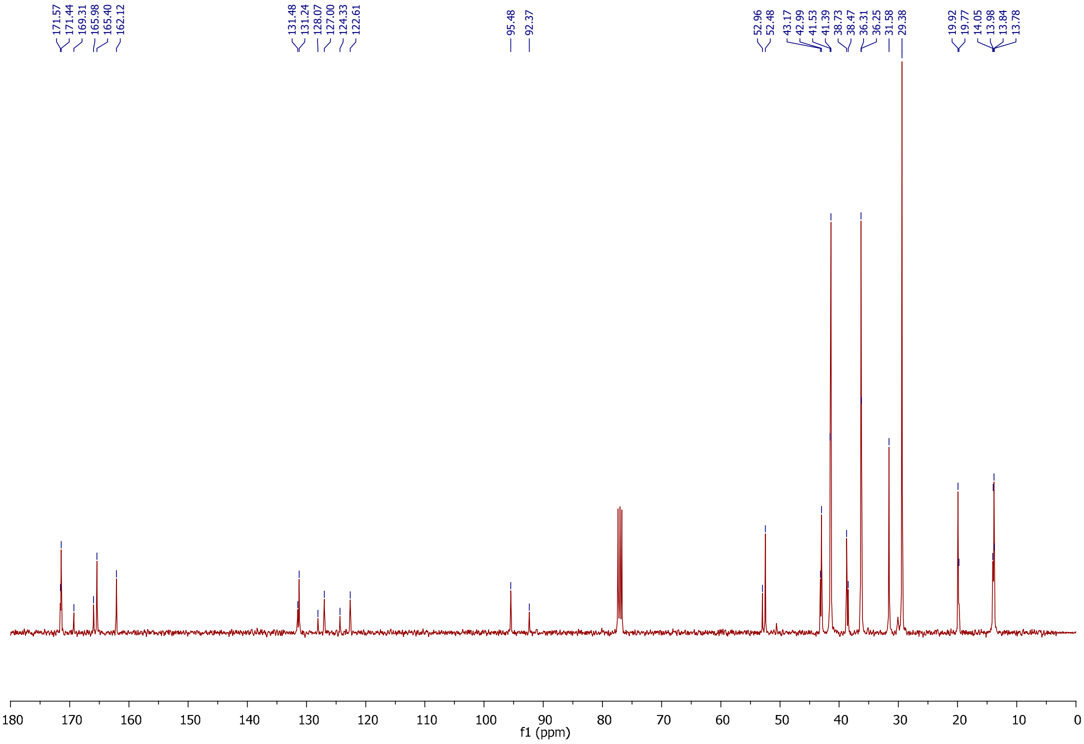

Figure S40. ^{13}C NMR spectrum of compound **51**.

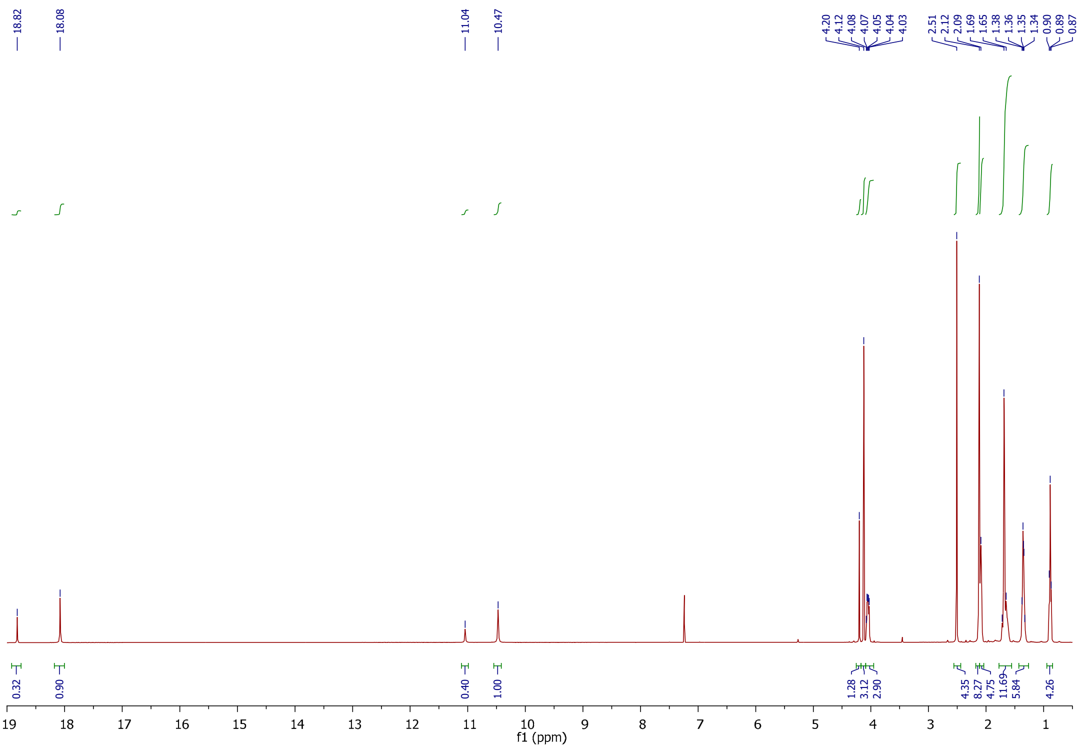

Figure S41. ^{1}H NMR spectrum of compound **52**.

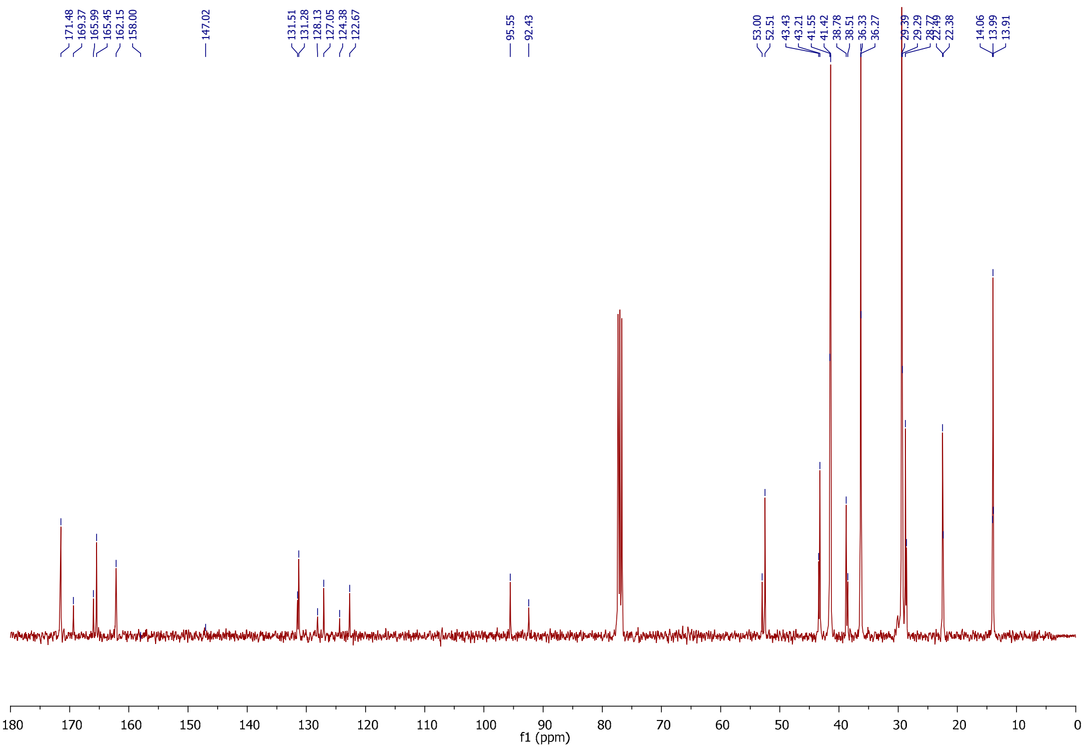

Figure S42. ^{13}C NMR spectrum of compound **52**.

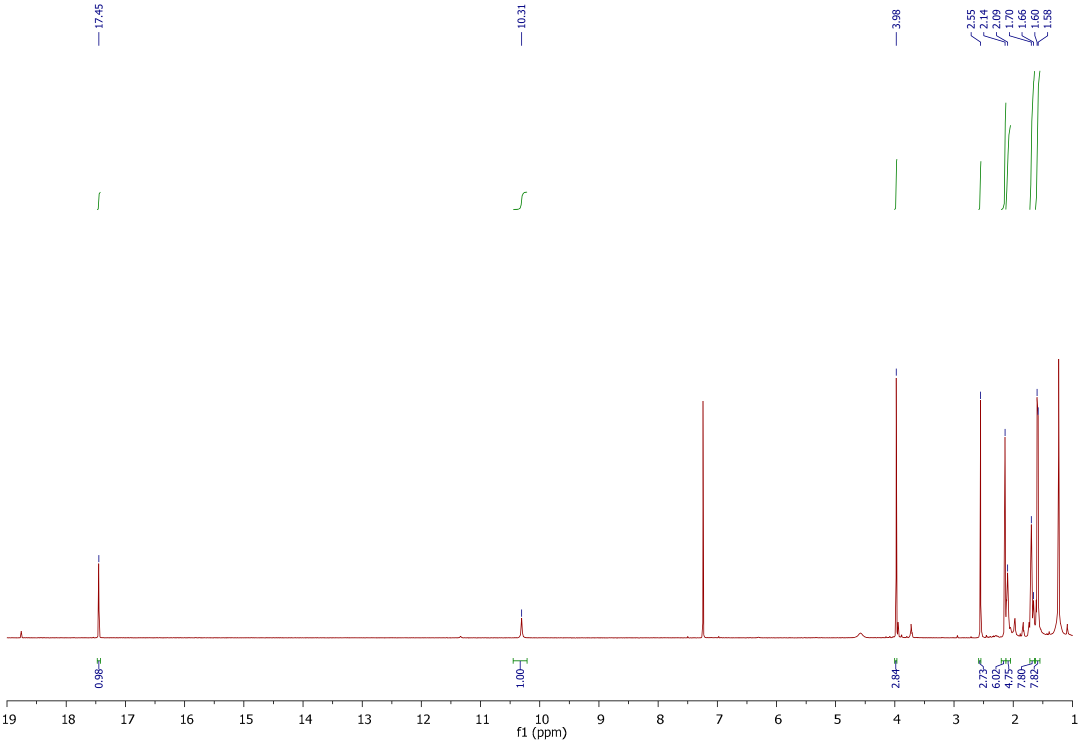

Figure S43. ^{1}H NMR spectrum of compound **53**.

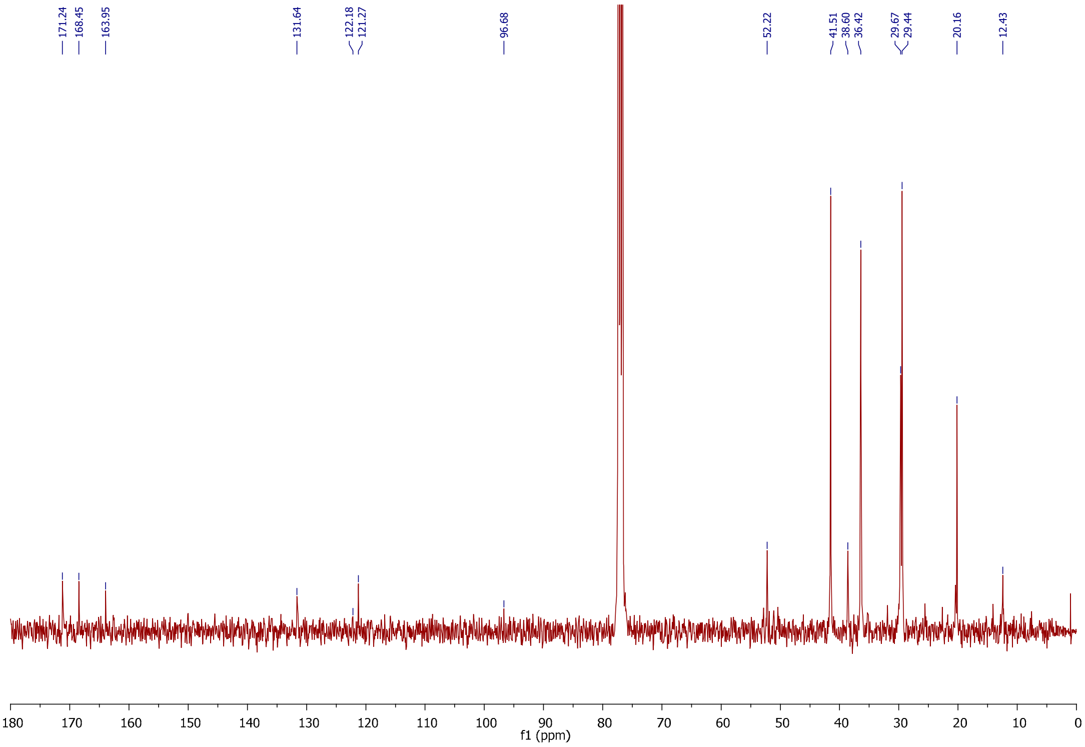

Figure S44. ^{13}C NMR spectrum of compound **53**.

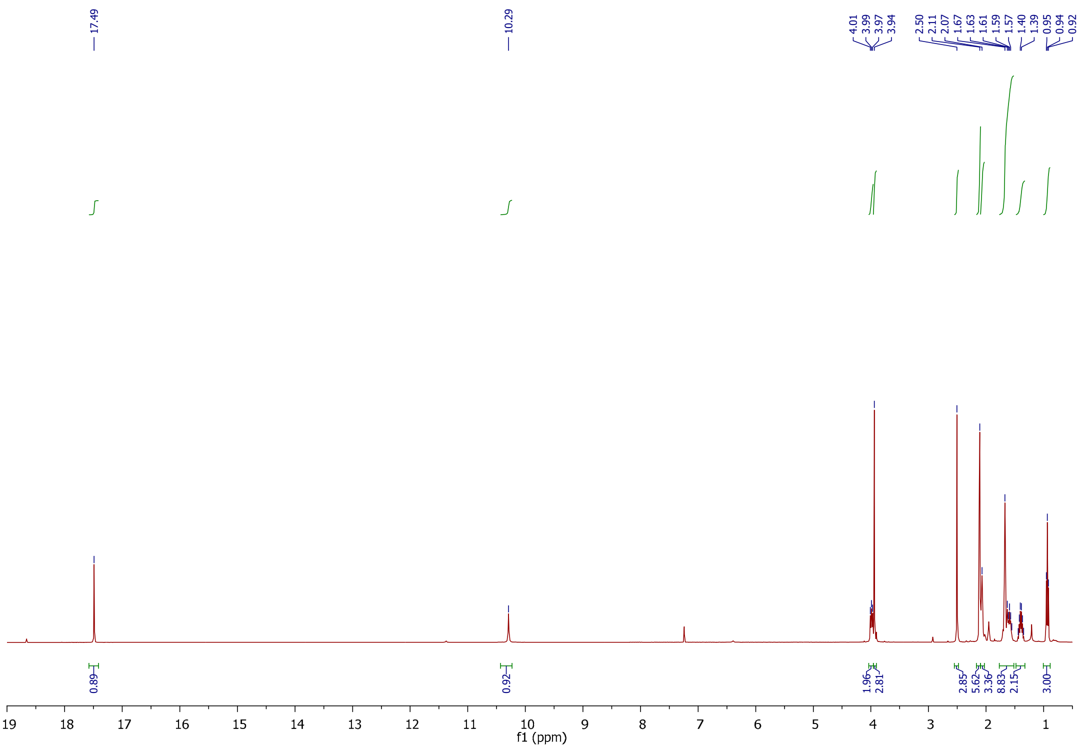

Figure S45. ^{1}H NMR spectrum of compound **54**.

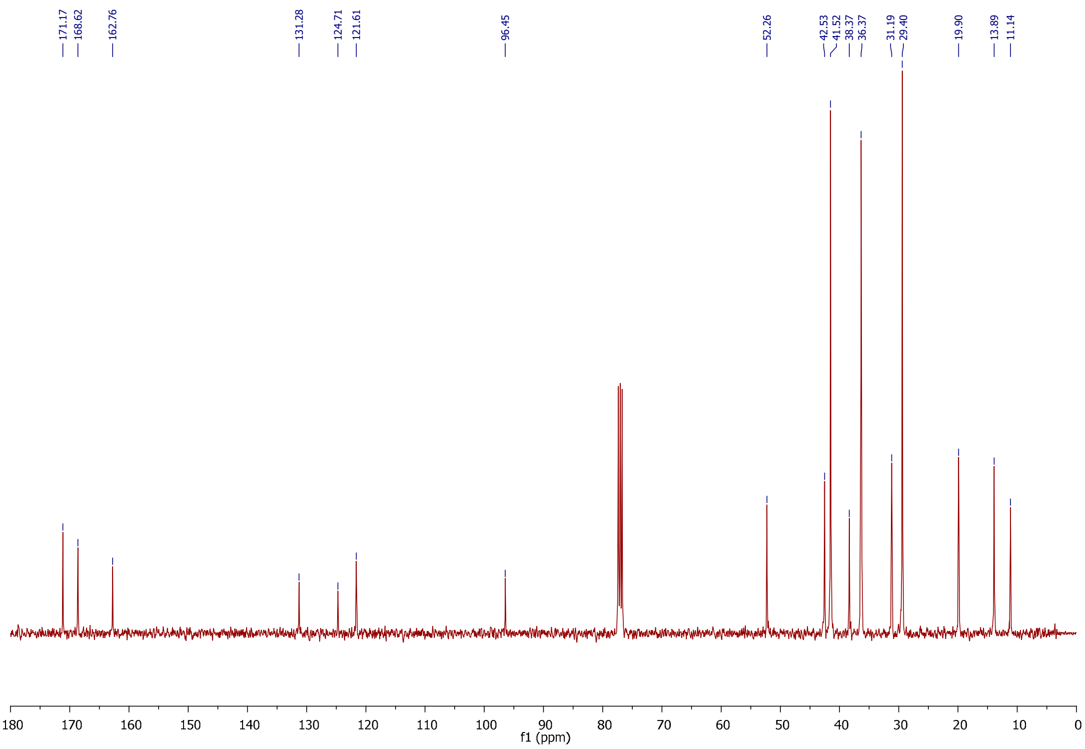

Figure S46. ^{13}C NMR spectrum of compound **54**.

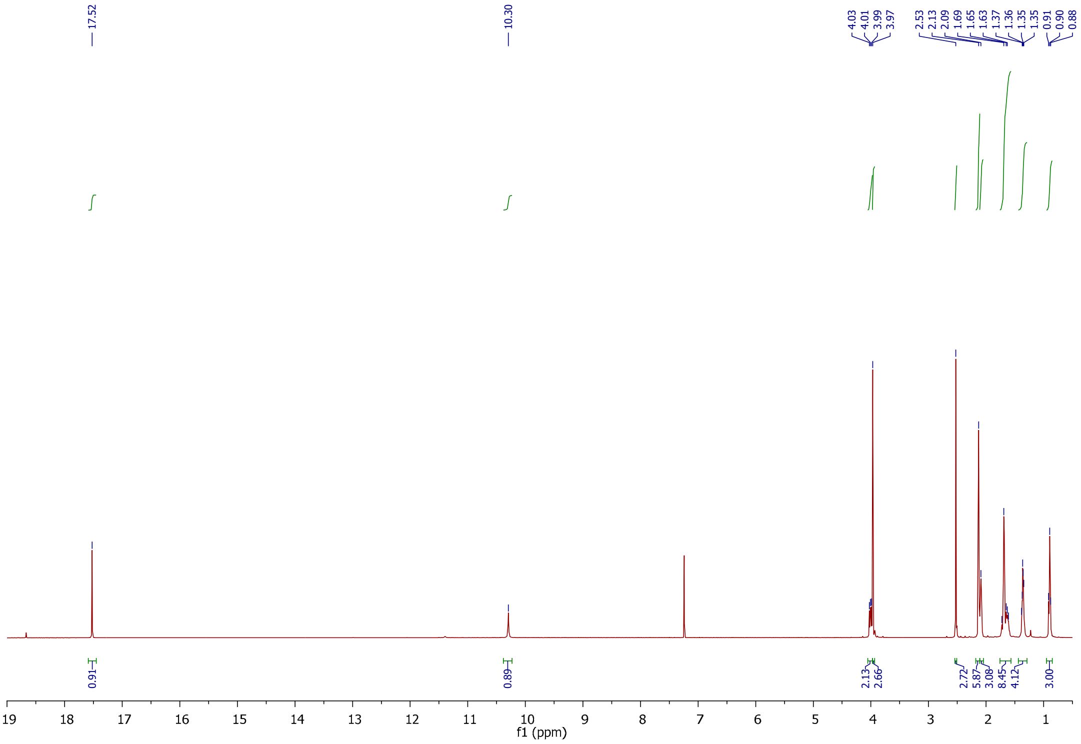

Figure S47. ^{1}H NMR spectrum of compound **55**.

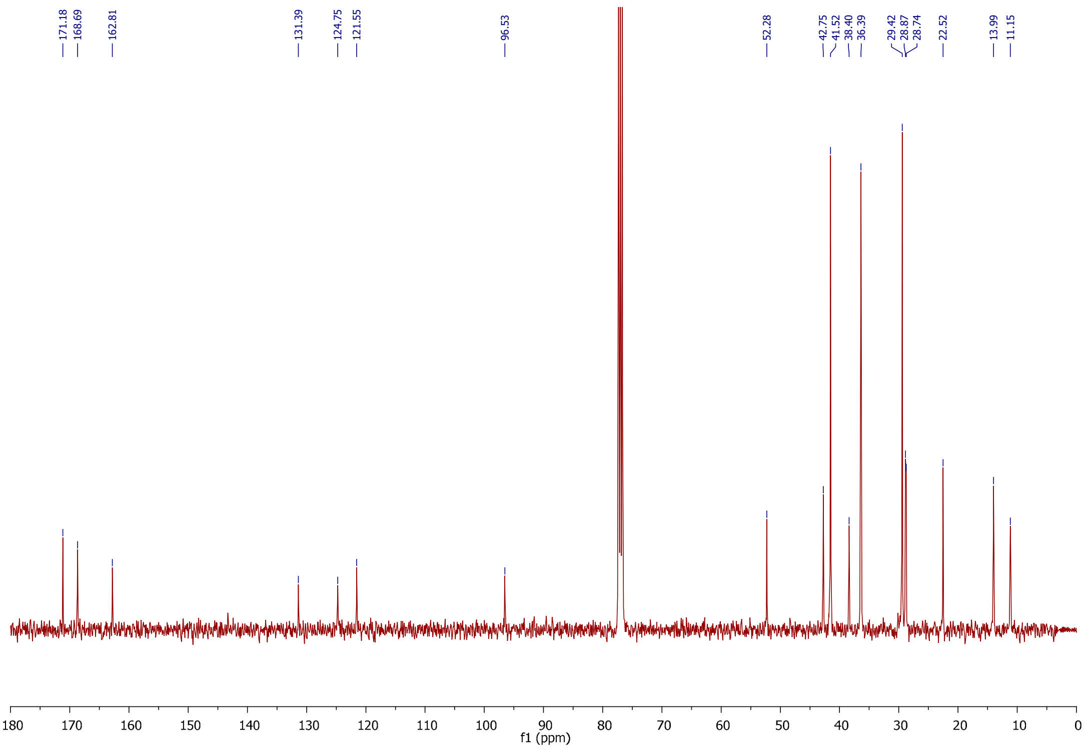

Figure S48. ^{13}C NMR spectrum of compound **55**.

Table S1. Calculated physicochemical and druglike properties of selected compounds

**Parameters**[a]	**Compounds**					
	**15**	**18**	**26**	**35**	**54**	**55**
No. H-bond acceptor	4	4	4	4	4	4
No. H-bond donor	2	2	2	2	2	2
Consensus Log$P_{o/w}$	2.88	2.86	3.11	3.21	3.20	3.53
No. rotatable bond	6	6	5	7	6	7
TPSA[b]	89.15	89.15	89.15	89.15	89.15	89.15
Lipinski's rule violation	No	No	No	No	No	No
PAINS alerts	No	No	No	No	No	No
Bioavailability score	0.55	0.55	0.55	0.55	0.55	0.55
GI absorption	High	High	High	High	High	High
Lipophilic Ligand Efficiency (LLE)[c]	5.16	4.90	6.21	6.14	5.47	5.15

[a]Properties calculated using the free web tool SwissADME [http://www.swissadme.ch]. [b]TPSA, topological polar surface area. [c]LLE, lipophilic ligand efficiency = pK_{iCB2} – CLogP.
